# Ancient genomic linkage of α-globin and Nprl3 couples metabolism with erythropoiesis

**DOI:** 10.1038/s41467-025-57683-z

**Published:** 2025-03-24

**Authors:** Alexandra E. Preston, Joe N. Frost, Megan R. Teh, Mohsin Badat, Andrew E. Armitage, Ruggiero Norfo, Sarah K. Wideman, Muhammad Hanifi, Natasha White, Noémi BA. Roy, Christian Babbs, Bart Ghesquiere, James Davies, Andrew JM. Howden, Linda V. Sinclair, Jim R. Hughes, Mira Kassouf, Rob Beagrie, Douglas R. Higgs, Hal Drakesmith

**Affiliations:** 1 MRC Translational Immune Discovery Unit, MRC Weatherall Institute of Molecular Medicine, John Radcliffe Hospital, University of Oxford, Oxford, UK; 2 Immunology Program, Memorial Sloan Kettering Cancer Center, New York, NY USA; 3 MRC Molecular Haematology Unit, MRC Weatherall Institute of Molecular Medicine, John Radcliffe Hospital, University of Oxford, Oxford, UK; 4 Centre for Genomics and Child Health, Blizard Institute, Queen Mary University of London and Barts Health, Whitechapel, London, UK; 5 Interdepartmental Centre for Stem Cells and Regenerative Medicine, Department of Biomedical, Metabolic and Neural Sciences, University of Modena and Reggio Emilia, Modena, Italy; 6 MRC Weatherall Institute of Molecular Medicine, John Radcliffe Hospital, University of Oxford, Oxford, UK; 7 Metabolomics Expertise Center, VIB Center for Cancer Biology, Katholieke Universiteit Leuven, Leuven, Belgium; 8 Oxford National Institute of Health Research Biomedical Research Centre, University of Oxford, Oxford, UK; 9 Division of Cell Signalling and Immunology, School of Life Sciences, University of Dundee, Dundee, UK; 10 Wellcome Centre for Human Genetics, University of Oxford, Oxford, UK; 11 Chinese Academy of Medical Sciences Oxford Institute, University of Oxford, Oxford, UK

**Keywords:** Differentiation, Genomics, Erythropoiesis

## Abstract

Red blood cell development from erythroid progenitors requires profound reshaping of metabolism and gene expression. How these transcriptional and metabolic alterations are coupled is unclear. *Nprl3* (an inhibitor of mTORC1) has remained in synteny with the α-globin genes for >500 million years, and harbours most of the a-globin enhancers. However, whether Nprl3 serves an erythroid role is unknown. We found that while haematopoietic progenitors require basal Nprl3 expression, erythroid Nprl3 expression is further boosted by the α-globin enhancers. This lineage-specific upregulation is required for sufficient erythropoiesis. Loss of Nprl3 affects erythroblast metabolism via elevating mTORC1 signalling, suppressing autophagy and disrupting glycolysis. Broadly consistent with these murine findings, human NPRL3-knockout erythroid progenitors produce fewer enucleated cells and demonstrate dysregulated mTORC1 signalling in response to nutrient availability and erythropoietin. Therefore, we propose that the anciently conserved linkage of NprI3, α-globin and their associated enhancers has coupled metabolic and developmental control of erythropoiesis.

## Abstract (web summary)

Nprl3 controls erythroblast metabolism and is upregulated by α -globin enhancers sited within its introns. This arrangement couples metabolic and developmental control during erythropoiesis and may explain the ancient synteny of α -globin with Nprl3.

## Introduction

Human erythropoiesis produces ~2 million erythrocytes per second^1^, representing the highest synthesis of all cell types^2^, and likely the largest continuous metabolic challenge in the body. Erythropoiesis also involves tightly controlled transcriptional regulation of the α- and β-globin genes, ensuring that globin synthesis occurs in a timely and sufficient manner. Regulation of α-globin expression by its enhancers has been extensively characterised^3–7^. Interestingly, α-globin has been maintained in synteny with Nitrogen permease regulator-like 3 (*Nprl3*) since before the divergence of jawed (gnathostome) and jawless (agnathan) vertebrates, and before the teleost-specific genome duplication. The α- and β-globin gene clusters separated in amniote vertebrates due to transposition of the proto β-globin gene^8^. However, *Nprl3*, α-globin and the α-globin enhancers have been conserved in synteny for >500 million years^9^, leading us to hypothesise that their co-localisation underlies a functionally significant partnership between the genomic elements.

*Nprl3* is conserved throughout Animalia, Fungi, Excavata, SAR (Stramenopiles, Alveolates, Rhizaria) and Amoebazoa; the only taxon that evidently lacks an *Nprl3*-like gene is that of green algae and land plants^8^. *Nprl3* is broadly expressed across vertebrate cell types, and its loss is embryonic lethal^10^. Nprl3 is a member of the GATOR-1 complex, which negatively regulates mTORC1. mTORC1 is a central metabolic controller, promoting anabolic processes (such as protein translation and synthesis^11^), inhibiting catabolism (such as autophagy^12^), and influencing metabolic pathways of ATP production^13^. Generally, anabolic metabolic outputs require active mTORC1, whereas catabolic signals occur under low mTORC1 signalling. mTORC1 serves critical roles in erythroid cells, contributing to an essential anabolic-catabolic balance during erythropoiesis: erythroblasts undergo rapid haemoglobinisation followed by high autophagic and proteasomal activity to expel or degrade organelles, ultimately resulting in a cytosol comprising 98% haemoglobin^14,15^. Nevertheless, how mTORC1 is dynamically regulated during this process remains poorly understood, and to the best of our knowledge, the role of Nprl3 in erythropoiesis has not been explored.

**Fig. 1:**
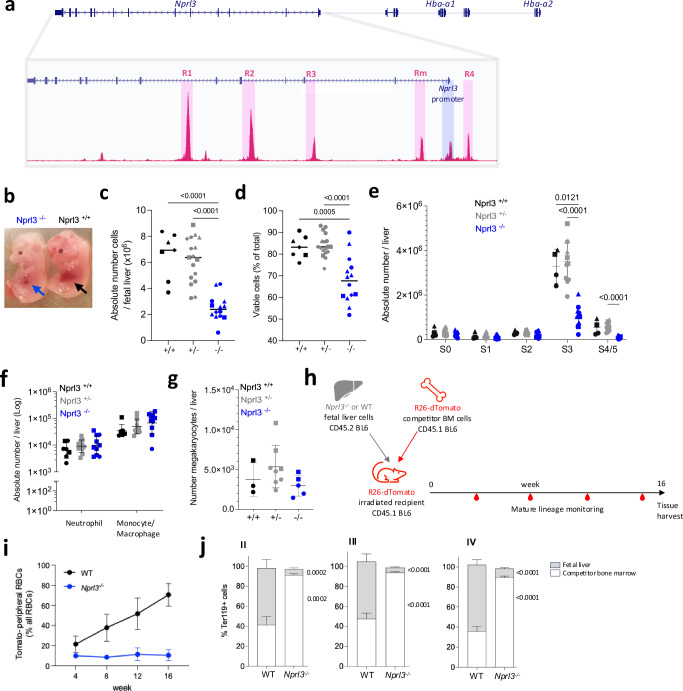
Loss of Nprl3 impairs erythroid differentiation in murine foetal liver and adult competitive chimaeras. **a** Schematic illustrating the relative genomic localisation of *Nprl3* and *Hba-1/2*, as well as the positions of the 5 murine α-globin enhancers, MCS-R1, R2, R3, Rm and R4, defined here by ATAC-Seq (GSE174110). **b** Representative images of E13.5 *Nprl3*^*−/−*^ and *Nprl3*^*+/+*^ embryos. **c** Total number and (**d**) viability of cells per foetal liver according to genotype (5 litters. Each point represents a foetal liver, and each shape represents a litter). Analysed by One-way ANOVA followed by Tukey’s test. **e** Absolute number of erythroid cells in stages S0-S5 of differentiation in foetal livers of *Nprl3*^*+/+*^*, Nprl3*^*+/−*^ and *Nprl3*^*−/−*^ embryos (*n* = 3 litters. Each point represents a foetal liver, and each shape represents a litter). S0/S1, CFU-Es; S2, proerythroblasts; S3, basophilic erythroblasts; S4/S5, polychromatic erythroblasts-enucleated cells. Points indicate individual embryos. Mean ± SD indicated. Analysed by Two-way ANOVA followed by Tukey’s test. **f** Absolute numbers of CD11b+ Ly6G+ neutrophils and CD11b+ Ly6G- F4/80+ monocytes/macrophages (*n* = 3 litters. Each point represents a foetal liver, and each shape represents a litter). Data have been log-transformed for visualisation and statistical analysis. Each point represents an individual embryo, and the geometric mean ± geometric SD are indicated. Analysed by Two-way ANOVA followed by Tukey’s test. **g** Absolute number of c-kit- CD41+ ‘megakaryocyte’ cells per foetal liver (*n* = 2 litters. Each point represents a foetal liver, and each shape represents a litter). Mean ± SD are indicated, and the data was analysed by One-way ANOVA followed by Tukey’s test. **h** Schematic illustration of the competitive chimaera experimental design. **i** Proportion of peripheral RBCs derived from WT and *Nprl3*^*−/−*^ foetal liver cells (each point represents 8 mice). Data are expressed as mean ± SD. **j** Competitivity comparison showing the proportions of cells in each erythroblast stage (defined by flow cytometry using CD44 and forward scatter: II, basophilic; III, polychromatic, and IV, orthochromatic erythroblasts) derived from foetal liver vs. competitor bone cells, according to the genotype of the transplanted foetal liver. Analysed in recipient bone marrow at week 16 post-transplantation. Data analysed by One-way ANOVA followed by Šídák’s test (*n* = 8 mice per group). Upper *p*-value compares foetal liver, lower compares bone marrow competitors. Data are expressed as mean ± SD. Nprl3 nitrogen permease regulator 3, Hba alpha-globin, MCS multispecies conserved sequence, CFU-E colony-forming unit erythroid, SD standard deviation. Source data are provided as a Source Data file.

*Nprl3* introns contain 4 of the 5 α-globin enhancers in mice, and 3 of 4 in humans (Fig. 1a)^16^. Furthermore, published data show that *Nprl3* RNA expression increases during erythroid commitment^17^. Considering its conserved genomic context and function regulating mTORC1, we aimed to explore the hypothesis that Nprl3 serves a key role in erythroid metabolism, potentially transcriptionally supported by its location in a transcriptional hub, thus linking metabolism with completion of the erythroid programme during red blood cell synthesis.

This study presents a critical role for Nprl3 in erythropoiesis, and shows that *Nprl3* expression levels are regulated by the α-globin enhancer cluster. Further findings suggest that the transcriptional hub (formed by *Nprl3*, α-globin and the associated enhancers) is functionally beneficial to erythroid cells.

## Results

### Nprl3-deficient erythropoiesis is ineffective in foetal and adult mice

We employed an *Nprl3* constitutive knockout (KO) model (*Nprl3*^−/−^), in which the entire *Nprl3* promoter region is deleted, leaving the α-globin enhancers (MCS- (multispecies conserved sequence) R1, R2, R3, Rm and R4) unchanged^18^. This deletion is embryonic lethal when homozygous (between embryonic day (E)15 and birth)^19^. In this model, *Nprl3*^−/−^ foetal liver demonstrates severe impairment of erythropoiesis compared to littermate controls. This is evident from visual inspection of embryos: healthy foetal liver at E13.5 is mostly comprised of haemoglobinising erythroblasts, whereas inability to fulfil this development in *Nprl3*^−/−^ embryos results in reduced foetal liver size (Fig. 1b), cellularity (Fig. 1c) and cell viability (Fig. 1d). Staging of erythroid differentiation based on Ter119/CD71 expression^20^ showed a profound block on terminal erythroid differentiation, with significantly fewer *Nprl3*^−/−^ erythroblasts reaching stage 3 (S3) (basophilic erythroblasts) compared to WT (Fig. 1e and Supplementary Fig. 1a, b). Notably, myelopoiesis and megakaryopoiesis are unimpaired in *Nprl3*^−/−^ foetal liver (Fig. 1f, g), showing an erythroid-specific dependence on sufficient *Nprl3* expression. Common myeloid progenitors (CMPs) and megakaryocyte erythroid progenitors (MEPs) were both reduced in number in the *Nprl3*^*−/−*^ foetal liver, whereas granulocyte monocyte progenitors (GMPs) and c-kit^+^Sca-1^+^ haematopoietic stem and progenitor cells (HSPCs) were unchanged (Supplementary Fig. 1c; measured by flow cytometry^21^, example Supplementary Fig. 5b). This is indicative of a biased erythroid/megakaryocyte sensitivity to *Nprl3*^*−/−*^, among haematopoietic progenitors, in addition to later-stage inhibition of erythropoiesis.

To assess the effects of *Nprl3*^−/−^ in adult erythropoiesis, independent of any potential systemic developmental defects, a foetal liver-bone marrow competitive chimaera model was established (Fig. 1h). One group of lethally irradiated CD45.1 *R26-*^*tdTomato*^ mice were adoptively transferred *Nprl3*^−/−^ CD45.2 foetal liver cells alongside *R26-*^*tdTomato*^ WT bone marrow cells, and another received WT CD45.2 foetal liver cells alongside *R26-*^*tdTomato*^ WT bone marrow cells. Equal numbers of foetal liver cells, and equal numbers of competitor bone marrow cells, were transferred between groups. As erythroid cells lose expression of CD45 antigens with maturity, the *R26-*^*tdTomato*^ ubiquitous reporter construct enabled the origin of mature RBCs to be defined in reconstituted mice as foetal liver (*Tomato*^*−*^*)* or bone marrow competitor (*Tomato*^*+*^).

Over 16 weeks of reconstitution, *Nprl3*^*−/−*^ foetal liver-derived cells contributed poorly to the peripheral RBC population (Fig. 1i). After 16 weeks, we determined the contribution of *Nprl3*^*−/−*^ cells through successive stages of bone marrow erythropoiesis, which showed a profound defect in terminal erythroid differentiation. *Nprl3*^*−/−*^ cells comprised only 5–10% of basophilic, polychromatic and orthochromatic erythroblasts in the bone marrow, whereas WT foetal liver-derived cells comprised ~60% (Fig. 1j and Supplementary Fig. 1d; assessed by flow cytometry, as per Supplementary Fig. 1e^22^). *Nprl3*^*−/−*^ cells seemed to accumulate in the pro-erythroblast stage where their contribution was comparable to that of WT foetal liver-derived cells (Supplementary Fig. 1f), suggesting a differentiation block reminiscent of our observations in the foetal liver (Fig. 1e).

In addition to defects in terminal erythropoiesis, we observed a competitive disadvantage at the earliest assessed haematopoietic bifurcation: fewer MPPs were formed by *Nprl3*^−/−^ foetal liver cells (Supplementary Fig. 1f; haematopoietic progenitors were defined by flow cytometry using a previously characterised scheme^23^, see Supplementary Fig. 5). This appeared to result in pan-haematopoietic disadvantage; *Nprl3*^*−/−*^ foetal liver contributed poorly to most progenitor cell types (except CFU-E/ProE) and mature haematopoietic cell types (Supplementary Fig. 1f, g). This is not reflective of the comparatively normal numbers of non-erythroid cell types and their progenitors found in E13.5 *Nprl3*^−/−^ foetal liver (Fig. 1f, g). Notably, this HSC-level disadvantage was only observed in the competitive setting, where more ‘fit’ WT cells exist in the same haematopoietic system. The difference between these settings may also reflect that foetal liver erythropoiesis is driven by progenitors that develop independently of the HSC compartment, whereas adult bone marrow haematopoiesis is HSC-dependent^24^. Interestingly, HSCs and erythroid cells express the highest levels of *Nprl3* among haematopoietic cells in the mouse bone marrow (Supplementary Fig. 1h) and it is known that HSC fitness is regulated by appropriate levels of mTORC1 signalling^25,26^.

Irrespective of the potential role for *Nprl3*^−/−^ in HSCs, the accumulation of *Nprl3*^*−/−*^ pro-erythroblasts and subsequent defect in terminal erythropoiesis supports a role for *Nprl3*^−/−^ in erythroid differentiation. The chimaeras indicate that the *Nprl3*^*−/−*^ erythroid deficiency is haematopoietic intrinsic, and independent of systemic developmental defects in *Nprl3*^−/−^ embryos.

### Human *NPRL3*-KO erythroblasts respond defectively to a changing cellular environment

**Fig. 2:**
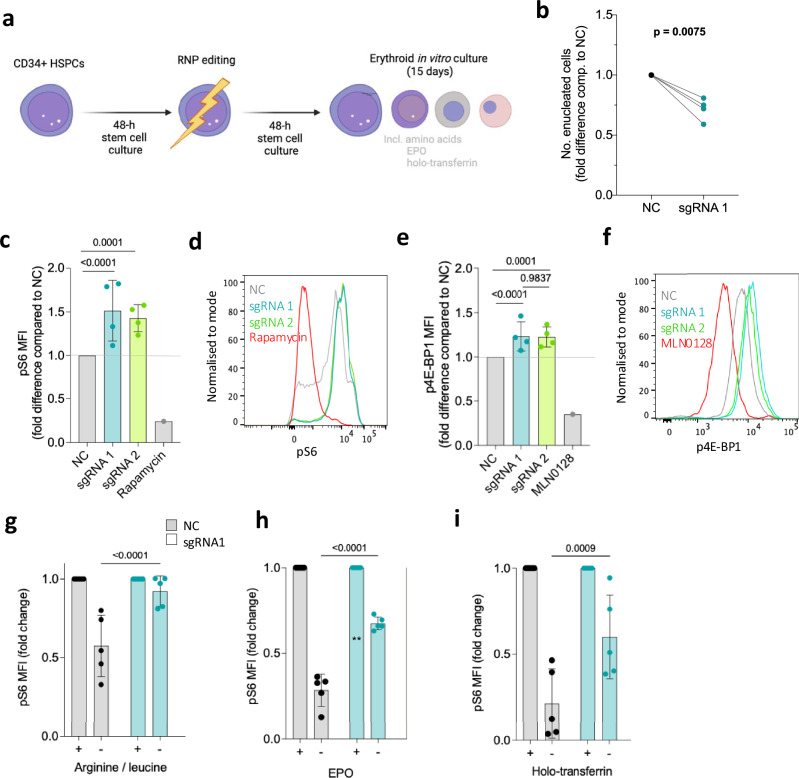
NPRL3-KO impairs primary human in vitro erythroid differentiation and erythroblast mTORC1 signalling. **a** Schematic illustration: following extraction of human peripheral blood mononuclear cells, magnetic assisted cell sorting was used to isolate CD34+ HSPCs. After a 48-h rest and expansion period, RNP-editing was performed to knockout NPRL3. After another rest period, the edited cells were exposed to an erythroid differentiation culture. Created in BioRender.com ‘Preston, A. (2025) https://BioRender.com/y65h405’. **b** Fold difference in the average number of enucleated cells formed by NPRL3-KO progenitors compared to NC by day 14. NC (negative control) represents a scrambled non-targeting sgRNA. Four individual donors are indicated, and each pair of connected points represents the average from one individual, and each average is represented by the geometric mean. Analysed by one-tailed ratio paired *t*-test. **c** pS6 median fluorescent intensity (MFI) of NC cells compared to NPRL3-KO induced using sgRNA1/2, measured by flow cytometry on day 11, 24 h post-media change (*n* = 4 cell populations from four separate donor individuals), also represented in (**d**) using a histogram. **e** p4E-BP1 MFI on day 11 (*n* = 4 cell populations from four separate donor individuals), also represented in (**f**) using a histogram. Rapamycin and MLN0128 were included to indicate the dynamic scope of mTORC1 signalling measurable by pS6 MFI. Error bars represent standard deviation. Fold reduction in pS6 MFI upon 4-h withdrawal of (**g**) arginine and leucine, (**h**) EPO and (**i**) holo-transferrin, measured on day 11. Each point represents an individual donor (*n* = 4 cell populations from four separate donor individuals). Analysed by Two-way ANOVA followed by Tukey’s test. Error bars represent standard deviation. NPRL3 nitrogen permease regulator 3, mTOR mammalian target of rapamycin, HSPC hematopoietic stem and progenitor cell, RNP ribonucleoprotein, NC negative control, KO knockout, pS6 phosphorylated S6, p4E-BP1 eukaryotic translation initiation factor 4E-binding protein 1, EPO erythropoietin, sgRNA short guide RNA, MFI median fluorescent intensity, SD standard deviation. Source data are provided as a Source Data file.

Homozygous *NPRL3* loss-of-function mutations have not been identified in humans, likely due to early lethality. To study NPRL3 in human erythropoiesis, we established an in vitro model of *NPRL3*-KO in erythroid cells. High-efficiency *NPRL3*-KO was induced in primary human CD34+ HSPCs by RNP-editing (Fig. 2a) and maintained throughout 15 days of erythroid-directed culture (Supplementary Fig.  2a, b)^27,28^. Negative control (NC) cells were nucleofected with a scrambled non-targeting sgRNA—Cas9 complex. To assess the ultimate erythroid productivity of single *NPRL3*-KO CFU-Es versus single NC CFU-Es, these cells were singly sorted into individual wells prior to erythroid culture. *NPRL3*-KO CFU-Es formed significantly fewer enucleated cells compared to their NC counterparts by day 15 (Fig. 2b and Supplementary Fig. 2c), showing that loss of NPRL3 also impairs the output of human in vitro erythropoiesis. Monitoring of cell number during bulk cell culture indicated a proliferative burst in NC populations from day 7 of culture, resulting in a peak in cell number on day 9 (Supplementary Fig.  2d). This was not observed in *NPRL3*-KO populations, suggesting a growth or differentiation impairment of *NPRL3*-KO erythroblasts. The culture is mostly comprised of basophilic erythroblasts at this time, and so this result is consistent with the loss of progression to S3 observed in murine *Nprl3*^*−/−*^ foetal liver (Fig. 1e).

On day 11 of the CD34 erythroid culture, bulk cultured *NPRL3*-KO erythroblasts (mostly basophilic and polychromatic erythroblasts) also showed overactive mTORC1 signalling, as measured by phosphoS6 (pS6; (Fig. 2c, d) and p4EB-P1 (Fig. 2e, f), close downstream effectors of mTORC1. This confirmed that NPRL3 deficiency results in increased mTORC1 activity by removing a means of negative mTORC1 regulation, demonstrating canonical GATOR-1 signalling in human erythroid cells for the first time. In cell lines and *Drosophila*, NPRL3 (as part of GATOR-1), inhibits mTORC1 activity in response to low amino acid availability, particularly arginine and leucine^29–31^. To test human erythroid cultures, edited erythroblasts were exposed to leucine and arginine withdrawal on day 11 for 4 h before pS6 measurement. In such conditions, *NPRL3*-KO erythroblasts were unable to appropriately downregulate mTORC1 activity (Fig. 2g). Two key physiologic regulators of erythropoiesis are iron availability and erythropoietin (EPO). Interestingly, *NPRL3*-KO erythroblasts failed to appropriately regulate pS6 in response to iron deficiency and EPO deprivation (separately, in replete amino acid conditions; Fig. 2h, i). This shows that NPRL3 is required for appropriate integration of multiple stimuli that influence successful completion of erythropoiesis.

These results highlighted the importance of the NPRL3 - mTORC1 axis as a central metabolic regulator of erythropoiesis. We next revisited the foetal liver model to more closely assess erythroid cell metabolism.

### Nprl3 maintains the metabolic profile of erythroid cells

**Fig. 3:**
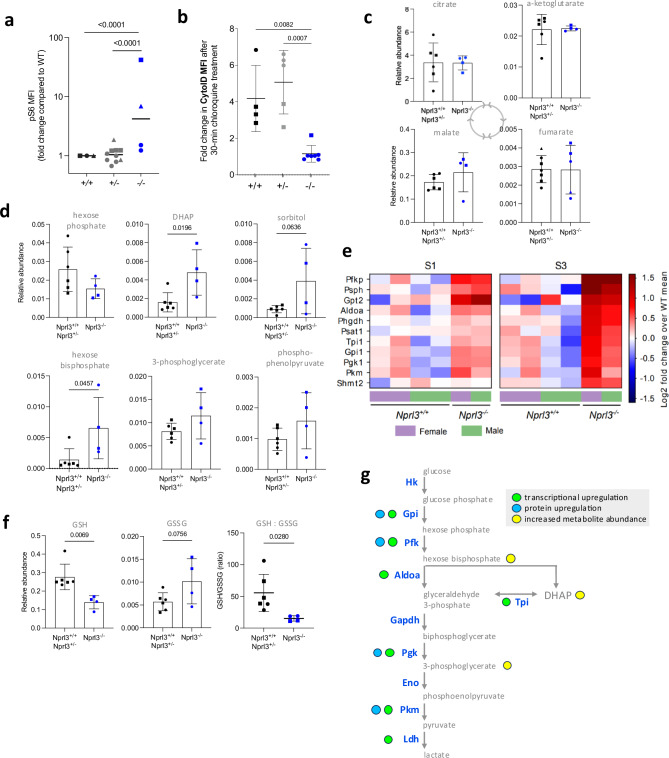
Metabolic effects of *Nprl3*^*−/−*^ in E13.5 foetal liver. **a** pS6 MFI measured in S2 foetal liver erythroblasts (fold change compared to *Nprl3*^*+/+*^), following a 2-h 37 °C incubation in RPMI with 5% FCS (otherwise un-supplemented). Each point indicates a foetal liver, symbols indicate different litters (*n* = 3 litters). Analysed by Two-way ANOVA followed by Tukey’s test (grouped by litter. High baseline variability between litters (i.e. experiments) could be due to temporal instrumental variation of the flow cytometer used, or the speed of sample processing). **b** Autophagic flux in S2 erythroblasts, as interpreted by difference in the MFI of Cyto-ID (an autophagosome dye) between untreated cells and cells treated with an inhibitor of autophagosome formation for 30 min. Each point indicates a foetal liver population, symbols indicate different litters. Mean ± SD are indicated. Analysed by One-way ANOVA followed by Tukey’s test. Relative abundance of (**c**) glycolysis and (**d**) Krebs cycle intermediates, in *Nprl3*^*−/−*^ Ter119+ cells versus littermates, measured by LC-MS. **e **RNA-Seq heatmap showing 11 genes in the ‘Glycolysis’ KEGG pathway that were identified as significantly upregulated in *Nprl3*^*−/−*^ S3 foetal liver compared to *Nprl3*^*+/+*^ S3 foetal liver. Colour intensity indicates the log2 fold change in each sample relative to the mean of wild-type samples. **f** GSH:GSSG ratio, calculated from relative abundances of glutathione and oxidised glutathione. All LC-MS measurements were normalised to the abundance of myristic acid, employed as an internal control. All LC-MS data were compared by two-tailed *t*-test. For all graphed LC-MS data, each point represents one cell population, each derived from separate foetal livers, and the mean ± SD are presented. **g** schematic illustrating the transcriptional and protein-level upregulation of glycolytic enzymes in *Nprl3*^*−/−*^ erythroblasts, and metabolic intermediates with increased abundance. Nprl3 nitrogen permease regulator 3, pS6 phosphorylated S6, Pfkp phosphofructokinase, Psph phosphoserine phosphatase, Gpt2 glutamic-pyruvic transaminase 2, Aldoa Aldoase 1, Phgdh phosphoglycerate dehydrogenase, Psat1 phosphoserine aaminotransferase 1, Tpi1 triosephosphate isomerase 1, Gpi1 glucose-6-phosphate isomerase 1, Pgk1 phosphoglycerate kinase 1, Pkm pyruvate kinase, Shmt2 serine hydroxymethyltransferase−2, MFI median fluorescent intensity, SD standard deviation, Hk hexokinase, Eno enolase, Ldh lactate dehydrogenase. Source data are provided as a Source Data file.

Loss of Nprl3 increased erythroblast phospho-S6 (pS6) signalling in murine E13.5 *Nprl3*^−/−^ foetal liver (Fig. 3a), as in human erythroid cultures. Autophagy is an important contributor to cellular metabolism; it is required for erythropoiesis^32^ and is suppressed by activated mTORC1^15,33^. Therefore, we hypothesised impaired autophagy to be a possible outcome of overactive mTORC1 signalling induced by *Nprl3*^*−/−*^. Consistent with the role of Nprl3 as an mTORC1 inhibitor, autophagic flux was reduced in *Nprl3*^*−/−*^ erythroblasts (S2) from E13.5 foetal liver compared to littermates (Fig. 3b). This metabolic dysregulation may contribute to their inability to efficiently mature to S3 (basophilic erythroblasts) and beyond.

To further explore the effect of *Nprl3*^*−/−*^ on metabolism we analysed a panel of metabolic intermediates in Ter119+ erythroid cells, from E13.5 foetal livers. Loss of Nprl3 did not alter concentrations of tricarboxylic acid cycle metabolites (Fig. 3c), amino acids or nucleotides (with increased levels of ATP relative to other nucleotides as expected in erythroid cells^34^; Supplementary Fig.  3b). However, *Nprl3*^*−/−*^ erythroid cells demonstrated increased abundance of hexose bisphosphate and dihydroxyacetone phosphate (DHAP; Fig. 3d), suggesting relative dysregulation of glycolysis (a major mTORC1 target^35^) at the level of aldolase and triosephosphatase isomerase (TPI). We then performed RNAseq on S1 and S3 wild-type and *Nprl3*^*−/−*^ erythroblasts. RNA-Seq showed that *Aldoa* and *Tpi1* were among seven glycolytic enzymes transcriptionally upregulated in E13.5 *Nprl3*^*−/−*^ erythroblasts, as part of the ‘carbon metabolism’ KEGG pathway that was upregulated at S1 and, to a greater extent, S3 stage (Fig. 3e). Overall, RNA-Seq analysis revealed a greater differential expression profile in *Nprl3*^*−/−*^ erythroblasts versus wild-type at S3 stage (298 upregulated, 365 downregulated) compared to at S1 (140 upregulated, 48 downregulated). (Fig. 3e and Supplementary Fig. 3c, d). This is consistent with the stage-specific loss of S3 erythroblast number we observe in *Nprl3*^*−/−*^ foetal liver. Interestingly, GSEA pathway analysis also suggests upregulation of a type-I interferon response (Supplementary Fig.  3e). In addition, we performed protein-MS on S3 wild-type and *Nprl3*^*−/−*^ erythroblasts. At this stage of differentiation, erythroid cells are predominantly synthesising haemoglobin so that numbers of identified proteins are relatively low. However, we observed significant increases in the protein expression of four central glycolytic enzymes (glucose-6-phosphate isomerase, phosphofructokinase, phosphoglycerate kinase and pyruvate kinase (Supplementary Fig.  3f).

*Nprl3*^−/−^ was also associated with decreased glutathione (GSH) and a decreased GSH: oxidised glutathione (GSSG) ratio (Fig. 3f), suggesting loss of reductive power and altered redox balance. Thus, we observe alterations in mTORC1-glycolysis regulation (summarised in Fig. 3g) and oxidative damage protection. Since enzymopathies affecting either of these processes also cause erythrocytic defects^34^, these data are consistent with the erythroid deficiency in *Nprl3*^*−/−*^ foetal livers and indicate that Nprl3-dependent control of metabolism is required for erythropoiesis.

### The α-globin enhancers increase erythroid *Nprl3* expression

Expression of *Nprl3* RNA increases with erythroid commitment^17^. We hypothesised that the α-globin enhancers drive this upregulation. To investigate this, we used transgenic breeding to eliminate *in cis* interactions between *Nprl3* and all murine α-globin enhancers. This involved the *Nprl3*^*+/−*^ mouse (introduced in Fig. 1), where the transgenic allele contains unperturbed α-globin enhancers^18^, but no *Nprl3* expression is directed from its promoter. The second model harbours heterozygous deletion of all 5 α-globin enhancers (*Nprl3*^*+/AEKO*^; producing little or no α-globin RNA^36^), while *Nprl3* is unperturbed. Please see ‘Methods’ for details of these lines.

**Fig. 4:**
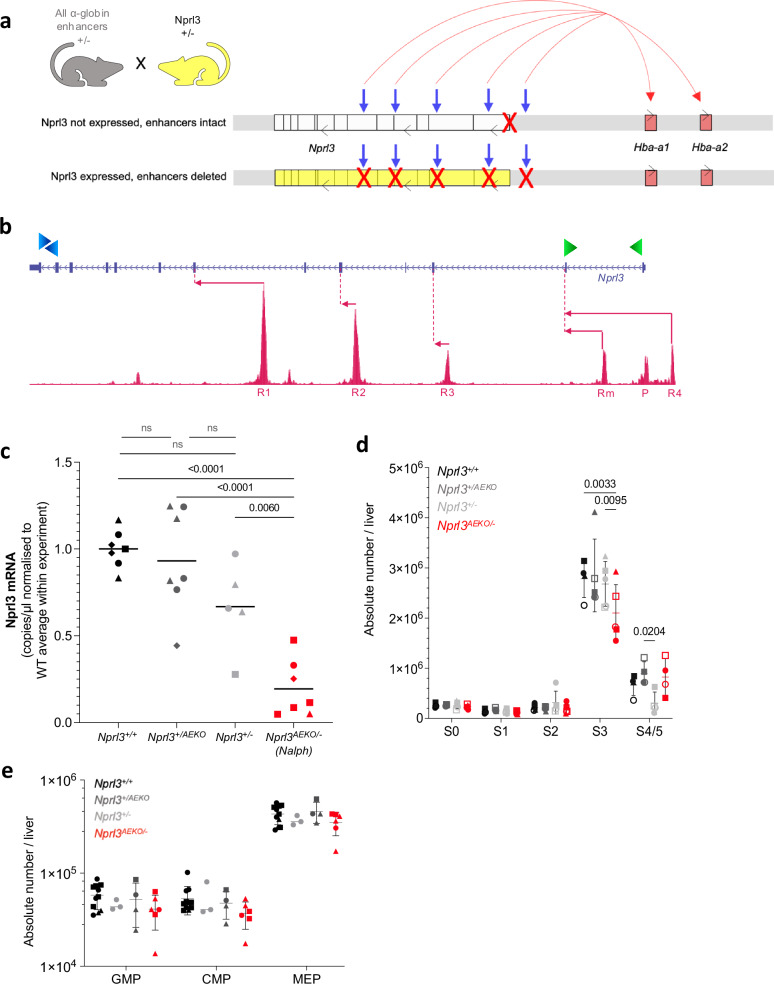
Elimination of interactions between the *Nprl3* promoter and α-globin enhancers. **a** Schematic illustrating the alleles inherited by the *Nalph* genotype, which is heterozygous for both the *Nprl3* promoter deletion, and deletion of all α-globin enhancers. Grey arrows indicate the enhancer locations, and red arrows indicate enhancer induction of sufficient α-globin expression on one allele. **b** Schematic illustrating the digital droplet assay design, with primer locations indicated by green (promoter-derived mRNA) and blue (mRNA + meRNA) arrowheads. Pink arrows indicate transcription starting from enhancers and their first splicing point. **c** Promoter-specific *Nprl3* mRNA expression in E13.5 *Nalph* S3 erythroblasts compared to littermates (*n* = 4 litters, each point represents a foetal liver, each shape represents a litter). Analysed by One-way ANOVA followed by Tukey’s test. **d** Absolute number of erythroid cells in stages S0-S5 of differentiation in E13.5 foetal livers of *Nalph* embryos vs. littermates (*n* = 5 litters, points represent average per litter). Data expressed as median with range. Analysed with by Two-way ANOVA followed by Tukey’s test. **e** Absolute number of GMP, CMP and MEP progenitors per feal liver (*n* = 3 litters, each point represents a foetal liver, each shape represents a litter. Mean ± SD are presented). Nprl3 nitrogen permease regulator 3, Hba alpha-globin, R1/2/3/m/4 multispecies conserved sequence R1/2/3/m/4, P progenitor, AEKO all-enhancer knockout. Source data are provided as a Source Data file.

Cross-breeding heterozygote *Nprl3*^*+/−*^ and *Nprl3*^*+/AEKO*^ mice enabled comparison of *Nprl3* RNA expression in the presence of full *Nprl3* expression (*Nprl3*^*+/+*^), heterozygous *Nprl3* (*Nprl3*^*+/−*^), homozygous *Nprl3* promoters with one allele lacking enhancers (*Nprl3*^*+/AEKO*^), and heterozygous *Nprl3* without possible enhancer influence (*Nprl3*^*AEKO/−*^). Importantly, all *in cis Nprl3*-α-globin enhancer interactions are eliminated in *Nprl3*^*AEKO/−*^ animals. They express one allele with an intact *Nprl3* promoter and coding sequence, but lacking all α-globin enhancers, whereas the other allele lacks the *Nprl3* promoter, but all α-globin enhancers are expressed. These embryos (of novel genotype) were termed ‘*Nalph*’ (see schematic, Fig. 4a).

To determine whether *Nprl3* expression is regulated by the α-globin enhancers in erythroid cells, we measured *Nprl3* mRNA in erythroblasts from all genotypes. *Nprl3* mRNA measurement has previously posed a challenge due to confounding measurement of full-length, spliced, polyadenylated enhancer-derived transcripts (meRNAs)^17^. Please note that meRNAs are not protein-coding and thus have no functional influence. To account for their confounding expression, we designed a high-sensitivity digital droplet PCR assay to measure promoter-specific *Nprl3* transcription (Fig. 4b). This was applied to cDNA from stage S3 sorted erythroblasts from E13.5 foetal livers. This assay was validated using *Nprl3*^*−/−*^ cells, indicating total loss of detectable *Nprl3* mRNA expression with promoter deletion (Supplementary Fig. 4a). The levels of *Nprl3* mRNA measured in *Nalph* erythroid cells were significantly lower than in littermates (Fig. 4c), and in particular, lower than when *Nprl3* lies *in cis* to functional α-globin enhancers in *Nprl3*^*+/−*^. This suggests direct regulation of *Nprl3* by the α-globin enhancers in erythroid cells.

One littermate genotype, *Nprl3*^*+/AEKO*^, accounts for possible indirect effects of the enhancer deletions on *Nprl3* expression, showing no significant difference in *Nprl3* expression compared to *Nprl3*^*+/+*^ or *Nprl3*^*+/−*^. Regulation of *Nprl3* by at least one α-globin enhancer is further supported by high-resolution capture sequencing (MCC-Seq)^18^ data, showing interaction between the *Nprl3* promoter and the R2 enhancer (Supplementary Fig. 4b). Polymerase II (Pol II) ChIP-Seq also indicates reduced Pol II loading at the *Nprl3* promoter when the remaining α-globin enhancers are deleted from a hemizygous α-globin locus in a cellular model (Supplementary Fig. 4c; as described in Blayney et al.^36^; data available, GSE220463). These findings are also consistent with Tri-C data showing that the *Nprl3* promoter is present with the α-globin genes and their enhancers in shared transcriptional hubs^7^.

When present independently, the *Nprl3*^*+/−*^ and *Nprl3*^*+/AEKO*^ genotypes do not affect the life expectancy or general health of adult mice. We gathered haematology readings from the peripheral blood of these mice and their littermates. *Nprl3*^*+/−*^ adults demonstrate no difference in RBC count, size or Hb content compared to WT littermates (Supplementary Fig. 4d). *Nprl3*^*+/AEKO*^ adults demonstrate normal RBC counts, with low MCV and Hb content (Supplementary Fig. 4e). This is reflective of the levels of α-globin expressed: equivalent to WT in *Nprl3*^*+/−*^, and reduced to expected levels in *Nprl3*^*+/AEKO*^ mice (Supplementary Fig. 4f, g). As α-globin enhancers are required for α-globin expression on the allele *in cis*, *Nprl3*^*+/AEKO*^ is equivalent to the expression of 2 of 4 functional α-globin genes (such as in α^0^-thalassaemia carriers and alpha-thalassaemia mice, including Hba^th-J^ (^− −^/^aa^)). Due to the duplication of α-globin, such patients are often asymptomatic and their erythropoiesis is sufficient overall^37^. This viable level of α-globin mRNA is not further reduced by the co-presence of *Nprl3*^*+/−*^ *in cis* (as in *Nalph*), confirming that the *Nalph* phenotype is not driven by alterations to globin expression (Supplementary Fig. 4h). Please note that the reduced α-globin expression associated with *Nprl3*^*+/AEKO*^ does not influence *Nprl3* mRNA expression (dark grey points, Fig. 4c), or erythroid output in the foetal liver (dark grey points, Fig. 4d).

Finally, to assess whether the enhancer-bestowed *Nprl3* transcriptional boost has a significant effect on the erythroid role of Nprl3, we compared the effects of enhanced and unenhanced levels of *Nprl3* RNA on erythroid development via flow cytometry on day E13.5. There was no difference in erythroid development between littermate embryos *Nprl3*^*+/−*^, *Nprl3*^*+/AEKO*^ or WT (Fig. 4d). However, fewer S3 erythroblasts were counted in *Nalph* embryos compared to littermates (Fig. 4d). The stage and direction of this effect is reminiscent of that observed in fully *Nprl3*-deficient (*Nprl3*^*−/−*^) foetal livers. The size of the difference is not as profound as in *Nprl3*^−/−^, which is consistent with decreased but existent *Nprl3* expression in *Nalph* cells, lacking the erythroid transcriptional ‘boost’. Nevertheless, among 12 litters, *Nalph* was absent from 3 and was the least represented genotype overall (Supplementary Fig. 4i), which may suggest a fitness disadvantage. Note that the numbers of haematopoietic progenitors (GMP, CMP and MEP) were not different between genotypes (Fig. 4e), suggesting an erythroid-specific requirement of enhanced *Nprl3* transcription. These results indicate that the α-globin enhancer regulation of *Nprl3* expression in erythroid cells enables optimal red blood cell production.

Overall, our data show that the topologically associated domain containing *Nprl3*, α-globin and their enhancers has a multifunctional role. This locus controls developmental gene expression (α-globin) as well as tuned regulation of erythroid cell metabolism (*Nprl3*), ultimately facilitating erythropoiesis.

## Discussion

Erythroid terminal differentiation requires intense anabolic activity (to synthesise sufficient haemoglobin, which is driven by mTORC1^38^). This is followed by a catabolic phase (to recycle macromolecules and enable the simplification from erythroblast to erythrocyte), which features autophagic activity, also controlled by mTORC1^15^. We show that control of mTORC1 by Nprl3 serves a critical role in erythropoiesis, maintaining appropriate glycolytic metabolism and autophagic flux in erythroblasts. Our work suggests that this developmental process may be particularly vulnerable to Nprl3 deficiency. While our work focusses on definitive erythropoiesis, *Nprl3* is also expressed by primitive erythroid cells, and it is plausible that *Nprl3* also influences metabolic regulation of primitive erythropoiesis^39,40^.

Our results demonstrate that Nprl3 maintains autophagic flux (a critical process in erythroblasts), and regulates mTORC1 responses not only to amino acid availability, but also to iron and EPO. It was recently found that cholesterol levels are also communicated to mTORC1 via GATOR-1 in HEK293T cells^41^. To our knowledge, our results are the first demonstration in ‘non-transformed’ cells for a role of GATOR-1 (and NPRL3) acting as an integrator of environmental signals beyond amino acid availability, and indicate a potential mechanism for the known regulation of mTORC1 by iron in erythroid cells^42^.

Metabolically, Nprl3 deficiency associates with increased glycolytic activity in erythroid cells, as indicated by metabolomic, transcriptomic and proteomic data. Erythroid proliferation and differentiation is supported by glycolysis^43^, and it has been characterised that mTORC1 regulates glycolysis (in mouse embryonic fibroblasts and cancerous cells) via Hif-1 and c-myc^44,45^. Therefore, it is likely that dysregulated glycolysis due to mTORC1 overactivation contributes to the *Nprl3*^*−/−*^ erythroid differentiation defect. Central glycolytic enzymes are upregulated at the mRNA and protein levels, including phosphofructokinase, the ‘gatekeeper’ of glycolysis. The increased DHAP level in *Nprl3*^*−/−*^ erythroid cells is notable, both because DHAP accumulation also occurs in TPI deficiency (linked to severe anaemia)^34^, and because DHAP is known to activate mTORC1^46^. Interestingly, genetic deficiency of TPI causes erythrocyte accumulation of DHAP and severe chronic haemolytic anaemia^47^. It is possible that mTORC1 overactivity driven by loss of Nprl3 may be maintained and exacerbated by increased DHAP, further destabilising metabolic control. Interestingly, glycolysis also supports HSC self-renewal^23^, and HSC commitment to the erythroid lineage^26^. If glycolytic dysregulation also occurs in *Nprl3*^*−/−*^ HSCs, this may contribute to their impairment in the competitive chimaera experiment, and represent a shared metabolic vulnerability of HSCs and erythroid cells. Metabolomic data also suggested that Nprl3 deficiency associates with dysregulation of redox control. Erythrocytes are exposed to oxidative damage and require protective reductive power in the form of glutathione (GSH), which was decreased in *Nprl3*^*−/−*^ erythroblasts. However, as RNA-Seq revealed no differential expression of genes that control antioxidant levels (e.g. *Nrf2, Nqo1* and *Gclc*) between WT and *Nprl3*^*−/−*^, the degree to which redox imbalance contributes to the *Nprl3*^*−/−*^ erythroid phenotype is unclear.

Intriguingly, *Nprl3*^*−/−*^ S3 cells also demonstrate a strong transcriptional type-I interferon response. This could be triggered by DAMPs derived from mitochondrial stress, (e.g. accumulated mitochondrial (mT)DNA/damaged mitochondria, potentially due to impaired autophagy). mtDNA can stimulate innate immune signalling and interferon via cGAS/STING, and mitophagy regulates this activity by clearing dysfunctional mitochondria^48^. The role of interferon signalling in erythroblasts warrants investigation in a separate study, particularly as defects in components of the pathway can influence erythropoiesis^49^.

We have shown that the α-globin enhancers upregulate *Nprl3* transcription, which supports erythropoiesis independently of their critical role supporting α-globin expression. We hypothesise that the presence of the α-globin enhancers enables tuneable *Nprl3* expression during erythropoiesis—helping to maintain sensitive regulation of mTORC1 signalling, particularly in response to environmental conditions and metabolic state. Previous research indicates the importance of full GATOR-1 activity for mTORC1 regulation in conditions of nutritional stress. For example, in conditions of mTORC1 overactivation in neurons (via neuronal-specific *Depdc5*-KO), an exacerbated reduction of cortical amino acid abundance was obsereved^50^.

The phenomenon of enhancer sharing, in which lineage-specific enhancers regulate two different genes in the same locus, has been previously described. Shared enhancers regulate expression of the embryonic, foetal and adult globin genes at both the α-globin^51^ and β-globin loci^52,53^, and at the Hox clusters^54^. While these examples involve paralogous genes that act in the same pathway, enhancer sharing by otherwise unrelated genes has also been described (e.g. *Lnpk* in the HoxD cluster^55^). Interestingly, intronic enhancers within *Formin* critically control *Gremlin* expression in the developing limb, where *Formin* expression is dispensable^56,57^. Both *Formin* and *Gremlin* are involved in kidney development, though to the best of our knowledge, no enhancers have been demonstrated to co-regulate both genes in the kidney. We believe our work demonstrating co-regulation of *Nprl3* and the α-globin genes by the α-globin enhancers constitutes the first direct demonstration of functional cross-talk between distinct cellular pathways (mTORC1 and globin synthesis) relying on shared genomic enhancers, where each component is required for successful completion of a key biological process - in this case, erythropoiesis.

It is thought that, in a common ancestor of all vertebrates, an *Nprl3*-hosted regulatory element (corresponding to mammalian R1) provided a platform to regulate ancestral globin genes^9^. *Nprl3* has remained syntenic with the convergently evolving gnathostome haemoglobin α and agnathan monomeric haemoglobin^58,59^, both of which bind and transport oxygen. Together with the work presented here, this suggests that an ancient *Nprl3*, which regulated metabolism and possessed an enhancer, could have facilitated the tissue-specific expression and upregulation of early oxygen-carrying globins during the evolution of erythropoiesis. Subsequent expansion of regulatory mechanisms, while retaining the proximity of *Nprl3* and α-globin, may have allowed optimisation of red blood cell synthesis over evolutionary time in a species-specific manner.

We would like to note some limitations of this study. The relative contributions of underlying causative mechanisms of *Nprl3*^*−/−*^ erythropoietic failure at S3 are not defined. Plausible, and possibly linked, mechanisms include: autophagic inhibition, which constricts macromolecular availability for biosynthesis (recycling) and prevents maintenance of cellular health (defective clearance); and unsustainable energetic imbalance represented by the dysregulation of glycolysis. Pharmacological interventions during pregnancy to test the contribution of these processes to the erythropoietic phenotype were inconclusive and potentially confounded due to effects of drugs on both mother and foetus. Ex vivo culturing of *Nprl3*^*−/−*^ foetal liver cells did not recapitulate the in vivo phenotype of impaired differentiation, likely due to supraphysiological nutrient availability needed for in vitro differentiation culture conditions. This lack of phenotype meant that rescue experiments (for example, testing the effects of suppressing glycolysis) could not be performed. For some experiments on *Nalph*, sample numbers were limited by breeding constraints and limited material available per foetal liver, per pregnancy. Similarly, it would have been informative to describe true promoter-derived *Nprl3* expression among all haematopoietic lineages using ddPCR, but we were limited by the availability of non-erythroid cellular material per foetal liver. In our metabolomic study, the number of metabolites detected is relatively modest. This is in part because mature erythroblasts have an unusual metabolic profile, in the process of refining their proteome and cellular processes. Deeper study of metabolic pathway dysfunction in Nprl3 deficiency would require metabolic tracer assays. However, RNA-Seq and proteomic data provide pertinent parallel ‘omics’, and together the datasets indicate a general upregulation of glycolytic enzymes. Another limitation is the lack of an available orthogonal method of separating *Nprl3* from the α-globin enhancers. The complexity of having two essential genes and their regulatory elements interspersed at one locus made this experiment particularly demanding. *Nalph*, and its internal littermate controls, result from years of breeding and genetic manipulation^9,20,38^, and cannot currently be recapitulated by in vitro methods. Finally, the presented human erythroid flow cytometry does not feature erythroblast-stage specific analysis, as surface markers of human erythroid maturation change along a continuum, meaning that definition of distinct populations is challenging.

Overall, we show that Nprl3 is functionally important for metabolic control and development of erythroid cells, and that *Nprl3* expression is transcriptionally supported by the α-globin enhancers. We propose that the deep conservation of α-globin and *Nprl3* has been maintained for this locus to control metabolism during erythropoiesis, as well as α-globin production.

## Methods

Animal procedures were performed under the authority of UK Home Office project and personal licences, in accordance with the Animals (Scientific Procedures) Act of 1998, and all studies were approved by the University of Oxford ethical review committee. Blood cones were collected from NHS Blood and Transplant, donors provided generic consent for research use as part of the donation process, and are not identifiable. These samples were used with ethical approval from the Oxfordshire Research Ethics Committee (MREC 03/8/097).

### Mice

The constitutive *Nprl3*-promoter-KO mouse model (*Nprl3*^*−/−*^; previously generated in the WIMM^18^) was rederived from cryopreserved sperm. The line was backcrossed onto a CB57BL/6 J background by breeding heterozygotes with external WT CB57BL/6 J mice (as monitored by the Transnetyx miniMUGA panel to >95% CB57BL/6 J). The commercially available line, Rosa26^tdTomato^, was provided by the Mead group (WIMM). The background of these mice is C57BL6/J. The all-enhancer-KO mouse was derived via the R2-only mouse line (generated by the Higgs group, WIMM^36,60^), by Ben Davies at the Wellcome Centre for Human Genetics (now Crick Institute, London). In brief, R2-only oocytes were microinjected with the following guide RNAs complexed with Cas9: R2 deletion right, GCCGTGACACTTCATGCTCA, and R2 deletion left, TACCTCCAAGGTTTTGCTC. These oocytes were reimplanted into female mice, whose consequent pups were screened by PCR to identify heterozygotes. This colony was crossed to C57BL6/J for two generations. WT C57BL/6JOlaHsd mice used for breeding and backcrossing were ordered from Envigo Ltd. Mice were housed in a 12-h light/dark cycle, in individually ventilated cages. A standard diet (SDS Dietex Services; Cat. No., 801161) was available *ad libitum*. Humidity was maintained between 45–65%, and temperature at 20–24 °C. Mice were euthanised in increasing concentrations of CO_2_, followed by confirmation through cervical dislocation. Timed matings were established for all foetal liver experiments. Mice were used between the ages of 6–18 weeks. Breeding pairs or trios were set up between 15:00 and 17:00, and plug checks were performed the next morning by 07:30. Observance of a vaginal plug marked embryonic day of development (E)0.5. After day E10.5, female mice which had displayed a plug could be visually checked to confirm pregnancy, before schedule 1 culling of the female and embryos on day E13.5. Foetal livers were collected by individual sterile dissection of embryos in cold PBS (0.5% BSA). Embryos used in the in-house transgenic cross model of *Nprl3*^*−/−*^ and all-enhancers^+/−^ carried the following genotypes: *Nprl3*^*+/+*^, *Nprl3*^+/−^, *Nprl3*^*+/AEKO*^ and *Nprl3*^*AEKO/−*^ (Nalph), where ‘+’ indicates the WT Nprl3 allele, ‘−’ indicates the Npr3 promoter KO, and ‘^*AEKO*^*’* indicates the all-enhancer KO. The genotype of each parent varied between experiments.

No sex-based analyses were performed with these mice, as we had no reason to believe sex contributes to phenotype, rather that broad phenotypes exist regardless of sex. Data are generally reported from male and female mice according to litter distribution (with no obvious phenotypic differences observed), with the exception of bone marrow chimaeras (where it is recommended to match donor and recipient to avoid immune reaction). Females used throughout chimaera studies.

### Foetal liver/bone marrow chimaeras

Female Rosa26-tdTomato mice (C57BL/6 J; 12–18 weeks old) were lethally irradiated with 2 × 4.5 Gys, delivered 4 h apart. Bulk foetal liver cells (WT or *Nprl3*^*−/−*^; C57BL/6 J) and Rosa26-tdTomato competitor bone marrow cells were intravenously injected into the recipient tail 2–4 h after irradiation. R26-dTomato recipients were used to ensure that if complete lethal irradiation was not achieved, any residual progenitors could be grouped as ‘bone marrow competitors’. A total of 4.3 × 10^5^ foetal liver cells and 8.6 × 10^5^ tdTomato competitor bone marrow cells were injected per recipient (calculated for a 1:2 ratio, limited by the number of available *Nprl3*^*−/−*^ FL cells). Recipients were administered with (0.16 mg/mL Enrofloxacin (Baytril), Bayer Corporation) in drinking water for 4 weeks post transplantation to prevent bacterial infection. Reconstitution of peripheral blood was assessed by flow cytometry, using 100 μl blood from the recipient tail vein at weeks 4, 8, 12 and 16.

### Flow cytometry for surface antigens

Tissues (bone marrow and foetal liver) were passed through 70- or 40-µm filters to create a single cell suspension. Cell suspensions were normalised for cell number, transferred to a 96-well round bottom plate, centrifuged at 1200 rpm for 5 min, and washed with 200 μl of PBS (2% FBS). Cells were incubated with anti-CD16/32 (FC receptor block) at 1:100 and LIVE/DEAD Fixable (1:400–1:800; Cat. No., 15519340; Invitrogen) in 20 μl PBS for 10 min. Fluorophore-conjugated antibodies were added at final dilutions of 1:50–1:400 and incubated for 15 min at 4 °C in the dark. Cells were washed in PBS (2% FBS) and analysed using an Invitrogen Attune or BD LSR Fortessa flow cytometer. Hoescht 33258 DNA stain (Invitrogen; Cat. No., H3569) was added to human cell populations immediately prior to analysis cells in PBS (2% FBS) for viability assessment (human cells were not stained with LIVE/DEAD Fixable). See Table 1 for antibody details.

**Table 1:** Flow cytometry antibodies used in this study, grouped according to experimental panel

Panel	Marker	Fluorophore	Manufacturer	Cat. No.
Foetal liver	Ter119	PE	BioLegend	116207
Erythroid	CD71	FITC	BD Pharmingen	561936
	NK1.1	APC-Cy7	BioLegend	108723
	CD90.2	APC-Cy7	BioLegend	105327
	B220	APC-Cy7	BioLegend	103224
	CD11b	APC-Cy7	BioLegend	101226
	Gr1	APC/Fire 750	BioLegend	108455
ps6	c-kit	BV421	BioLegend	105827
	CD71	FITC	BD Pharmingen	561936
	Ter119	PE	BioLegend	116207
	pS6	APC	Cell Signaling Technologies	4851
	B220	APC-Cy7	BioLegend	103224
	NK1.1	APC-Cy7	BioLegend	108723
	CD90.2	APC-Cy7	BioLegend	105327
	Gr1	APC/Fire 750	BioLegend	108455
	CD11b	APC-Cy7	BioLegend	101226
Progenitors	CD48	PB	BioLegend	103417
	CD41	BV605	BioLegend	133921
	CD34	FITC	BD Pharmingen	560238
	CD16/32	PercyP Cy5.5	BioLegend	101323
	c-kit	Biotinylated	BioLegend	105803
	Streptavidin	PE	BioLegend	405203
	Sca-1	PE-Cy7	BioLegend	108113
	CD150	APC	BioLegend	115909
	B220	APC-Cy7	BioLegend	103224
	NK1.1	APC-Cy7	BioLegend	108723
	CD90.2	APC-Cy7	BioLegend	105327
	Gr1	APC/Fire 750	BioLegend	108455
	CD11b	APC-Cy7	BioLegend	101226
	Ter119	APC-Cy7	BioLegend	116223
Myeloid	CD11b	BV421	BioLegend	101235
	Ter119	BV510	BioLegend	116237
	Ly6C	BV605	BioLegend	128035
	F4/80	AF488	BioLegend	123119
	CD71	PE	BioLegend	113807
	c-kit	PE-Cy7	BioLegend	105813
	Ly6G	PerCPCy5.5	BioLegend	127615
	CD115	APC	BioLegend	135509
	B220	APC-Cy7	BioLegend	103224
	NK1.1	APC-Cy7	BioLegend	108723
	CD90.2	APC-Cy7	BioLegend	105327
Autophagy	c-kit	BV421	BioLegend	105827
	Cyto-ID	FITC	Enzo	
	Ter119	PE	BioLegend	116207
	Sca-1	PE-Cy7	BioLegend	108113
	CD71	APC	BioLegend	113819
	B220	APC-Cy7	BioLegend	103224
	NK1.1	APC-Cy7	BioLegend	108723
	CD90.2	APC-Cy7	BioLegend	105327
	Gr1	APC/Fire 750	BioLegend	108455
	CD11b	APCCy7	BioLegend	101226
Human				
pS6/4E-BP1	Hoescht 333258	(VL-1)		
	p4E-BP1	PE	Cell Signaling Technologies	7547
	pS6	APC	Cell Signaling Technologies	4851
Foetal liver—bone marrow chimaera				
Progenitors	Ter119	BV421	BioLegend	116233
	CD16/32	BV510	BioLegend	101333
	CD41	BV605	BioLegend	133921
	CD45.2 biotin	n/a	BioLegend	109803
	strepdavidin	BUV395	BD Biosciences	564176
	Sca-1	BV785	BioLegend	108139
	CD105	FITC	BioLegend	120405
	c-kit	PE-Cy7	BioLegend	105813
	CD150	APC	BioLegend	115909
	CD90.2	APC-Cy7	BioLegend	105327
	B220	APC-Cy7	BioLegend	103224
	Nk1.1	APC-Cy7	BioLegend	108723
	CD11b	APC-Cy7	BioLegend	101226
	Ly6C	APC-Cy7	BioLegend	128017
Erythroid	Ter119	FITC	BioLegend	116205
Recipient bone marrow	CD71	PE-Cy7	BioLegend	113811
	CD44	APC	BioLegend	103011
	CD90.2	APC-Cy7	BioLegend	105327
	CD11b	APC-Cy7	BioLegend	101226
	B220	APC-Cy7	BioLegend	103224
	Gr1	APC/Fire 750	BioLegend	108455

### Flow cytometry for intracellular antigens

After processing as above and staining of surface antigens of interest, cells were fixed for 20 min in 150 μl 4% paraformaldehyde (BioLegend; Cat. No., 420801) at 4 °C in darkness before a 30-min permeabilisation step (eBioscience; Cat. No. 008333). Staining for intracellular markers was performed for 30 min in permeabilisation buffer, cells were then centrifuged at 1800 rpm for 2 min and resuspended in permeabilisation buffer for flow cytometric analysis. Flow cytometry gating schemes are provided in Supplementary Fig. 5.

### Autophagic flux analysis

A total of 3 × 10^5^ cells were suspended in RPMI + 5% FCS and plated (in duplicate per sample) in 250 μl in a 96-well plate triplicate. Chloroquine was added to one of the wells for each sample (50 μM; Enzo Life Sciences; Cat. No., ENZ-51031). The plate was incubated at 37 °C and 5% CO_2_ for 2 h. Cells were then processed using the CYTO-ID Autophagy Detection Kit (Enzo Life Sciences; Cat. No., ENZ-51031), as per the manufacturer’s instructions. However, due to the small scale of this assay, only 125 μl diluted Cyto-ID dye was used per sample. Cells were then washed in the provided assay buffer, and surface antigen staining was performed as described above. Cells were immediately analysed by flow cytometry.

### Single-cell sorting of HSPCs

A Sony MA900 Cell Sorter to deposit CFU-Es into single wells of round-bottomed 96-well plates containing pre-warmed HSPC media (see below).

### Isolation of primary CD34^+^ cells

Blood leukocyte cones were donated via the NHS Blood and Transplant Centre (Oxford). Each cone was diluted 1:1 with PBS and layered onto Histopaque-1077 Hybri-Max (Sigma-Aldrich). The suspension was centrifuged at 1800 rpm for 25 min at room temperature, with soft acceleration and deceleration. The interphase PBMC layer was collected with a pasteur pipette and washed twice with PBS. Cells were resuspended in MACS buffer (PBS, 0.5% BSA, 2 mM EDTA) for CD34^+^ magnetic selection using CD34 human MicroBeads (Miltenyi Biotec; Cat. No., 130-046-702; 200 μl beads per cone, incubated at 4 °C on a roller for 30 min), a MidiMACS magnet (Miltenyi Biotec; Cat. No., 130-042-302) and LS columns (Miltenyi Biotec; Cat. No., 130-042-401). Cells were plated immediately into pre-warmed antibiotic-free HSPC media (StemSpan SFEM II; Stemcell Technologies; cat. no., 09605_C) supplemented with SCF (100 ng/ml; Peprotech; Cat. No., 300-07), TPO (100 ng/ml; Peprotech; Cat. No., 300-18) and Flt3 (100 ng/ml; Peprotech; Cat. No., 300-19) at 0.25 × 10^6^ cells/ml.

### In vitro human erythroid differentiation culture from CD34+ cells

This culture system has been previously established^27,28^. On day 0, HSPCs were seeded at 0.25 × 10^6^ cells/ml in phase 1 media (components and their concentrations are listed in Table 2). Cells were counted every 24–48 h from Day 3 using a NucleoCounter NC-3000 (Chemometec). Precisely, cell counts were performed on days 0 (post CD34+ isolation), 3, 4, 5, 7, 9 and 10. Until day 4, cell concentration was maintained at 0.25 × 10^5^ cells/ml. Until day 11, cell concentration was maintained at 5 × 10^5^ cells/ml after each cell count. On day 7, cells were resuspended in phase 2 media. On day 11, cells were resuspended in phase 3 media and maintained at 1 × 10^6^ cells/ml for the remainder of the culture. The NucleoCounter NC-3000 does not detect enucleated cells as live cells, therefore, as cell counts were not continued once from day 10. Enucleation rates are ~20% by day 11, and ~50% by day 14).

**Table 2:** In vitro liquid culture erythroid differentiation media constituents and their working concentrations

Component	Manufacturer	Catalogue No.	Final concentration	
Iscove’s modified Dulbecco’s media (gluta) GlutaMAX	Gibco	31980030	-	1,2,3
Inactivated group AB Plasma	Dept. of Haematology, Oxford University Hospitals Trust	-	3%	1,2,3
FBS	Sigma-Aldrich		2%	1,2,3
Human holo-transferrin	Sigma-Aldrich	T0665	200 μg/ml/500 μg/ml	1,2/ 3
Recombinant human insulin	Sigma-Aldrich	I9278	10 μg/ml	1,2,3
Heparin sodium	Sigma-Aldrich	H3149	3U/ml	1,2,3
Recombinant erythropoietin-a	Bio-Rad	OBT1538	3U/ml	1,2,3
SCF	PeproTech	300-07	10 ng/ml	1,2
Penicillin/Streptomycin	Thermo Fisher Scientific	15140122	100U/ml	1,2,3
Interleukin-3	PeproTech	200-03	1 ng/ml	1

To measure cell growth in terms of fold change in cell concentration over time, the concentration was normalised after every count from day 4 by. Day 7 represents replacement with phase 2 media. Each cell count was compared as a fold change to the preceding normalised concentration. As outlined above, for concentration normalisations, cell concentration was returned to the appropriate concentration for that media phase (0.25 × 10^6^ until day 4, 0.5 × 10^6^ until day 11 and 1 × 10^6^ thereafter).

For culture of singly-sorted HSPCs, cells were maintained in 150 μl phase 1 media in round-bottomed 96-well plates, within plastic boxes containing a reservoir of sterile water to avoid evaporative loses. Cells were not disturbed until day 7, at which point viable colonies were taken forward into phase 2, and media was changed as normal on day 11. The absolute number of enucleated cells was measured on day 11 using Hoescht 33342 (Invitrogen; Cat. No., H3570; 0.5 μl 2.5 mg/ml Hoescht 33342 was added per well in 100 μl IMDM, and incubated at 37 °C for 25 min) for immediate analysis by flow cytometry (Hoescht 33342 not washed out).

### Ribonucleoprotein (RNP) editing

A total of 36–48 h post magnetic isolation, primary human CD34+ cells underwent RNP editing. Cas9 nuclease V3 (IDT; Cat. No., 1081058) and sgRNAs (custom made by Synthego) were complexed at a 1:2.5 molarity ratio (Table 3). This was performed in PCR strip tubes with a 10-min incubation at 18 °C, then cells were kept on ice. Cells were washed in 1 ml PBS in a V-bottom cryovial by centrifugation at 300 × *g* for 5 min, to aid complete removal of PBS before resuspension in P3 Nucleofection Solution (room temperature; P3 Primary Cell 4D-NucleofectorTM X Kit S; Lonza; Cat. No., V4XP-3032). A total of 20 μl solution was used to resuspend 80,000 cells, and these cells were transferred to a cuvette (P3 Primary Cell 4D-NucleofectorTM X Kit S; Lonza) for nucleofection in a 4D-Nucleofector (Lonza) using the DZ100 programme. Cells were rested for 5 min before transferring into 100 μl pre-warmed antibiotic-free HSPC media (supplemented as above). Edited cells were cultured for 24–48 h at 37 °C in 5% CO_2_ before differentiation was initiated.

**Table 3:** Example RNP complexing working conditions

sgRNA mass (μg)	sgRNA volume (μl)	Cas9 mass (μg)	Cas9 working conc. (μg/ml)	Cas9 volume (μl)
1.6	1.6	3	3	1
3.2	3.2	6	6	1

sgRNA sequences: Nprl3 sgRNA 1: 5′-GCAACCAAGUCUGAAAUGUG-3′ Nprl3 sgRNA 3: 5′-UUGAUAAUGUGCGAUUUGUU-3′ NC sgRNA: non-editing sgRNA provided by Synthego (sequence not defined) not corresponding to any genomic region.

### Digital droplet PCR (ddPCR)

#### RNA extraction

1–2 × 10^5^ sorted S3-stage foetal liver erythroblasts were centrifuged at 300 × g for 5 min, the supernatant was removed and the cells were lysed in RLT+ buffer. The resulting lysate was used for RNA extraction using the RNA-easy Plus Mini Kit (Qiagen; Cat. No., 74134) as per the manufacturer’s instructions.

#### cDNA synthesis

Any contaminating DNA was removed from the RNA extract using the high efficiency TURBO DNA-free Kit (ThermoFisher Scientific; Cat. No., AM1907), as per the manufacturer’s instructions. Using 2 μl RNA, cDNA was synthesised using the SuperScriptTM III First-Strand Synthesis System (Thermofisher Scientific; Cat. No. 18080051).

#### ddPCR reaction

The ddPCR reaction mix was prepared using 6 μl cDNA, ddPCR Supermix for Probes no dUTP (2X; Bio-Rad; Cat. No., 1863024), and contained final primer concentrations of 250 nM, and final probe concentrations of 125 nM. The following primer and probe sequences were used, all custom-made by Integrated DNA Technologies.

Primers and probe for *Nprl3* exon 1–2:

Exon1—2-Forward, 5′-CCATCAGCGTGATCCTGGTGAGCT-3′

Exon1—2-Reverse, 5′-CTGGTCATCAGCATGTTCGCCAGTG-3′

Exon1—2-probe: 5′-ACGCGGGGTGCTCCTGGCTCCTCTGGAA-3′ with 5′ HEX dye (PrimeTime®) and 3′ blackhole quencher (BHQ®-1).

Primers and probe for *Nprl3* exon 12–13:

Exon12—13-Forward, 5′-ACTCACCACTGAACAAGAGGATGACAG-3′

Exon12—13-Reverse, 5′-GCCGAGTGTTCTCATTGTACATGATCTC-3′

Exon1—2 probe: 5′-TGGTGGCGGCCACGGAAGTAGTGAAGGA-3′ with 5′ FAM dye (PrimeTime®) and 3′ blackhole quencher (BHQ®-1).

Primers for *Gapdh* housekeeping control:

Gapdh_ddPCR-Forward, 5′-AGGTCGGTGTGAACGGATTTG-3′

Gapdh_ddPCR-Reverse, 5′-TGTAGACCATGTAGTTGAGGTCA-3′

Probe for Gapdh housekeeping control: 5′-ATTGGGCGCCTGGTCACCAGGGCT-3′ with 5′ FAM dye (PrimeTime®) and 3′ blackhole quencher (BHQ®-1).

A foil plate lid was then applied at 180 °C for 3 s. Droplets were generated using a BioRad Droplet Generator and Bio Rad ‘Oil for Probes’. The droplets were then exposed to the following thermocycling conditions to complete the ddPCR reaction:

1. Initial denaturation: 10 min 95 °C
2. Denaturation: 30 s at 94 °C
3. Annealing/Extension: 1 min at 60 °C (AEKD WT and KO) (Steps 2 and repeated for 40 cycles)
4. Enzyme inactivation: 10 min at 98 °C

(Ramp rate of 2 °C/sec applied at all stages). Product was measured using a QX200 Droplet Reader (Bio-Rad).

#### Analysis

The presented mRNA/eRNA readings (copies/µl) are generated by the QX200 Droplet Reader, using an internal BioRad internal algorithm. To precisely normalise for input concentration, copies/µl readings were first normalised to that of a reference gene, Gapdh. Next, they were normalised to the average readings for the control genotype (*Nprl3*^*+/+*^ *AEKO*^*+/+*^) in each litter.

### RNA-seq

S1/S3 erythroblasts were FACS sorted from E13.5 foetal liver, according to Ter119 and CD71 expression, using a BD FACS Aria Fusion cytometer. 10–30,000 erythroblasts were sorted directly into lysis solution from the RNAqueous™-Micro Total RNA Isolation Kit (Thermo Fisher Scientific; cat. no. AM1931). RNA extraction was then performed with this kit, according to the manufacturer’s instructions. RNA quality was assessed using an Agilent high-sensitivity RNA ScreenTape (Agilent, 5067-5579) and a 4200 TapeStation system (Agilent, G2991BA).

The RNA was amplified by Novogene using an in-house ‘SMARTer amplification’ pipeline, prior to mRNA library preparation (poly A enrichment). Novogene performed bulk mRNA sequencing to a depth of 30 million reads using the Illumina NovaSeq X Plus Series, and sequencing strategy, PE150.

RNA-seq reads were mapped to mm39 using STAR v2.7.9 with default parameters. A read count matrix was assembled using featureCounts v1.6.4 and the Ensembl gene annotation v104. Differential expression analysis was conducted using DESeq2 v 1.38.3 and R v 4.2.1 and an adjusted *P*-value threshold of 0.05. Initial analysis was conducted blinded to sample genotype. Sample sex was assigned based on the number of reads mapping to genes located on chromosome Y. Gene ontology analysis was conducted using DAVID, comparing genes with padj <0.05, mean expression >10 and log2 fold change >0.6 (upregulated) or <−0.6 (downregulated) with a background gene list of all genes with mean expression >10. Analysis scripts can be found at https://github.com/beagrie-lab/preston-rna-seq.

### Metabolomics

Ter119+ cells were extracted using Anti-Ter119 MicroBeads (Milenyi Biotec; Cat. No., 130-049-901), and LS columns (Milenyi Biotec), according to the manufacturer’s instructions. All reagents were kept on ice. Ter119+ cells were washed in PBS, pelleted, and immediately exposed to 80% methanol containing 2 µM d27 myristic acid and incubated for 3 min on ice. Samples were centrifuged at 17,000 × *g* for 15 min. The supernatant was transferred to a new Eppendorf and stored at −80 °C until metabolite analysis.

Mass Spectrometry measurements were performed using Dionex UltiMate 3000 LC System (Thermo Scientific) coupled to a Q Exactive Orbitrap mass spectrometer (Thermo Scientific) operated in negative mode. Ten microliter sample was injected onto a Poroshell 120 HILIC-Z PEEK Column (Agilent InfinityLab). A linear gradient was carried out starting with 90% solvent A (acetonitrile) and 10% solvent B (10 mM Na-acetate in mqH2O, pH 9.3). From 2 to 12 min the gradient changed to 60% B. The gradient was kept on 60% B for 3 min and followed by a decrease to 10% B. The chromatography was stopped at 25 min. The flow was kept constant at 0.25 ml/min. The column temperature was kept constant at 25 °C. The mass spectrometer operated in full scan (range [70.0000–1050.0000]) and negative mode using a spray voltage of 2.8 kV, capillary temperature of 320 °C, sheath gas at 45, auxiliary gas at 10. AGC target was set at 3.0E + 006 using a resolution of 70000. Data collection was performed using the Xcalibur software (Thermo Scientific). The data analyses were performed by integrating the peak areas (El-Maven–Polly–Elucidata).

Absolute abundance measurements were normalised to the internal standard (myristic acid or D7-glucose) to generate relative abundance.

### Proteomics

Cell lysis and protein digestion:

2 × 10^5^ S3 erythroblasts were sorted into 1% RPMI-FBS, washed twice with HBSS, and cell pellets were snap frozen. Cell pellets were lysed in 100 µl lysis buffer (5% sodium dodecyl sulphate, 50 mM triethylammonium bicarbonate (pH 8.5) and 10 mM tris(2-carboxyethyl)phosphine-hydrochloride) and lysates were shaken at room temperature at 1000 rpm for 5 min before being incubated at 95 °C at 500 rpm for 5 min. Samples were then sonicated using a BioRuptor (15 cycles: 30 s on and 30 s off) and alkylated with 20 mM iodoacetamide for 1 h at 22 °C in the dark. Protein concentration was determined using the EZQ protein quantitation kit (Thermo Fisher Scientific) and protein cleanup and digestion were performed using S-TRAP micro columns (Protifi). Proteins were digested with trypsin at 1:10 ratio (enzyme:protein) for 2 h at 47 °C. Peptides were eluted from S-TRAP columns in a 3 step process. Firstly, peptides were eluted using 50 mM ammonium bicarbonate, followed by 0.2% aqueous formic acid and lastly 50% aqueous acetonitrile containing 0.2% formic acid. Peptides were dried by speedvac before resuspending in 1% formic acid. Peptide quantity was measured using the CBQCA kit (Thermo Fisher Scientific).

LC-MS/MS analysis:

Peptides were analysed by data-independent acquisition (DIA) on a Thermo Orbitrap Astral mass spectrometer coupled to a Vanquish liquid chromatography system. For each sample, 200 ng digested peptide was loaded onto the LC system and samples were analysed using the SPD 60 method. Mass spectrometry raw files were searched using Spectronaut (Biognosys) version 17. Raw mass spec files were searched against a mouse database (Swissprot Trembl November 2023) with the following parameters: directDIA, false discovery rate set to 1%, protein N-terminal acetylation and methionine oxidation were set as variable modifications and carbamidomethylation of cysteine residues was selected as a fixed modification.

Protein intensity values were normalised by dividing the raw intensity value by the sum of all intensity values for the sample. *P*-values were calculated using a two-tailed, unequal variance *t*-test on the log2(normalised intensity values). A significance threshold was set at *p* < 0.05. The heatmap was plotted using normalised intensity values for significantly altered glycolytic proteins.

### Statistics

Statistics were performed in GraphPad Prism software (Version 9.4.1). Statistical significance was defined to be indicated by *p* < 0.05. Normality checks were performed before parametric testing. *T*-tests were performed when comparing the means of two groups, and paired if samples were matched between groups. ANOVA was used to make multiple comparisons between >2 normally distributed groups. One-way or Two-way ANOVA was performed depending on the number of independent variables (one or two, respectively). For optimal power when multiple comparisons testing, the post-hoc test used depended on the comparisons being made. Tukey’s test was performed when comparing every row (or column) mean with every other row (or column) mean. Šídák’s test was used to provide more power when comparing rows (or columns) within columns (or rows). When including individual repeat datapoints in grouped statistical analysis, a consistent number of repeats for every group is required to perform ANOVA. In the rare situation that single repeat measurements were missing for particular groups, a Prism Mixed-effects model was performed to maintain the power of using replicate data, and to generate comparisons equivalent to those made by ANOVA. For the -squared test, observed and expected numbers were compared with 3 degrees of freedom.

### Reporting summary

Further information on research design is available in the Nature Portfolio Reporting Summary linked to this article.

## Supplementary information

Supplementary Information

Reporting Summary

Transparent Peer Review file

## Source data

Source Data

## Data Availability

Data supporting the findings of this study are available in the article and its Supplementary information. Source data are provided as Source Data file and may be obtained from the corresponding authors upon request. RNA-Seq data generated for this study have been deposited on the Gene Expression Omnibus (GEO) database under accession: GSE273384. ATAC-Seq raw sequence data generated are available under accession: GSE174110. The mass spectrometry proteomics data have been deposited to the ProteomeXchange Consortium via the PRIDE partner repository with the dataset identifier: PXD060557. Source data are provided with this paper.
