# Supplementary material for: Ancient genomic linkage of α-globin and Nprl3 couples metabolism with erythropoiesis
Source: Nat Commun. 2025 Mar 24;16:2749. doi: 10.1038/s41467-025-57683-z (PMC11933693; doi:10.1038/s41467-025-57683-z)
Supplement: Supplementary file 1 — Supplementary Information [file 41467_2025_57683_MOESM1_ESM.pdf]

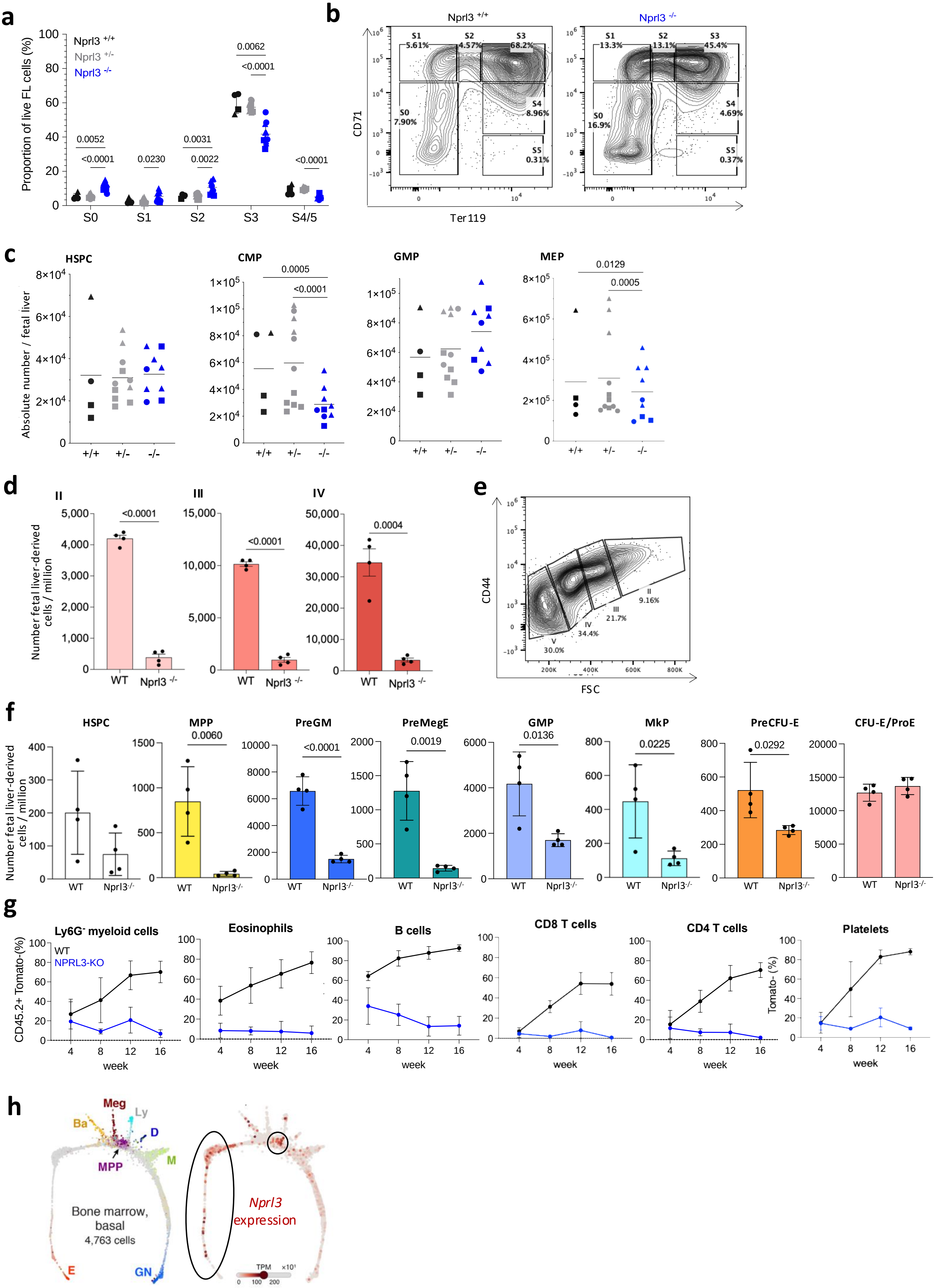
a
Proportion of live FL cells (%)
Nprl3 +/+
Nprl3 +/-
Nprl3 -/-
0.0052
<0.0001
0.0230
0.0031
0.0022
0.0062
<0.0001
<0.0001
S0
S1
S2
S3
S4/5
b
Nprl3 +/+
Nprl3 -/-
S1 5.61%
S2 4.57%
S3 68.2%
S4 8.96%
S0 7.90%
S5 0.31%
S1 13.3%
S2 13.1%
S3 45.4%
S4 4.69%
S0 16.9%
S5 0.37%
CD71
Ter119
c
HSPC
CMP
GMP
MEP
Absolute number / fetal liver
0.0005
<0.0001
0.0129
0.0005
+/+
+/-
-/-
d
II
III
IV
Number fetal liver-derived cells / million
<0.0001
<0.0001
0.0004
WT
Nprl3 -/-
e
CD44
FSC
II 9.16%
III 21.7%
IV 34.4%
V 30.0%
f
HSPC
MPP
PreGM
PreMegE
GMP
MkP
PreCFU-E
CFU-E/ProE
Number fetal liver-derived cells / million
0.0060
<0.0001
0.0019
0.0136
0.0225
0.0292
WT
Nprl3-/-
g
Ly6G- myeloid cells
Eosinophils
B cells
CD8 T cells
CD4 T cells
Platelets
WT
NPRL3-KO
CD45.2+ Tomato-(%)
Tomato- (%)
week
h
Meg
Ly
Ba
D
MPP
M
Bone marrow, basal
4,763 cells
E
GN
Nprl3 expression
TPM

Supplementary Fig. 1. Effects of *Nprl3*$^{-/-}$ in the fetal liver and in competitive chimaeric mice. a) Erythroid stages as a proportion of total live fetal liver cells according to genotype (n = 3 litters). Points indicate individual embryos. Data are expressed as the mean ± SD. Analysed by Two-way ANOVA followed by Tukey's test. b) Example gating of erythroid stages in the fetal liver at E13.5. c) Absolute number per fetal liver of: total c-kit+ cells of lineage- Sca-1+ HSPCs, CD34+ CD16/32+ GMPs, CD34+ CD16/32- CMPs and CD34- CD16/32- MEPs (n = 5 litters). Each point represents an individual embryo, and litter indicated by shape of point. Data are expressed as the mean ± SD, and analysed by Two-way ANOVA (grouped by litter) followed by Tukey's test. d) Absolute number of erythroblasts in stages II, II and IV derived from either WT or *Nprl3*$^{-/-}$ fetal liver cells, per million recipient bone marrow cells. Analysed in recipient bone marrow at week 16 post-transplantation (n = 8 mice per group). Data analysed by two-tailed t-test, and expressed as the mean ± SD. e) Example gating of erythroid stages in the bone marrow. f) Absolute number of fetal liver-derived (CD45.2+ Tomato-) progenitors per million recipient bone marrow cells at week 16 post transplantation (n = 4 mice per group). Data analysed by two-tailed t-test, and expressed as the mean ± SD. g) Temporal contribution of *Nprl3*$^{-/-}$ FL cells to peripheral mature cell types: Ly6G- myeloid cells, eosinophils, B cells, CD4/8 T cells and platelets. h) Single cell RNA-Seq measured from mouse bone marrow-derived cells. The spatial positions of HSCs and erythroid precursors are indicated. Adapted from an online tool published in Tusi *et al.* 2018. https://kleintools.hms.harvard.edu/paper_websites/tusi_et_al/. Source data are provided as a Source Data file.

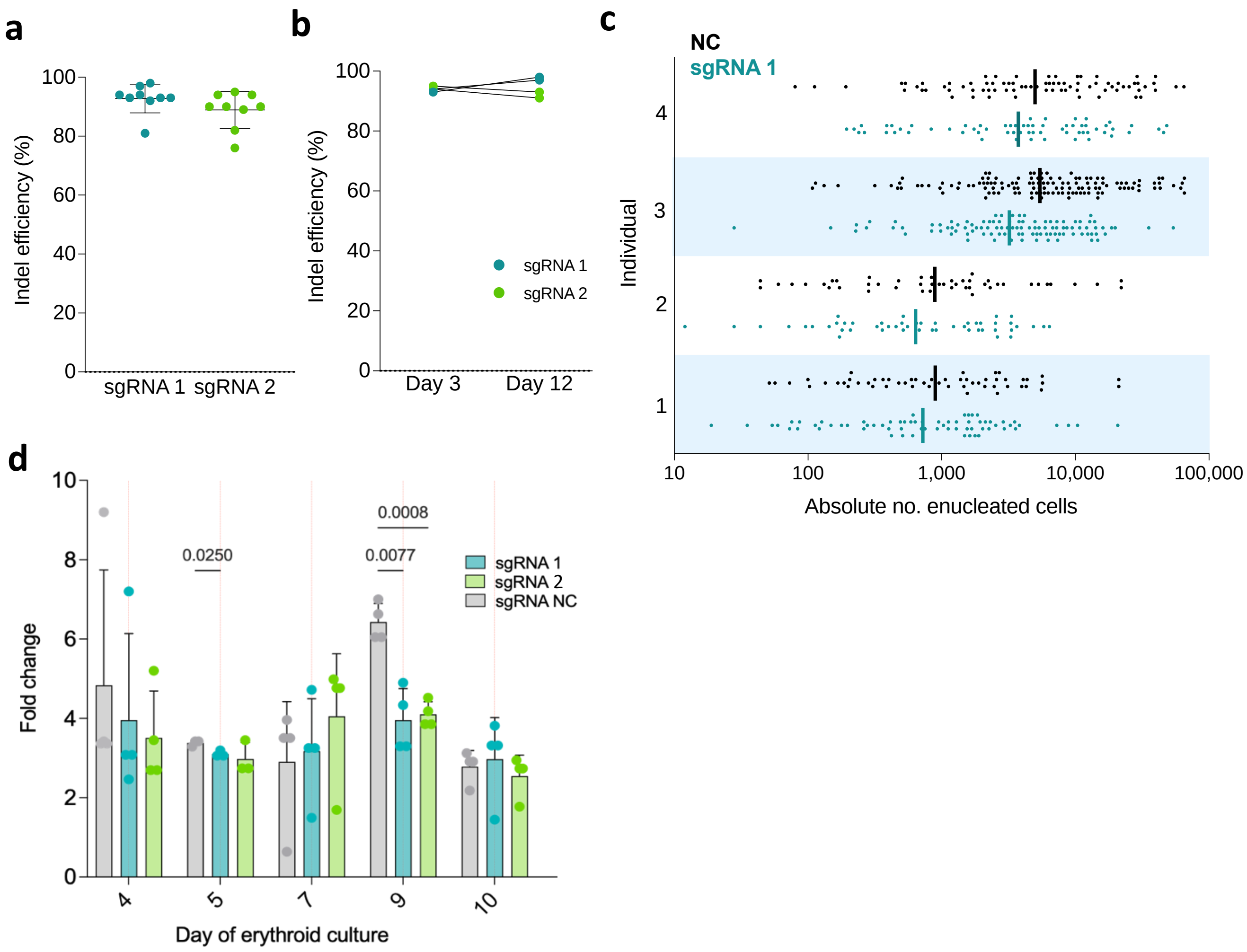

Supplementary Fig. 2. Effects of NPRL3-KO on primary human *in vitro* erythroid differentiation.

a) Editing efficiency (% reads with identical knockout indel) achieved with NPRL3-targeting sgRNAs, sgRNA 1 and sgRNA 2. Each point represents a nucleofected donor population. b) Maintenance of editing efficiency from day 3 to day 12 of erythroid culture. c) Absolute number of enucleated cells formed from a single starting cell (each point represents a single starting cell). Bars indicate the geometric mean. Four individual donors are represented, stacked. d) Fold change in cell concentration throughout erythroid differentiation compared to previous dilution. Cells were counted on days 3, 4, 5, 7, 9 and 10, and concentration was normalised after every count from day 4 by media top up (except for day 7, which represents replacement with phase 2 media). Plotted points indicate the fold change in cell concentration relative to the normalised concentration established after the previous count point. Data represents 3 individual donors, expressed as the mean ± SD, and analysed by Prism Mixed-effects analysis matched by donor individual, followed by Tukey's test. Source data are provided as a Source Data file.

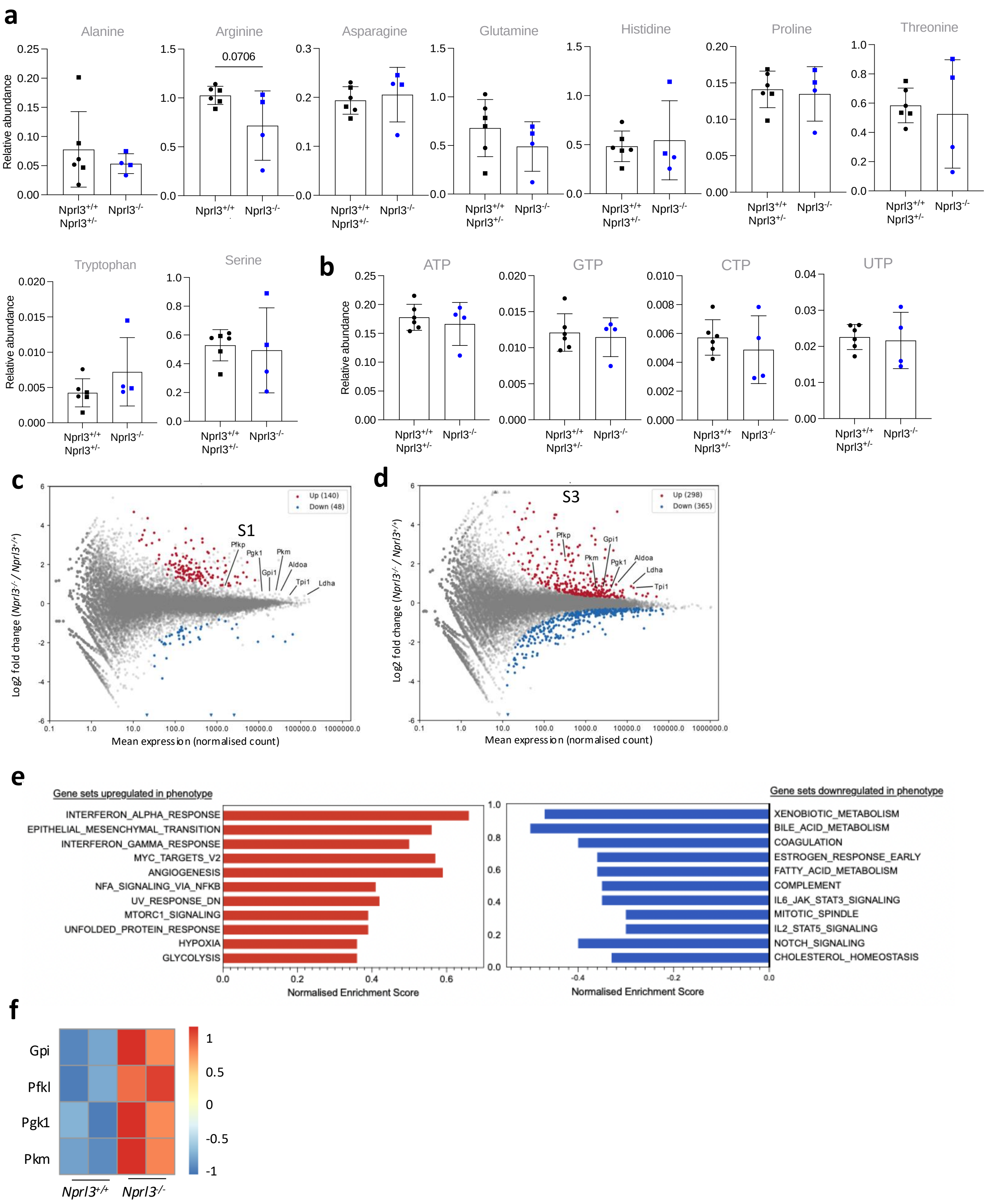

a
Alanine
Arginine
0.0706
Asparagine
Glutamine
Histidine
Proline
Threonine
Tryptophan
Serine
Relative abundance
Nprl3+/+ Nprl3+/-
Nprl3-/-
b
ATP
GTP
CTP
UTP
c
S1
Up (140)
Down (48)
Pfkp
Pgk1
Pkm
Gpi1
Aldoa
Tpi1
Ldha
Log2 fold change (Nprl3-/- / Nprl3+/+)
Mean expression (normalised count)
d
S3
Up (298)
Down (365)
e
Gene sets upregulated in phenotype
INTERFERON_ALPHA_RESPONSE
EPITHELIAL_MESENCHYMAL_TRANSITION
INTERFERON_GAMMA_RESPONSE
MYC_TARGETS_V2
ANGIOGENESIS
NFA_SIGNALING_VIA_NFKB
UV_RESPONSE_DN
MTORC1_SIGNALING
UNFOLDED_PROTEIN_RESPONSE
HYPOXIA
GLYCOLYSIS
Normalised Enrichment Score
Gene sets downregulated in phenotype
XENOBIOTIC_METABOLISM
BILE_ACID_METABOLISM
COAGULATION
ESTROGEN_RESPONSE_EARLY
FATTY_ACID_METABOLISM
COMPLEMENT
IL6_JAK_STAT3_SIGNALING
MITOTIC_SPINDLE
IL2_STAT5_SIGNALING
NOTCH_SIGNALING
CHOLESTEROL_HOMEOSTASIS
f
Gpi
Pfkl
Pgk1
Pkm
Nprl3+/+
Nprl3-/-

Supplementary Fig. 3. Further multi-omic data assessing dysregulations in *Nprl3$^{-/-}$* erythroblasts.

a) Relative abundance of amino acids, measured by LC-MS, normalised to D7-glucose (internal control). b) Relative abundance of nucleotides, measured by LC-MS normalised to myristic acid (internal control). All LC-MS data were compared by two-tailed t-test. Differential expression analysis in S1 and S3. Each point gives the mean expression of a single gene and the log2 fold change in *Nprl3-/-* samples compared to *Nprl3$^{+/+}$* samples. Genes that were not significantly differentially expressed are plotted in grey, significantly upregulated genes in red and significantly downregulated genes in blue. c and d) Differential expression analysis in c) S1 and d) S3. Each point gives the mean expression of a single gene and the log2 fold change in *Nprl3$^{-/-}$* samples compared to *Nprl3$^{+/+}$* samples. Genes that were not significantly differentially expressed are plotted in grey, significantly upregulated genes in red and significantly downregulated genes in blue. e) Top up and downregulated "hallmark" gene sets identified by RNA-Seq GSEA. f) Proteins in the KEGG_glycolysis_glucogenesis gene set identified as significantly upregulated in *Nprl3$^{-/-}$* samples compared to *Nprl3$^{+/+}$* by proteomics, with Gpi manually added. Source data are provided as a Source Data file.

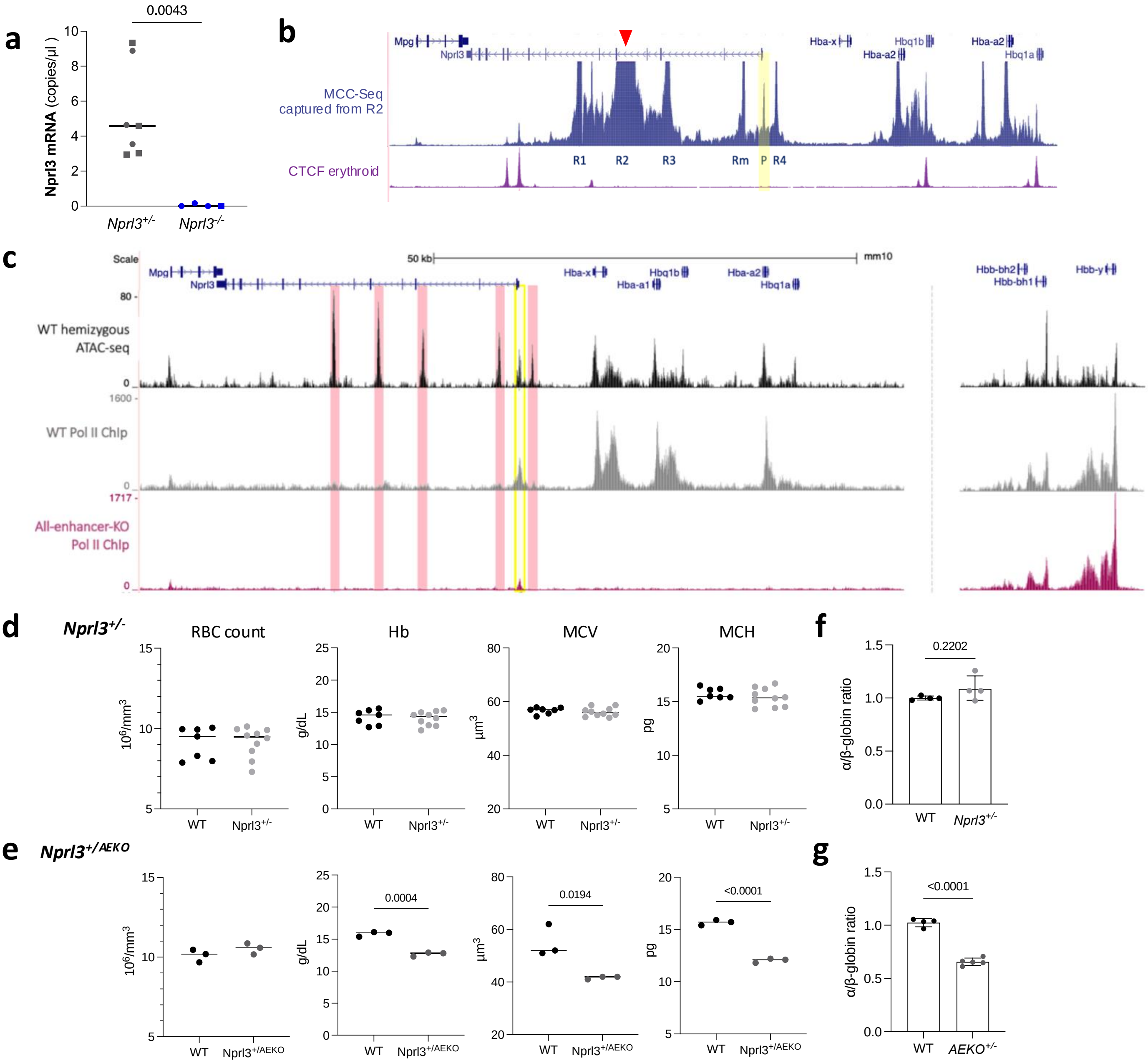

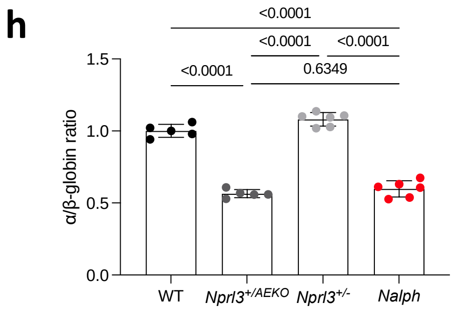

i

Genotype	*Nprl3*+/+	*Nprl3*+/AEKO	*Nprl3*+/-	*Nprl3*AEKO/- (*Nalph*)
Embryo number	36	22	26	17
Chi-squared, p = 0.052				

Supplementary Fig. 4. Further characterisation of the Nprl3-α-globin transcriptional hub.

a) Promoter-specific *Nprl3* mRNA expression in *Nprl3*$^{-/-}$ S3 erythroblasts (a negative control for Fig. 4c) compared to *Nprl3*$^{+/-}$ littermates (n = 2 litters, no WT embryos were present). Analysed by unpaired t-test. Each point represents an embryo, with litter represented by shape. b) MCC-Seq snapshot captured from the R2 enhancer (anchor point indicated by red arrowhead). Yellow panel highlights the interaction of the R2 enhancer with the Nprl3 promoter (P). c) Pol II ChIP-Seq in WT erythroid cells, and following deletion of all α-globin enhancers from cells hemizygous for the locus including nprl3 and alpha (all-enhancer-KO). Enhancers indicated by pink panels, and the *Nprl3* promoter by yellow. d, e) Peripheral blood measurements (RBC count, Haemoglobin (Hb), mean corpuscular volume (MCV) and mean corpuscular haemoglobin (MCH)) of d) *Nprl3*$^{+/-}$, and e) *Nprl3*$^{+/AEKO}$ adults and their littermate WT controls. Analysed by two-tailed t-test. Data expressed as the mean ± SD. f, g, h) α/β-globin mRNA expression ratio measured by RT-qPCR on f) littermate WT and *Nprl3*$^{+/-}$, *g*) littermate WT and *AEKO*$^{+/-}$ Ter119+ cells, and h) littermates from the *Nprl3*$^{+/-}$ x *Nprl3*$^{+/AEKO}$ cross. Analysed by unpaired t-test (f and g) or One-way ANOVA (h). i) Number of embryos of each genotype across 12 litters of *Nprl3*$^{+/-}$ x *Nprl3*$^{+/AEKO}$ animal crosses. Source data are provided as a Source Data file.

a

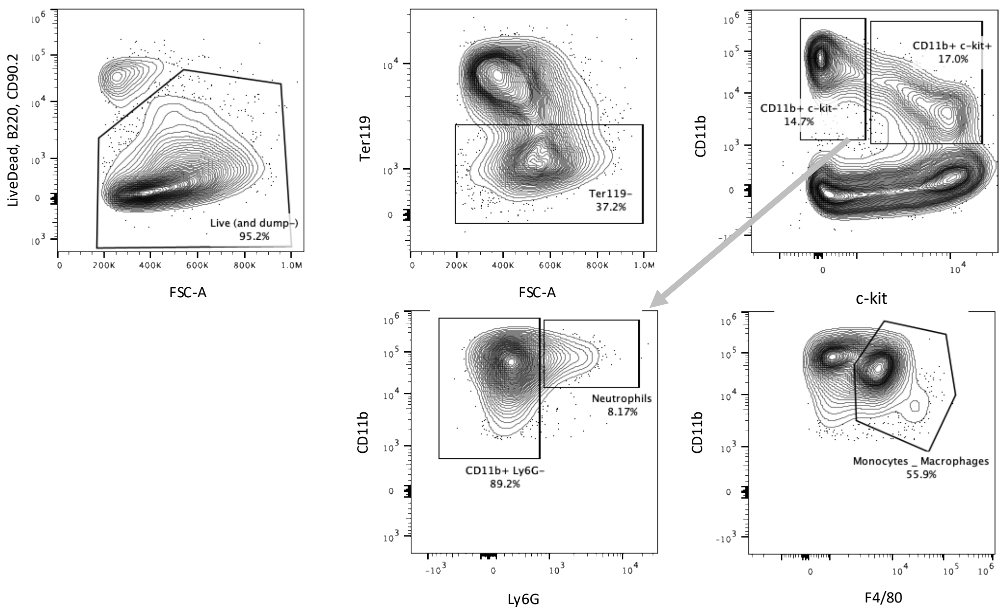
LiveDead, B220, CD90.2
FSC-A
Live (and dump-)
95.2%
Ter119
FSC-A
Ter119-
37.2%
CD11b
c-kit
CD11b+ c-kit+
17.0%
CD11b+ c-kit-
14.7%
CD11b
Ly6G
Neutrophils
8.17%
CD11b+ Ly6G-
89.2%
CD11b
F4/80
Monocytes _ Macrophages
55.9%

b

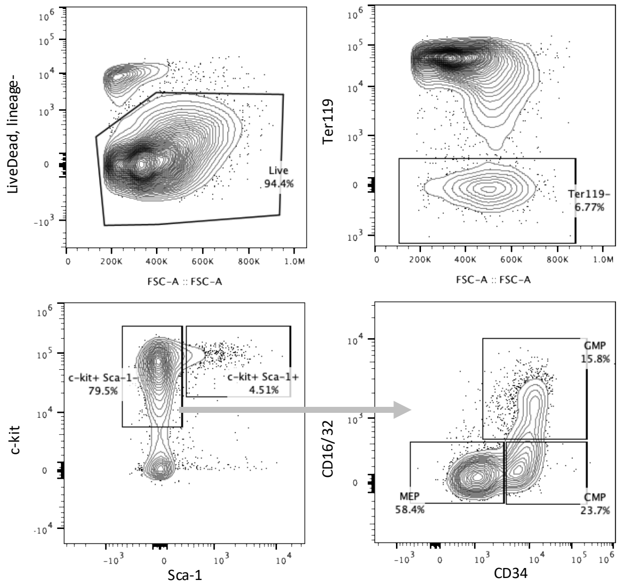
LiveDead, lineage-
FSC-A :: FSC-A
Live
94.4%
Ter119
FSC-A :: FSC-A
Ter119-
6.77%
c-kit
Sca-1
c-kit+ Sca-1-
79.5%
c-kit+ Sca-1+
4.51%
CD16/32
CD34
GMP
15.8%
MEP
58.4%
CMP
23.7%

c

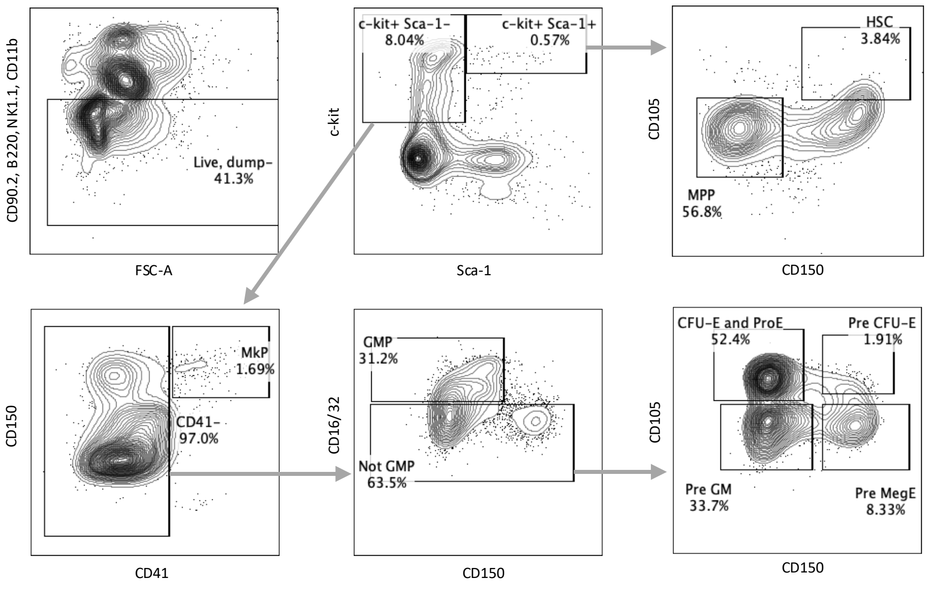
LiveDead
CD90.2, B220, NK1.1, CD11b
FSC-A
Live, dump-
41.3%
c-kit
Sca-1
c-kit+ Sca-1-
8.04%
c-kit+ Sca-1+
0.57%
CD105
CD150
HSC
3.84%
MPP
56.8%
CD150
CD41
MkP
1.69%
CD41-
97.0%
CD16/32
CD150
GMP
31.2%
Not GMP
63.5%
CD105
CD150
CFU-E and ProE
52.4%
Pre CFU-E
1.91%
Pre GM
33.7%
Pre MegE
8.33%

Supplementary Fig. 5. Representative flow cytometry gating schemes.
a) Myeloid cell types (as per Fig. 1f). b) Haematopoietic progenitors in the fetal liver (as per Supplementary Fig. 1c).
c) Haematopoietic progenitors in the bone marrow (as per Supplementary Fig. 1f)
