# Supplementary material for: Ancient genomic linkage of α-globin and Nprl3 couples metabolism with erythropoiesis
Source: Nat Commun. 2025 Mar 24;16:2749. doi: 10.1038/s41467-025-57683-z (PMC11933693; doi:10.1038/s41467-025-57683-z)
Supplement: Supplementary file 2 — Reporting Summary [file 41467_2025_57683_MOESM2_ESM.pdf]

nature portfolio

Corresponding author(s): Alexandra Preston

Last updated by author(s): 13/02/2024

Reporting Summary

Nature Portfolio wishes to improve the reproducibility of the work that we publish. This form provides structure for consistency and transparency in reporting. For further information on Nature Portfolio policies, see our Editorial Policies and the Editorial Policy Checklist.

Statistics

For all statistical analyses, confirm that the following items are present in the figure legend, table legend, main text, or Methods section.

n/a	Confirmed	
☐	☒	The exact sample size (*n*) for each experimental group/condition, given as a discrete number and unit of measurement
☐	☒	A statement on whether measurements were taken from distinct samples or whether the same sample was measured repeatedly
☐	☒	The statistical test(s) used AND whether they are one- or two-sided<br>*Only common tests should be described solely by name; describe more complex techniques in the Methods section.*
☒	☐	A description of all covariates tested
☐	☒	A description of any assumptions or corrections, such as tests of normality and adjustment for multiple comparisons
☐	☒	A full description of the statistical parameters including central tendency (e.g. means) or other basic estimates (e.g. regression coefficient) AND variation (e.g. standard deviation) or associated estimates of uncertainty (e.g. confidence intervals)
☐	☒	For null hypothesis testing, the test statistic (e.g. F, t, r) with confidence intervals, effect sizes, degrees of freedom and P value noted<br>*Give P values as exact values whenever suitable.*
☒	☐	For Bayesian analysis, information on the choice of priors and Markov chain Monte Carlo settings
☒	☐	For hierarchical and complex designs, identification of the appropriate level for tests and full reporting of outcomes
☐	☒	Estimates of effect sizes (e.g. Cohen's *d*, Pearson's *r*), indicating how they were calculated

Our web collection on statistics for biologists contains articles on many of the points above.

Software and code

Policy information about availability of computer code

Data collection	Flow cytometry data collected using either Thermo Fisher Attune of BD LSR Fortessa cytometers. Blood measurements from ABX Pentra 60 haematology analyser. Metabolite measurements collected from Q Exactive Orbitrap or Q Exactive Focus Orbitrap mass spectrometers. ddPCR readings collected using a BioRad QX300 ddPCR Reader. RNA-Seq collected using the Illumina NovaSeq X Plus Series.
Data analysis	Flow cytometry analysis – FlowJo version 10.<br>Graphing and statistics – GraphPad Prism version 9.<br>Knockout efficiency – Synthego ICE CRISPR Analysis Tool.<br>RNA-Seq analysis - DESeq2 version 1.38.3 and R version 4.2.1.<br>Mass spec proteomics - Spectronaut (Biognosys) version 17.

For manuscripts utilizing custom algorithms or software that are central to the research but not yet described in published literature, software must be made available to editors and reviewers. We strongly encourage code deposition in a community repository (e.g. GitHub). See the Nature Portfolio guidelines for submitting code & software for further information.

Data

Policy information about availability of data

All manuscripts must include a data availability statement. This statement should provide the following information, where applicable:

- Accession codes, unique identifiers, or web links for publicly available datasets
- A description of any restrictions on data availability
- For clinical datasets or third party data, please ensure that the statement adheres to our policy

RNA-Seq data has been deposited on the Gene Expression Omnibus (GEO) database under accession: GSE273384 [www.ncbi.nlm.nih.gov/geo/query/acc.cgi?acc=GSE273384].
The mass spectrometry proteomics data have been deposited to the ProteomeXchange Consortium via the PRIDE [https://www.ebi.ac.uk/pride/] partner repository with the dataset identifier: PXD060557 [www.ebi.ac.uk/pride/archive/projects/PXD060557].
All other data supporting the findings of this study are available within the paper and its Supplementary Information. Source data are provided with this paper.

Research involving human participants, their data, or biological material

Policy information about studies with human participants or human data. See also policy information about sex, gender (identity/presentation), and sexual orientation and race, ethnicity and racism.

Reporting on sex and gender	N/A<br><br>Note that the human data in this study derive from donated blood cones (NHSBT) which are never accompanied by any information on the donating individual.
Reporting on race, ethnicity, or other socially relevant groupings	N/A
Population characteristics	N/A
Recruitment	N/A
Ethics oversight	All studies were approved by the University of Oxford ethical review committee. Donors give informed consent for general research use at the point of sample collection.

Note that full information on the approval of the study protocol must also be provided in the manuscript.

Field-specific reporting

Please select the one below that is the best fit for your research. If you are not sure, read the appropriate sections before making your selection.

☒ Life sciences ☐ Behavioural & social sciences ☐ Ecological, evolutionary & environmental sciences

For a reference copy of the document with all sections, see nature.com/documents/nr-reporting-summary-flat.pdf

Life sciences study design

All studies must disclose on these points even when the disclosure is negative.

Sample size	Taking into consideration '3Rs' guidance in use of animals for scientific research, and an average number of 7 embryos per mouse, the average/standard sample size for mouse studies was n = 3 (pregnant) mice, in order to generate genotype groups of a minimum of 3 embryos per genotype (for experiments in Fig. 1b-g, Fig. 3a-f, Fig. 4c-e). Power calculations indicated that 4 embryos per genotype would be required to detect a difference in fetal liver cell numbers. Experiments were repeated with new timed matings until this minimum embryo number was achieved per genotype. For mechanistic experiments on mTOR activity and autophagy, n=4 mice were used for each condition on the basis of power calculations.
Data exclusions	Supp. Fig. 1g - there is missing data for one Nprl3-/- chimaeric mouse, for which sample collection was not completed on week 12.<br>There is also missing platelet data for some animals. Some samples failed due to flow rate being too high during flow cytometry (this is because platelets and RBCs are so small, sticky and prevalent in even 1ul of diluted blood).<br>The same explanation exists for the missing RBC value in Fig. 1i.
Replication	All experimental repeats were successful, and all repeats are presented in the provided data.
Randomization	Randomisation is not relevant to this study.
Blinding	For protein and metabolite mass spec and RNA-Seq, investigators were blinded during data collection.<br>Otherwise, blinding was not possible as the lead researcher was required to be present for each stage of data collection and analysis.

Reporting for specific materials, systems and methods

We require information from authors about some types of materials, experimental systems and methods used in many studies. Here, indicate whether each material, system or method listed is relevant to your study. If you are not sure if a list item applies to your research, read the appropriate section before selecting a response.

Materials & experimental systems

n/a	Involved in the study
☐	☒ Antibodies
☒	☐ Eukaryotic cell lines
☒	☐ Palaeontology and archaeology
☐	☒ Animals and other organisms
☒	☐ Clinical data
☒	☐ Dual use research of concern
☒	☐ Plants

Methods

n/a	Involved in the study
☒	☐ ChIP-seq
☐	☒ Flow cytometry
☒	☐ MRI-based neuroimaging

Antibodies

Antibodies used

Antibodies grouped by flow cytometry panel
Marker / Fluorophore / Manufacturer / Catalog no.

Fetal liver
Ter119 PE BioLegend 116207
CD71 FITC BD Pharmingen 561936
NK1.1 APC-Cy7 BioLegend 108723
CD90.2 APC-Cy7 BioLegend 105327
B220 APC-Cy7 BioLegend 103224
CD11b APC-Cy7 BioLegend 101226
Gr1 APC/Fire 750 BioLegend 108455

ps6
c-kit BV421 BioLegend 105827
CD71 FITC BD Pharmingen 561936
Ter119 PE BioLegend 116207
pS6 APC Cell Signalling Technologies 4851
B220 APC-Cy7 BioLegend 103224
NK1.1 APC-Cy7 BioLegend 108723
CD90.2 APC-Cy7 BioLegend 105327
Gr1 APC/Fire 750 BioLegend 108455
CD11b APC-Cy7 BioLegend 101226

Progenitors
CD48 PB BioLegend 103417
CD41 BV605 BioLegend 133921
CD34 FITC BD Pharmingen 560238
CD16/32 PercyP Cy5.5 BioLegend 101323
c-kit Biotinylated BioLegend 105803
Streptavidin PE BioLegend 405203
Sca-1 PE-Cy7 BioLegend 108113
CD150 APC BioLegend 115909
B220 APC-Cy7 BioLegend 103224
NK1.1 APC-Cy7 BioLegend 108723
CD90.2 APC-Cy7 BioLegend 105327
Gr1 APC/Fire 750 BioLegend 108455
CD11b APC-Cy7 BioLegend 101226
Ter119 APC-Cy7 BioLegend 116223

Myeloid
CD11b BV421 BioLegend 101235
Ter119 BV510 BioLegend 116237
Ly6C BV605 BioLegend 128035
F4/80 AF488 BioLegend 123119
CD71 PE BioLegend 113807
c-kit PE-Cy7 BioLegend 105813

Ly6G PerCPCy5.5 BioLegend 127615
CD115 APC BioLegend 135509
B220 APC-Cy7 BioLegend 103224
NK1.1 APC-Cy7 BioLegend 108723
CD90.2 APC-Cy7 BioLegend 105327

Autophagy
c-kit BV421 BioLegend 105827
Ter119 PE BioLegend 116207
Sca-1 PE-Cy7 BioLegend 108113
CD71 APC BioLegend 113819
B220 APC-Cy7 BioLegend 103224
NK1.1 APC-Cy7 BioLegend 108723
CD90.2 APC-Cy7 BioLegend 105327
Gr1 APC/Fire 750 BioLegend 108455
CD11b APCCy7 BioLegend 101226

Human
p4E-BP1 PE Cell Signalling Technologies 7547
pS6 APC Cell Signalling Technologies 4851

Fetal liver - bone marrow chimaera
Progenitors
Ter119 BV421 BioLegend 116233
CD16/32 BV510 BioLegend 101333
CD41 BV605 BioLegend 133921
CD45.2 biotin n/a BioLegend 109803
strepdavidin BUV395 BD Biosciences 564176
Sca-1 BV785 BioLegend 108139
CD105 FITC BioLegend 120405
c-kit PE-Cy7 BioLegend 105813
CD150 APC BioLegend 115909
CD90.2 APC-Cy7 BioLegend 105327
B220 APC-Cy7 BioLegend 103224
Nk1.1 APC-Cy7 BioLegend 108723
CD11b APC-Cy7 BioLegend 101226
Ly6C APC-Cy7 BioLegend 128017

Erythroid
Ter119 FITC BioLegend 116205
CD71 PE-Cy7 BioLegend 113811
CD44 APC BioLegend 103011
CD90.2 APC-Cy7 BioLegend 105327
CD11b APC-Cy7 BioLegend 101226
B220 APC-Cy7 BioLegend 103224
Gr1 APC/Fire 750 BioLegend 108455

Validation

All primary antibodies had already been verified by their manufacturer (Biolegend, BD Biosciences, Cell Signalling Technologies or BD Pharmingen).

Animals and other research organisms

Policy information about studies involving animals; ARRIVE guidelines recommended for reporting animal research, and Sex and Gender in Research

Laboratory animals

Three transgenic mouse (Mus musculus) models:
Nprl3-promoter-knockout, CB57BL/6J background, generated in-house
all-enhancer-knockout, CB57BL/6J background, generated in-house
Rosa26tdTomato (B6.Cg-Gt(ROSA)26Sortm9(CAG-tdTomato)Hze Tg(CMV-cre) Ptprca.Pepcb), CB57BL/6J background, gifted in-house, originally Jackson Laboratory
All used aged between 6-18 weeks

Wild animals

This study did not involve wild animals

Reporting on sex

Data reporting on sex differences has not been collected. Female and male mice were both used in this study and similar biological effects were observed in samples collected from male and female mice. We had no reason to believe sex may contribute to phenotype, rather that broad phenotypes exist regardless of sex. Data are reported from male and female mice according to litter distribution (with no obvious phenotypic differences observed), with the exception of bone marrow chimaeras (where it is

	recommended to match donor and recipient to avoid immune reaction). Females used throughout chimaera studies.
Field-collected samples	This study did not involve samples collected in the field
Ethics oversight	Animal work was completed under the authority of UK home office project licenses (P5AC0E8C9 / PP1487090) granted under the Animals (Scientific Procedures) Act (ASPA) 1986.

Note that full information on the approval of the study protocol must also be provided in the manuscript.

Plants

Seed stocks	*Report on the source of all seed stocks or other plant material used. If applicable, state the seed stock centre and catalogue number. If plant specimens were collected from the field, describe the collection location, date and sampling procedures.*
Novel plant genotypes	*Describe the methods by which all novel plant genotypes were produced. This includes those generated by transgenic approaches, gene editing, chemical/radiation-based mutagenesis and hybridization. For transgenic lines, describe the transformation method, the number of independent lines analyzed and the generation upon which experiments were performed. For gene-edited lines, describe the editor used, the endogenous sequence targeted for editing, the targeting guide RNA sequence (if applicable) and how the editor was applied.*
Authentication	*Describe any authentication procedures for each seed stock used or novel genotype generated. Describe any experiments used to assess the effect of a mutation and, where applicable, how potential secondary effects (e.g. second site T-DNA insertions, mosiacism, off-target gene editing) were examined.*

Flow Cytometry

Plots

Confirm that:

- [x] The axis labels state the marker and fluorochrome used (e.g. CD4-FITC).
- [x] The axis scales are clearly visible. Include numbers along axes only for bottom left plot of group (a 'group' is an analysis of identical markers).
- [x] All plots are contour plots with outliers or pseudocolor plots.
- [x] A numerical value for number of cells or percentage (with statistics) is provided.

Methodology

Sample preparation	Provided in methods. Single cell suspensions were made, stained using fluorescent dyes and conjugated antibodies and optionally fixed and permeabilised prior to acquisition
Instrument	Attune NxT / BD LSRFortessa
Software	Attune NxT software and FlowJo version 10
Cell population abundance	Flow cytometry was run on whole fetal liver or bone marrow homogonates, or a dynamic heterogeneous human CD34+ erythroid culture (not pure starting populations, and of varying constitution between samples). S1 and S3 erythroblasts sorted for RNA-Seq, existed as ~40% and 1-5% of the whole population, respectively.
Gating strategy	Gating strategies are illustrated in Supplementary Figure 5

- [x] Tick this box to confirm that a figure exemplifying the gating strategy is provided in the Supplementary Information.
