# Supplementary material for: Ancient genomic linkage of α-globin and Nprl3 couples metabolism with erythropoiesis
Source: Nat Commun. 2025 Mar 24;16:2749. doi: 10.1038/s41467-025-57683-z (PMC11933693; doi:10.1038/s41467-025-57683-z)
Supplement: Supplementary file 3 — Transparent Peer Review file [file 41467_2025_57683_MOESM3_ESM.pdf]

nature portfolio

Peer Review File

Ancient genomic linkage of α-globin and *Nprl3* couples metabolism with erythropoiesis

Corresponding Author: Dr Alexandra Preston

This file contains all reviewer reports in order by version, followed by all author rebuttals in order by version.

Version 0:

Reviewer comments:

Reviewer #1

(Remarks to the Author)
A multicomponent super-enhancer for alpha globin expression exists within the introns of the nearby Nprl3 gene, which encodes a negative regulator of mTORC1 signaling. Nprl3 expression is regulated by the same enhancer and therefore is induced during erythropoiesis. Considering that this relationship between alpha globin and Nprl3 is deeply conserved in evolution, the authors hypothesized that NPRL3 may function in erythroid development, which depends on regulated mTORC1 signaling. Consistent with this model, disruption of the Nprl3 gene promoter in mice, which leaves the super-enhancer intact, caused impairments in erythropoiesis with upregulation of mTORC signaling at baseline, inhibited glycolysis and failure to suppress mTORC signaling after several metabolic deprivations. Similarly, disruption of the NPRL3 gene in human CD34+ cells caused impaired erythropoiesis during in vitro differentiation.

This is an interesting and impactful study supporting the notion that NPRL3 coordinates metabolic needs during erythropoiesis by inhibiting mTORC1. Suggestion for improving this paper include:

Major

1. Nprl3 appears to be required for multiple aspects of hematopoiesis beyond RBC development. While extensive characterization of Nprl3 loss throughout hematopoiesis is beyond the scope of this paper, the authors could extend some of their studies to address this point. Suggestions include:

- Figure 1i: Provide similar graphs showing Nprl3-/- fetal liver donor cell (tomato negative) contribution to circulating granulocytes, lymphocytes and platelets after transplantation.

- Figure 4: Determine whether any non-erythroid progenitors are altered in Nalph mice, similar to what was done in Figure S1, panel f. This experiment would determine whether “baseline” (non-enhancer driven) Nprl3 expression is sufficient for non-erythroid lineages.

2. Figure 2c-f. Please indicate at what time during erythroid culture was mTORC1 activity assessed. Can the authors estimate which progenitors are being observed? Perhaps they could co-stain the cells with erythroid maturation markers to assess mTORC activities at different stages of erythropoiesis.

3. A few additional studies could better define Nprl3 gene regulation by the alpha-globin enhancer. Specifically:

- Figure 4c should include Nprl3-/- erythroid cells as a negative control.

-Quantify mRNAs for Nprl3, alpha globin and a control gene(s) in purified erythroid progenitors at defined developmental stages from fetal liver of WT, Nprl3 KO, Nprl3+/- and Nalph mice.

-Compare the levels of Nprl3 expression in erythroid cells and non-erythroid cells? The bloodspot database [https://www.bloodspot.eu/] and perhaps Extended Data Figure 1g indicate that Nprl3 expression is high in some non-erythroid lineages. Nprl3 expression in the latter should not be affected by enhancer knockout.

Minor

1. Extended data Fig. 1g should be marked to show the positions of HSCs and erythroid precursors to better support the linked statement in the text.

2. Figure S2a should show targeting data for sgRNA3. Methods section shows sequences for sgRNA 1 and 3 but not sgRNA2. It might be helpful to include a diagram of the human NPRL3 locus showing the positions of the sgRNAs and the gene exons and the enhancer elements.

3. The negative control (NC) should be explained in the legend of Figure 2 and in the main text.

4. Figure 4: Please note in legend and corresponding text the embryonic age of fetal liver analyzed.

Reviewer #2

(Remarks to the Author)
This study probes the role of Nprl3, a key negative regulator of the mTORC1 pathway, in the setting of red blood cell development, motivated by the intriguing observation that this gene has remained in synteny across evolution with the alpha-globin genes. The authors convincingly establish that loss of Nprl3 results in a severe defect in red blood cell development and their cellular metabolism, and that these effects are mediated intrinsically. The authors need to address what aspects of dysregulated cellular metabolism are critical for red blood cell development through targeted rescue experiments. Moreover, whether this synteny is functionally relevant needs further investigation. The authors should address if the effects they observe on hematopoiesis are due to this synteny or due to the effect that their AEKO mouse model has on globin gene transcription. Finally, the authors need to show validation of their mouse model at the Nprl3 protein level, and they must present their western blots with the proper controls.

1. In Figure 1, the authors should validate that their Nprl3 KO mouse line leads to a loss in Nprl3 protein levels for accurate interpretation that the effects they observe on red blood cell development are indeed due to a loss of Nprl3 levels.

2. For the claims that Nprl3 loss in CD34+ HPSCs lead to overactive mTORC1 activity in Figure 2, the authors need to show a representative western blot for each of the panels in c, d, e, f, and g, and not simply the summarized quantification. These blots should also include total S6 protein as well as a loading control for proper interpretation that the manipulation affects pS6 and not the levels of S6 or total protein in the sample. Likewise, in Figure 3a, they should also show the western blot with the aforementioned loading controls. In particular, there is very high variability in the pS6 signal of one of the Nprl3-/- litters.

3. In Figure 2, the authors should demonstrate that the effect of Nprl3 loss on mTORC1 activity is specific to its integration of amino acid inputs. A key input into the pathway is serum/growth factors. if the pathway functions in a canonical way in this cell type, loss of serum input to the CD34+ HPSCs should abolish the pS6 signal.

4. Nprl2 is in a well-established complex with Nprl3 and Depdc5 to form GATOR1, which altogether negatively regulates mTORC1 activity. In Figure 2, the authors assess Nprl3 and Nprl2, but omit Depdc5. What is the rationale for this omission? To understand how Nprl2 and Nprl3 regulate the mTORC1 activity in these cells, the authors should address if Depdc5 is also involved in this process, or whether there is a non-canonical mechanism of mTORC1 regulation that depends only on Nprl2 and Nprl3?

5. In Figure 3, dysregulated mTORC1 signaling has a multitude of effects on metabolism, which is consistent with its established role in multiple aspects of cellular metabolism. The authors should evaluate which of these output(s) of mTORC1 are responsible for the effects on erythroid differentiation? Would an upregulation of autophagy, antioxidant response, or glycolysis in the context of Nprl3 loss help to rescue these defects? For instance, serum deprivation is likely to trigger the onset of autophagy by inhibiting mTORC1 activity despite Nprl3 loss. Would this help to rescue the defects?

6. The effects on metabolism stemming from Nprl3 loss in the fetal liver would benefit greatly from a more global assessment of metabolic changes via metabolomics rather than selecting a subset of metabolites, which can introduce bias as well as cause the authors to miss key or unexpected effects that this dysregulation could have, especially in the context of this specialized cell type.

7. Given the role of Nprl3 in responding to and controlling amino acid levels and metabolism, it is surprising that there are no significant changes in amino acid levels in these Nprl3-/- cells in Extended Figure 3. Previous studies with another GATOR1 component, DEPDC5, show dramatic differences in vivo upon GATOR1 loss (Yuskaitis et al, 2022). The authors should comment on this discrepancy.

8. The authors cross-bred Nprl3+/- and Nprl3+/AEKO mouse lines to assess the role of globin enhancers on Nprl3 mRNA transcription. The authors conclude that the Nprl3+/AEKO genotype “accounts for possible indirect effects of the enhancer deletions on Nprl3 expression, showing no significant difference in Nprl3 expression compared to Nprl3+/+ and Nprl3+/- lines”. However, in Figure 4c, there is a clear outlier in the Nprl3+/AEKO group. Moreover, if one compares the Nprl3+/- and Nprl3AEKO/- lines, the only difference between them is the AEKO allele, and this allele leads to a large reduction in Nprl3

transcription. Given this observation, wouldn’t Nprl3 transcript levels be expected to be reduced in the Nprl3+/AEKO mouse line?

9. In Figure 4c, what accounts for the highly variable Nprl3 mRNA expression in the Nprl3+/- mouse line?

10. In Extended Data Figure 4B, the Pol-II ChIP-Seq shows that not only is Pol II loading impaired on the Nprl3 promoter, but also at the Hba-a1 and Hba-a2 loci in the “All enhancer KO allele”. Given this observation, are the effects that the authors observe on hematopoiesis in Figure 4 and Extended Figure 4 specific to the downregulation of Nprl3 expression? This is a difficult claim to make given that hemoglobin gene transcription is also affected. To interpret their findings, the authors need to demonstrate that globin protein expression is not impaired in this mouse model or that loss of globin gene expression cannot explain the defects in red blood cell development that they observe.

11. In Figure 4D, there is a modest reduction in absolute numbers of RBCs in the Nprl3+/AEKO line, especially considering the extreme loss of Nprl3 transcription observed in Figure 4C. Is this because Nprl3 protein is still present and regulation of mTORC1 signaling is still present? The authors should probe mTORC1 activity and Nprl3 protein levels across the four genotypes in Figure 4 to interpret their results.

Reviewer #3

(Remarks to the Author)
This is a well written paper focused on elucidating the function of Nprl3 in erythropoiesis. Nprl3 is a gene syntenic with the alpha globin locus over a broad swath of evolution. The authors convincingly determine that Nprl3 regulates the metabolic state of erythroid precursors through the mTORC1 signaling pathway specifically in the setting of amino acid, iron and EPO deprivation. Studies are conducted both in vivo in a murine knockout model and in human CD34+-derived HSPCs cultured in vitro. Additionally, generic studies using complex mouse models indicate that the alpha globin enhancers also regulate Nprl3 expression. Taken together, this manuscript presents significant new data that the alpha globin enhancers couple Nprl3-regulated metabolism with globin gene expression during terminal stages of erythropoiesis. This serves as an intriguing example of an evolutionarily conserved enhancer sharing mechanism. The study limitations are acknowledged.

Further comments:

Major:
1. The studies of Nprl3 knock-out mouse embryos focus on the E13.5 fetal liver revealing significant defects in definitive erythropoiesis but not in concomitant HSPCs, CMP, and GMP. The relatively late in utero death of Nprl3-null embryos is consistent with a significant defect in definitive erythropoiesis. Does Nprl3 play a role in primitive erythropoiesis? Is it expressed in primitive erythroblasts? Are Nprl3 knock-out mouse embryos anemic at E12.5, i.e., before significant numbers of definitive cells have entered the circulation? Is Nprl3 regulation connected to the alpha-globin enhancers that regulate zeta-globin?

Minor:
1. Second paragraph, “erythroblasts undergo rapid haemaglobinisation followed by high autophagic and proteosomal activity”, and first paragraph of the Discussion describing anabolic and subsequent catabolic phases. Is there evidence that these are two separate phases over the ~3 terminal erythroblast cell divisions or do they occur simultaneously during terminal erythroid differentiation?

2. In contrast to the findings in the fetal of Nrpl3 knock-out mice, in chimeric adult mice Nprl3-null cells do not contribute well to the broad HSPC compartment, suggesting defects at that level in HSC-derived hematopoiesis, as well as in terminal erythropoiesis. Despite this defect and the severe defect in BasoE and subsequent stages of erythropoiesis (Extended Data, Fig. 1f), there are normal numbers of CFU-E/ProE. Can the authors speculate how this might be possible?

3. Nprl3 knock-out erythroid cells have decreased glutathione levels. Do they contain increased ROS levels or do other antioxidant pathways compensate?

4. The study of Nprl3 levels and Nrlp3 promoter-enhancer interactions in erythroid cells from the fetal livers of transgenic mice support the regulation of Nprl3 expression by the alpha-globin enhancers. The decreased hemoglobin and MCV of Nprl3+/AEKO mice are certainly consistent with alpha-thalassemia. Are there comparative data available from alpha-thalassemia mice lacking 2 of the 4 alpha-globin genes?

Version 1:

Reviewer comments:

Reviewer #1

(Remarks to the Author)
Overall, this is an interesting study that identifies “housekeeping” and erythroid specific functions for the mTORC1 inhibitor Nprl3 in hematopoiesis. In general, I am satisfied with the authors efforts to address reviewer comments. However, not all of the comments could be addressed, in part due to technical difficulties, unavailable or faulty reagents and the complexities of

mTORC1 signaling. For many experiments, the numbers of mice analyzed are relatively low and there is considerable phenotype variability, possibly due to variable metabolic conditions, as the authors note in their rebuttal. The authors provide evidence for accelerated glycolysis and impaired autophagy, both of which are known consequences of mTORC activation. However, there is no experimental evidence that either of these abnormalities contribute to the observed impairment in erythropoiesis. The authors also note that studying mTORC signaling and its effects on cells in vitro is difficult due to variables that are difficult to control for. Several reviewer comments/critiques are answered in the rebuttal but not addressed in the revised manuscript. To address these issues, I suggest that the authors add a paragraph to the discussion reviewing limitations of the study, including some of the questions/problems cited by the reviewers.

A few additional comments/suggestions:

1. The abstract could better explain one of the author's major points -- that Nprl3 is required for all hematopoietic lineages (possibly due to a requirement in hematopoietic stem/progenitor cells), but an extra "boost" from the alpha globin enhancers is required specifically for erythroid cells.

2. Change text accompanying Figure 4f-h (line 268, page 6) to: "This viable level of alpha-globin mRNA is not further reduced by the co-presence of Nprl3+/- (as in Nalph) in cis…"

3. Line 331, page 7. Authors state: "Therefore, it is likely that disruption of glycolysis due to mTORC1 overactivation contributes to the Nprl3-/- erythroid differentiation defect". However, glycolysis thought to be upregulated, not disrupted. This point should be clarified I the text. How might activation of glycolysis inhibit erythropoiesis? Is there evidence in the literature to support this? Have the authors actually measured glycolysis in the mutant erythroid cells?

(Remarks on code availability)

Reviewer #2

(Remarks to the Author)
My main concerns were addressed by the authors, and I commend them for doing so.

First, I was concerned about the lack of validation that Nprl3 levels were reduced in their Nprl3-/- mouse line. I appreciate the authors' efforts to find an antibody that works, and their data that Nprl3 mRNA levels are greatly decreased in Ext. Data Figure 4 is an important addition.

There is high variability on the effect of mTORC1 activity with the Nprl3-/- mice in Figure 2, but the information the authors now include about the litter identity clarifies confusion, especially in combination with the other findings of how Nprl3 loss affects responsiveness to arginine and leucine deprivation.

The authors presented a more unbiased view of the metabolic effects of Nprl3 loss with RNA seq, which more convincingly pinpoint the defects in glycolysis than only targeted metabolomics. I understand the difficulties of performing more ideal rescue experiments and mechanistic work in their system, and I hope that the authors will follow-up these findings in another model system that is subject to less variability and will be more amenable to biochemical and genetic manipulation.

Finally, the authors addressed the validity of their Nalph model in Figure 4. Their corrections of misplotted data with necessary measurements of alpha globin levels make a stronger case that the effects observed on S3 erythroblast numbers are not due to an effect in alpha globin expression but rather the reduction of Nprl3 in this model.

(Remarks on code availability)

Reviewer #3

(Remarks to the Author)
The authors have very effectively responded to the critiques and Issues raised by the reviewers of the initial submission. Importantly, they have performed additional experiments and added new data both to the primary and to the extended figures. The authors have also appropriately modified the text of the manuscript to reflect the new data. Additionally, the text (particularly the Discussion) has been modified in response to other points/questions raised in the reviews. Overall, these additions and changes further strengthen an already strong paper.

This manuscript presents significant new data that the alpha globin enhancers couple Nprl3-regulated metabolism with globin gene expression during terminal stages of erythropoiesis. This serves as an intriguing example of an evolutionarily conserved enhancer sharing mechanism.

(Remarks on code availability)

Version 2:

Reviewer comments:

Reviewer #1

(Remarks to the Author)
The authors have responded to most of my queries and the queries from other reviewers. A few questions poised by the reviewers remain unanswered due to various problems including difficulties producing mice, lack of reagents, etc. It might be helpful to add a section in the discussion about open questions and limitations of the current study.

Open Access This Peer Review File is licensed under a Creative Commons Attribution 4.0 International License, which permits use, sharing, adaptation, distribution and reproduction in any medium or format, as long as you give appropriate credit to the original author(s) and the source, provide a link to the Creative Commons license, and indicate if changes were made.
In cases where reviewers are anonymous, credit should be given to 'Anonymous Referee' and the source.
The images or other third party material in this Peer Review File are included in the article's Creative Commons license, unless indicated otherwise in a credit line to the material. If material is not included in the article's Creative Commons license and your intended use is not permitted by statutory regulation or exceeds the permitted use, you will need to obtain permission directly from the copyright holder.
To view a copy of this license, visit https://creativecommons.org/licenses/by/4.0/

Nature Communications Reviews

Reviewer 1

A multicomponent super-enhancer for alpha globin expression exists within the introns of the nearby Nprl3 gene, which encodes a negative regulator of mTORC1 signaling. Nprl3 expression is regulated by the same enhancer and therefore is induced during erythropoiesis. Considering that this relationship between alpha globin and Nprl3 is deeply conserved in evolution, the authors hypothesized that NPRL3 may function in erythroid development, which depends on regulated mTORC1 signaling. Consistent with this model, disruption of the Nprl3 gene promoter in mice, which leaves the super-enhancer intact, caused impairments in erythropoiesis with upregulation of mTORC signaling at baseline, inhibited glycolysis and failure to suppress mTORC signaling after several metabolic deprivations. Similarly, disruption of the NPRL3 gene in human CD34+ cells caused impaired erythropoiesis during in vitro differentiation.

This is an interesting and impactful study supporting the notion that NPRL3 coordinates metabolic needs during erythropoiesis by inhibiting mTORC1. Suggestion for improving this paper include:

Major

1. Nprl3 appears to be required for multiple aspects of hematopoiesis beyond RBC development. While extensive characterization of Nprl3 loss throughout hematopoiesis is beyond the scope of this paper, the authors could extend some of their studies to address this point. Suggestions include:

- Figure 1i: Provide similar graphs showing Nprl3-/- fetal liver donor cell (tomato negative) contribution to circulating granulocytes, lymphocytes and platelets after transplantation.

Thank you for this request. We have incorporated graphs into Extended Data Fig. 1 (panel g) indicating the impaired contribution of *Nprl3*$^{-/-}$ fetal liver cells to mature myeloid cells, B cells, CD4 and CD8 T cells and platelets.

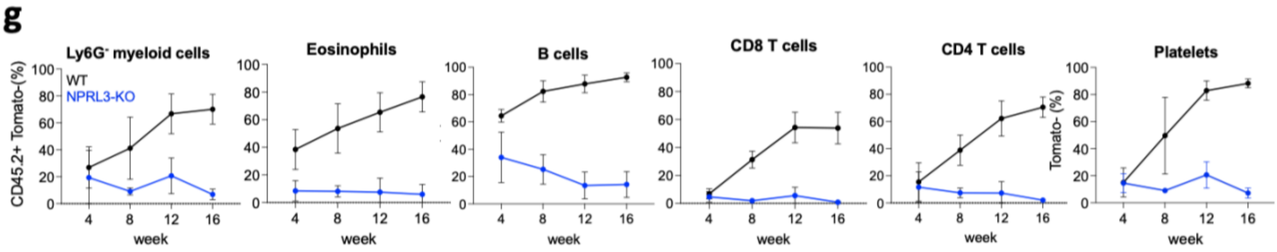

- Figure 4: Determine whether any non-erythroid progenitors are altered in Nalph mice, similar to what was done in Figure S1, panel f. This experiment would determine whether "baseline" (non-enhancer driven) Nprl3 expression is sufficient for non-erythroid lineages.

Thank you for this insightful suggestion. Please see the graph below, showing measurement of GMP, CMP and MEP numbers in *Nalph* mice and littermates. This indeed suggests that baseline / ‘unenhanced’ *Nprl3* expression is sufficient for haematopoiesis upstream of terminal erythroid differentiation. We have included this as a figure panel (Fig. 4e).

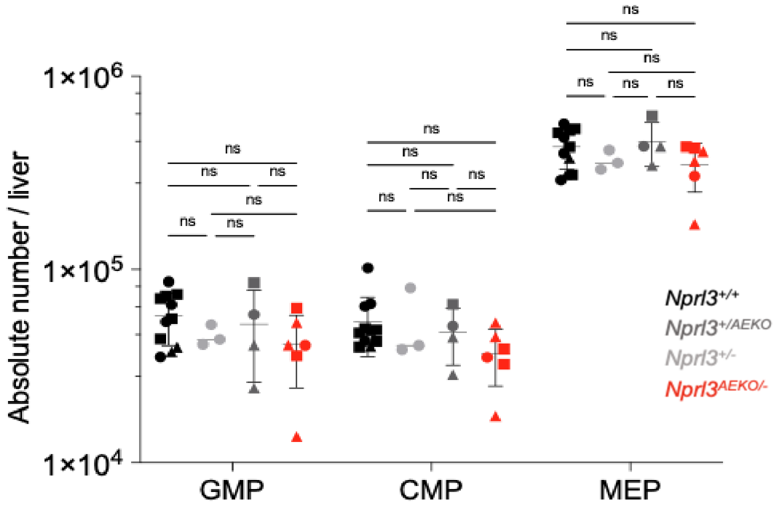

Added in-text interpretation:
‘Note that the numbers of haematopoietic progenitors (GMP, CMP and MEP) were not different between genotypes (Fig. 4e), indicating an erythroid-specific requirement of enhanced *Nprl3* transcription.’

2. Figure 2c-f. Please indicate at what time during erythroid culture was mTORC1 activity assessed. Can the authors estimate which progenitors are being observed? Perhaps they could co-stain the cells with erythroid maturation markers to assess mTORC activities at different stages of erythropoiesis.

Thank you, p4E-BP1 and pS6 were measured on day 11 of the CD34 erythroid culture system (now stated in the figure legend). At this time, the majority of the population are basophilic and polychromatic erythroblasts (see representative cytospins below). These proportions are not significantly different between genotypes, though the reduced ultimate production of enucleated cells is notable (Fig. 2b, Extended Data Fig. 2c).

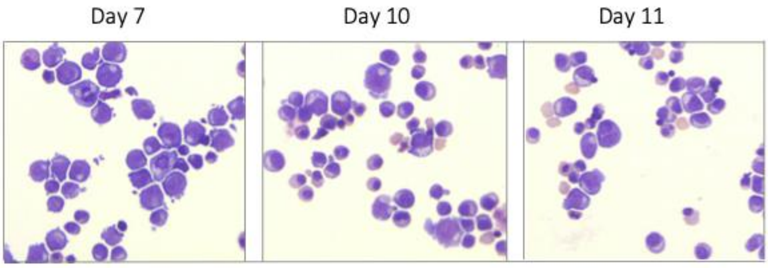

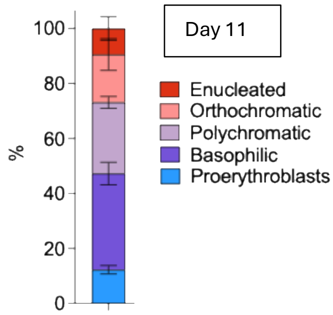

We have not performed erythroblast-stage specific analysis because effective use of surface markers to define staged erythroblasts in this system is challenging. Unlike in mouse, markers of human erythroid maturation change along a continuum (McGrath, Catherman and Palis, *https://doi.org/10.1016/j.ymeth.2016.08.012*).

3. A few additional studies could better define Nprl3 gene regulation by the alpha-globin enhancer. Specifically:

- Figure 4c should include Nprl3-/- erythroid cells as a negative control.

We have now performed the ddPCR assay with *Nprl3*$^{-/-}$ cells, confirming the complete absence of detectable *Nprl3* transcription in this model. This data has been incorporated as Extended data Fig. 4a:

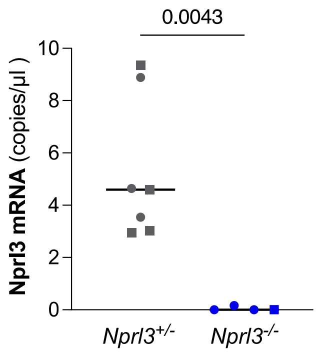

-Quantify mRNAs for Nprl3, alpha globin and a control gene(s) in purified erythroid progenitors at defined developmental stages from fetal liver of WT, Nprl3 KO, Nprl3+/- and Nalph mice.

As above, *Nprl3* mRNA is now presented for all genotypes. We agree that globin expression is an important control to present for all genotypes. This data has also now been gathered and is presented in Extended Data Fig. 4 (f/g/h).

Accompanying manuscript text:
As α-globin enhancers are required for α-globin expression on the allele *in cis*, *Nprl3*$^{+/AEKO}$ is equivalent to the expression of 2 of 4 functional α-globin genes (such as in α^0-thalassaemia carriers and alpha-thalassemia mice, including Hba^{th-J} ($^{--}$/^{aa})). Due to the duplication of α-globin, such patients are often asymptomatic and their erythropoiesis is sufficient overall[39]. This viable level of α-globin mRNA is not further reduced by the co-presence of *Nprl3*$^{+/-}$ (as in *Nalph*), confirming that the *Nalph* phenotype is not driven by alterations to globin expression (Extended Data. Fig. 4h).

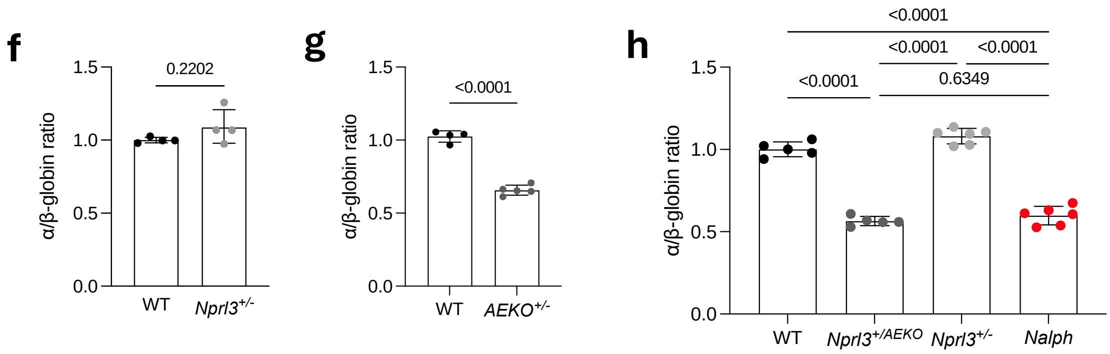

Unfortunately, due to the limited number of S0/S1/S2 cells per fetal liver, we have only been able to measure *Nprl3* and globin in S3 erythroblasts.

-Compare the levels of Nprl3 expression in erythroid cells and non-erythroid cells? The bloodspot database [https://www.bloodspot.eu/] and perhaps Extended Data Figure 1g indicate that Nprl3 expression is high in some non-erythroid lineages. Nprl3 expression in the latter should not be affected by enhancer knockout.

Thank you for this thought. It is difficult to accurately compare pre-existing data regarding erythroid and non-erythroid *Nprl3* expression, because enhancer-derived eRNAs likely confound erythroid measurements (which is why we used ddPCR to define promoter-derived mRNA).

However, it is interesting to compare *Nprl3* expression levels between non-erythroid lineages. As illustrated by the scRNA-Seq U-map below (from human fetal liver, data derived from CellAtlas.io tool, developed by the Haniffa lab, with searchable transcriptional expression in the human yolk sac https://app.cellatlas.io/fetal-liver/dataset/3/scatterplot), non-erythroid haematopoietic cell types express *Nprl3* within a comparable range (but lower than erythroid).

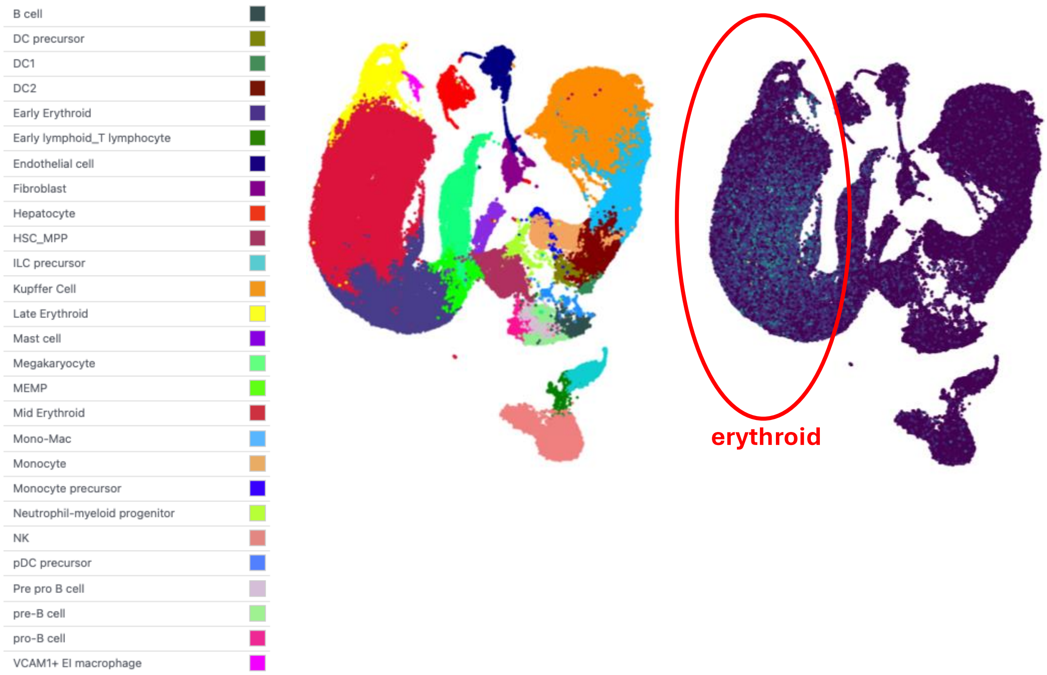

The 'mouse normal haematopoietic system' data (bone marrow; microarray) accessible through the bloodspot tool suggest that ProB cells and CD8 T cells may express slightly higher *Nprl3* than other hamatopoieic lineages (see below).

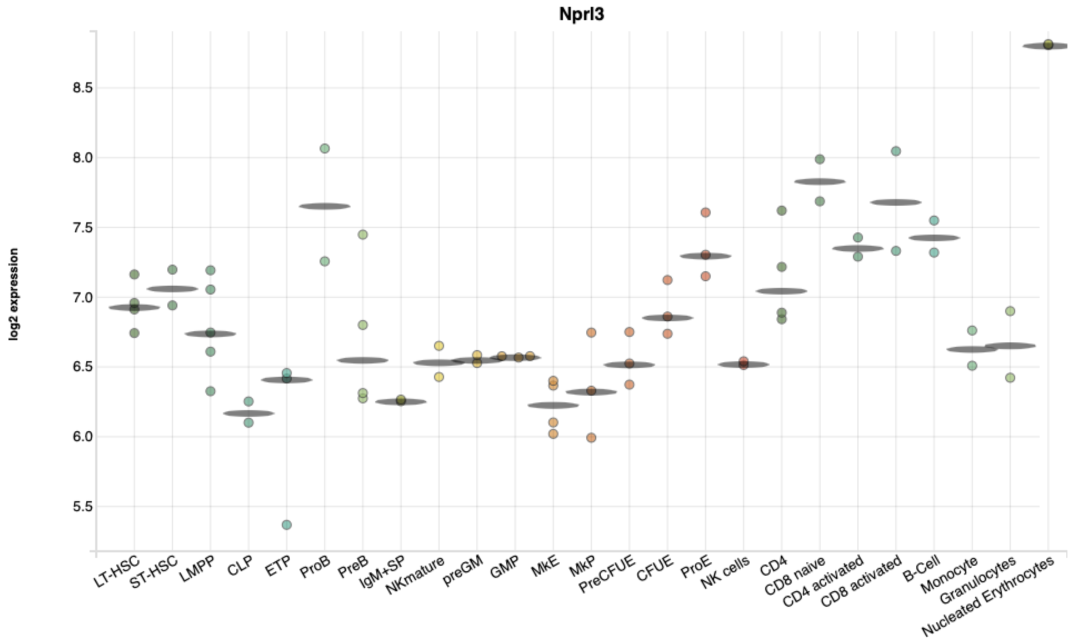

Meanwhile, scRNA-Seq of mouse bone marrow suggests that HSCs express relatively high levels of Nprl3 (Ext Data Fig. 1h).

It is true that *Nprl3* transcription would not be affected by α-globin enhancer-knockout in non-erythroid lineages, where the enhancers are not 'open' (α-globin enhancer activity is erythroid-specific - PMID: 15215894, PMID: 28737770).

It would be interesting to definitively describe *Nprl3* levels among haematopoietic lineages using the ddPCR assay we introduce in this study. Unfortunately, we do not have sufficient cellular material to perform our ddPCR assay on non-erythroid lineages, and also suggest that this is beyond the scope of this study.

Minor

1. Extended data Fig. 1g should be marked to show the positions of HSCs and erythroid precursors to better support the linked statement in the text.
Thank you, this has been done, and legend now reads 'The spatial positions of HSCs and erythroid precursors are indicated'.

2. Figure S2a should show targeting data for sgRNA3. Methods section shows sequences for sgRNA 1 and 3 but not sgRNA2. It might be helpful to include a diagram of the human NPRL3 locus showing the positions of the sgRNAs and the gene exons and the enhancer elements.
Apologies, sgRNA3 was mislabelled in Fig. S2a. Only two sgRNAs were selected for use in this study. For clarity, we have renamed sgRNA3 as sgRNA 2. Both sgRNAs cut in exon 4 of *Nprl3*.

3. The negative control (NC) should be explained in the legend of Figure 2 and in the main text.
This has been done. 'NC (negative control) represents a scrambled non-targeting sgRNA'.

4. Figure 4: Please note in legend and corresponding text the embryonic age of fetal liver analyzed.
Thank you - now included (always E13.5).

Reviewer 2

This study probes the role of Nprl3, a key negative regulator of the mTORC1 pathway, in the setting of red blood cell development, motivated by the intriguing observation that this gene has remained in synteny across evolution with the alpha-globin genes. The authors convincingly establish that loss of Nprl3 results in a severe defect in red blood cell development and their cellular metabolism, and that these effects are mediated intrinsically. The authors need to address what aspects of dysregulated cellular metabolism are critical for red blood cell development through targeted rescue experiments. Moreover, whether this synteny is functionally relevant needs further investigation. The authors should address if the effects they observe on haematopoiesis are due to this synteny or due to the effect that their AEKO mouse model has on globin gene transcription. Finally, the authors need to show validation of their mouse model at the Nprl3 protein level, and they must present their western blots with the proper controls.

1. In Figure 1, the authors should validate that their Nprl3 KO mouse line leads to a loss in Nprl3 protein levels for accurate interpretation that the effects they observe on red blood cell development are indeed due to a loss of Nprl3 levels.

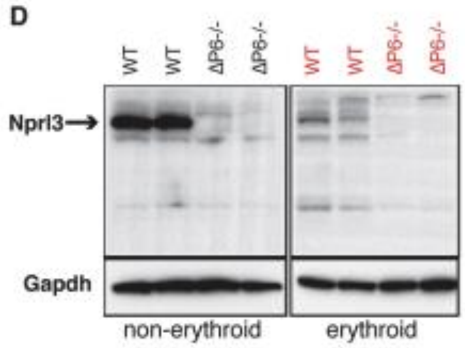

Please note that this mouse line was characterised in a previous study from our institute (doi.org/10.1016/j.molcel.2011.12.021). In this work, loss of Nprl3 protein upon deletion of the *Nprl3* promoter ($\Delta P6^{-/-}$) is demonstrated by western blot, see right. The mouse line was rederived from cryopreservation for the current study.

In this model, a 2.3-kb section of DNA is absent, spanning the entire promoter region. See below schematic in WT, with an enrichment of ATAC-Seq reads indicating the transcriptional start site of *Nprl3,* and the region of the promoter deletion indicated by the blue box (2.3kb).

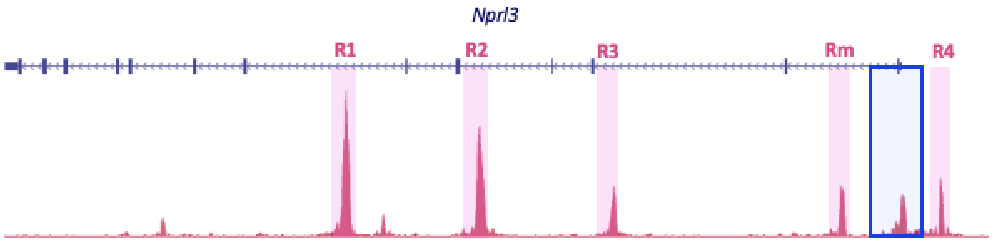

We have now confirmed the total absence of detectable *Nprl3* transcription in the presence of this promoter deletion using digital droplet PCR (which measures absolute transcript abundance). Please see below the lack of detectable *Nprl3* mRNA transcript in *Nprl3*$^{-/-}$ cells (now Extended Data Fig. 4a – different symbols represent mice from different litters).

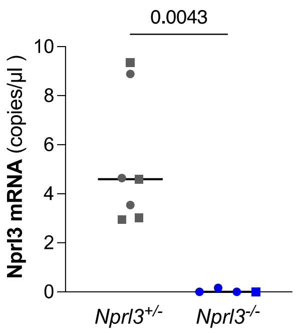

Added manuscript text:
'This assay was validated using *Nprl3*$^{-/-}$ cells, indicating total loss of *Nprl3* expression with promoter deletion (Extended Data. Fig. 4a).'

We had hoped to perform further western blot studies, but multiple commercially available antibodies did not produce bands from WT cells at the correct molecular weight. We also contacted the researcher who synthesized the custom antibody for the blot pictured above, c. 2010. This was located in their -20°C-freezer and was kindly shipped to us from Italy. Unfortunately, we found the antibody to be degraded and so could not be used.

2. For the claims that Nprl3 loss in CD34+ HPSCs lead to overactive mTORC1 activity in Figure 2, the authors need to show a representative western blot for each of the panels in c, d, e, f, and g, and not simply the summarized quantification. These blots should also include total S6 protein as well as a loading control for proper interpretation that the manipulation affects pS6 and not the levels of S6 or total protein in the sample. Likewise, in Figure 3a, they should also show the western blot with the aforementioned loading controls. In particular, there is very high variability in the pS6 signal of one of the Nprl3-/- litters.

In Figure 2, pS6 protein was measured by flow cytometry, not western blotting, using an established approach (e.g. PMID: 35793635, PMID: 32219446, PMID: 27601261, PMID: 32024827). We aimed to show (on a per cell basis) high mTORC1 activation in *Nprl3*$^{-/-}$ conditions compared to control**,** using pS6 as a well-characterised reporter for mTORC1 activity.

It is true that the degree of pS6 upregulation in *Nprl3*$^{-/-}$ is variable between litters. We have updated Fig. 3a to show each analysed embryo from each litter (represented by shape), to indicate that this variability exists predominantly between litters and typically less so within litters. Inter-litter variability in pS6 signal could result from nutritional state of the dam or differences in stress levels between dams, and within a litter depending on position of the embryo within the uterine horn.

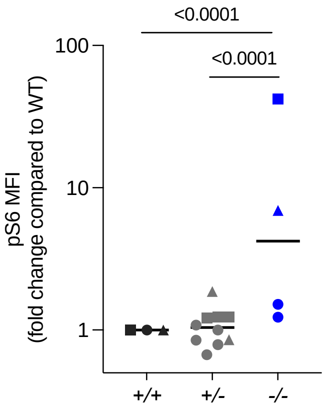

3. In Figure 2, the authors should demonstrate that the effect of Nprl3 loss on mTORC1 activity is specific to its integration of amino acid inputs. A key input into the pathway is serum/growth factors. if the pathway functions in a canonical way in this cell type, loss of serum input to the CD34+ HPSCs should abolish the pS6 signal.

Thank you for this consideration. It is already characterised that there are distinct pathways of mTORC1 activation by amino acids and growth factors. Signalling of growth factors (such as insulin) to receptor tyrosine kinases activates mTORC1 via AKT and the TSC-Rheb regulatory pathway (PMID: 12172553, PMID: 12150915, PMID: 12172554). Meanwhile, amino acids (and not growth factors) signal to Rheb via GATOR-1 (including Nprl3) and the Rag GTPases (PMID: 18497260). Amino acid signalling to Rag GTPase proteins is required for localisation of mTORC1 to the lysosome for activation (PMID: 18497260). Importantly, the TSC1/TSC2 complex is not required for the regulation of mTORC1 by amino acids (PMID: 157720760).

Therefore, on the basis of this literature, we considered that the approach to testing whether Nprl3 signals canonically in erythroblasts (which has not been described previously) was to measure mTORC1 activation following amino acid withdrawal, and not serum withdrawal. Hence, this was addressed by our experiments presented in Fig. 2g, aiming to demonstrate that Nprl3 was acting canonically according to expectations of its role in GATOR1.

4. Nprl2 is in a well-established complex with Nprl3 and Depdc5 to form GATOR1, which altogether negatively regulates mTORC1 activity. In Figure 2, the authors assess Nprl3 and Nprl2, but omit Depdc5. What is the rationale for this omission? To understand how Nprl2 and Nprl3 regulate the mTORC1 activity in these cells, the authors should address if Depdc5 is also involved in this process, or whether there is a non-canonical mechanism of mTORC1 regulation that depends only on Nprl2 and Nprl3?

Apologies for any confusion, but we have not investigated (or mentioned) Nprl2 in this study. For your interest, there has been a haematopoietic study of Nprl2 in mouse (PMID: 26166573), in which they associate loss of Nprl2 with cobalamin deficiency and altered methionine metabolism. The erythropoietic perturbation in *Nprl2*-deficient mice is less severe compared to *Nprl3$^{-/-}$*, not causing the differentiation block at S3. *Depdc5$^{-/-}$* mice demonstrate mTORC1 hyperactivation and a complex embryonic lethal phenotype that was reported to include anaemia, although the nature of the erythropoietic defect was not investigated (PMID: 28974734). Meanwhile, germ line deletion of *Tsc1$^{-/-}$* (not part of the Gator-1 complex) is frequently associated with neural tube disclosure, without reported erythroid phenotype (PMID: 11438694). This may support a relative erythroid-specific importance of Gator-1-mediated regulation of mTORC1, but this remains speculative.

Our rationale for focussing on *Nprl3* was provided by its genomic location and its potential regulation alongside alpha globin (now shown herein).

5. In Figure 3, dysregulated mTORC1 signaling has a multitude of effects on metabolism, which is consistent with its established role in multiple aspects of cellular metabolism. The authors should evaluate which of these output(s) of mTORC1 are responsible for the effects on erythroid differentiation? Would an upregulation of autophagy, antioxidant response, or glycolysis in the context of Nprl3 loss help to rescue these defects? For instance, serum deprivation is likely to trigger the onset of autophagy by inhibiting mTORC1 activity despite Nprl3 loss. Would this help to rescue the defects?

Thank you for this consideration. We aimed to disentangle the multiple effects of mTORC1 overactivation through rescue experiments and assign different processes a contribution to the overall phenotype. However, this has proven to be very difficult, for practical experimental reasons.

The desirable experimental approach for such phenotype rescues would be to culture WT and *Nprl3$^{-/-}$* erythroid cells from fetal liver *ex vivo*, and test pathway-specific interventions to return the *Nprl3$^{-/-}$* phenotype to WT. Unfortunately, in culture, *Nprl3$^{-/-}$* erythroblasts differentiate and grow very similarly comparably to WTs (see graph below), and do not recapitulate the severe *in vivo* block at S3. This may be because under the nutrient- and growth-factor-rich culture conditions required to promote high efficiency erythroid cell proliferation and differentiation, mTORC1 is highly activated and so loss of the inhibitor *Nprl3* does not substantially alter cellular

state. In contrast, *in vivo,* Nprl3-dependent regulation of mTORC1 is critical for erythropoiesis, likely because numerous simultaneous developmental processes are ongoing in the physiological nutritional conditions of pregnancy. This result means an easily tractable *in vitro* approach was not available to us.

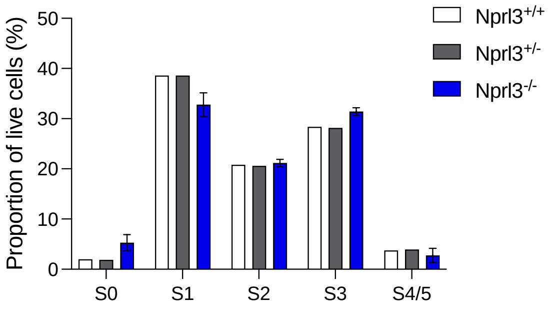

We next attempted complete phenotype reversal *in vivo*. Instead of targeting particular metabolic processes, we began with full mTORC1 inhibition using rapamycin, to test proof-of-principle. Please see the below data indicating a possible but incomplete rescue of the erythroid S3 depletion phenotype of *Nprl3*$^{-/-}$ embryos following rapamycin administration to pregnant mice (each mother is treated as an experimental unit due to drug administration, and is represented by a symbol). Rapamycin treatment was administered at 0.3mg/kg on day E10.5, and 0.1mg/kg on E11.5 and E12.5. Rapamycin was delivered intraperitoneally, and so will affect both maternal and fetal cell types, and the placenta. Thus, the inability to target fetal erythroblasts of the appropriate stage makes such experiments difficult to interpret. Given that we only observe a partial effect with direct mTORC1 inhibition, we are less likely to observe rescue with other perturbations *in vivo*.

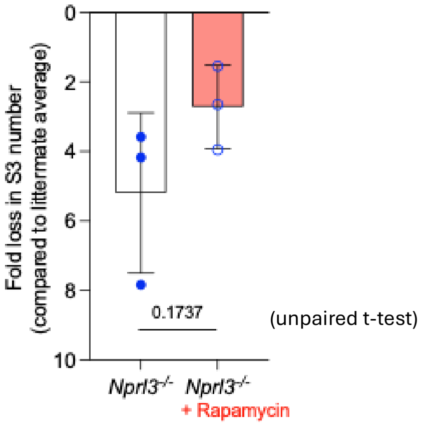

Due to these findings, and the breadth of processes downstream of mTORC1 signalling, we have not been able to decipher a linear or causative mechanism behind the erythroid phenotype of *Nprl3*$^{-/-}$. Therefore, in an effort to build a fuller picture of changes underlying the *Nprl3*$^{-/-}$ phenotype *in vivo*, we performed RNA-Seq on stage-matched S1 and S3 erythroblasts and proteomics on S3 erythroblasts from WT and *Nprl3*$^{-/-}$ mice.

These new data (see Fig 3e, g; Extended Data Figure 3c,d,e,f) show transcriptomic and protein-level upregulation of glycolytic enzymes that control production of some of the metabolites we showed were dysregulated in *Nprl3*$^{-/-}$ erythroblasts (Fig 3d). Please note increased transcription of Aldolase 1, which regulates conversion of hexose bisphosphate to DHAP and 3-phosphoglycerate, and protein/mRNA upregulation of phosphofructokinase, the 'gatekeeper of glycolysis' which catalyses hexose phosphate > hexose bisphosphate. Both RNA-Seq and proteomics revealed general upregulation of enzymes in glycolysis, with 4 overlapping hits. The combined outputs of 3 omics approaches provide strong evidence for disrupted glycolysis is a significant alteration in *Nprl3*$^{-/-}$ erythropoiesis. This is important as glycolysis is a critical metabolic pathway for developing erythroblasts and genetic defects in this pathway cause erythrocyte disorders (see e.g. Lyu et al. Metabolic regulation of erythrocyte development and disorders. Experimental Hematology, 2024. PMID: 38237718).

Given the erythroid importance of autophagy for recycling and cellular clearance (e.g. PMID: 20080761, PMID: 28483400), we hypothesize that the loss of autophagic activity in *Nprl3*$^{-/-}$ erythroblasts (Fig 3b) is a key component of the erythroid phenotype. Autophagy is induced and inhibited via protein modification (i.e. lipidation of LC3), and therefore are not always accompanied by changes in the transcriptome. Protein expression signatures that have been known to associated with altered autophagy were not detectable in our E13.5 erythroblasts. Therefore, our new omics analyses have limited potential to deepen insight into the autophagy effect. Ideally, we would assess the effect of an autophagy activator (such as spermidine) on *in vitro* erythroid culture, but unfortunately, such an approach is not achievable because of the lack of effect of Nprl3-KO on *in vitro* culture as mentioned above.

The dysregulated GSH:GSSG ratio observed from metabolomics is suggestive of redox imbalance. However, this is not accompanied by any corresponding transcriptional pathway enrichment, and individual genes involved in redox maintenance were not dysregulated: *Gr*, *Gpx*, *Nrf2*, *Nqo1, Gclc, Txndr1, Prdx4*. Proteomic analysis did show a ~3-fold increase in biliverdin reductase a (Blvra) expression in *Nprl3*$^{-/-}$ S3 erythroblasts (p=0.056, not statistically significant possibly due to low protein coverage). Blvra generates bilirubin from biliverdin, and because bilirubin is a highly potent antioxidant, this enzyme provides a cytoprotective barrier against ROS-mediated damage that may be more important than glutathione (see PMID: 12456881). We have not been able to directly measure ROS, but the lack of induction of the Nrf2 alongside an increase in Blvra protein may suggest that this bilirubin-mediated compensatory pathway operates to decrease the threat from ROS. This pathway may be of particular relevance to erythroid cells synthesizing heme as biliverdin is a heme degradation product, but this requires further investigation.

Therefore overall, our omics approaches have added to the evidence base and precision regarding disruption of glycolysis in *Nprl3*$^{-/-}$ erythroblasts, and have not altered interpretation of decreased autophagic flux. We have revised our manuscript to reflect these findings.

6. The effects on metabolism stemming from Nprl3 loss in the fetal liver would benefit greatly from a more global assessment of metabolic changes via metabolomics rather than selecting a subset of metabolites, which can introduce bias as well as cause the authors to miss key or unexpected effects that this dysregulation could have, especially in the context of this specialized cell type.

We present a subset of metabolites from a total of 48, which we appreciate is a relatively modest metabolomic study. Mature erythroblasts have an unusual metabolic profile, as they have begun the process of refining their proteome and cellular processes. We have only presented metabolites that showed significant differences, and which had sufficient abundance to measure, those which showed no difference were still helpful for refining our hypotheses. Although our metabolomics data is not global, new RNA-Seq and proteomics data have provided pertinent parallel omics to firm up our conclusion that glycolysis is dysregulated in *Nprl3*$^{-/-}$ erythroblasts (as described above). Aldolase 1 and phosphofructokinase, regulating the conversion of hexose bisphosphate to DHAP / 3-phosphoglycerate, and hexose phosphate to hexose bisphosphate, respectively, are both upregulated. Upregulation of these enzymes is consistent with the observed changes in metabolite levels. Both RNA-Seq and proteomics revealed general upregulation of glycolysis enzymes, with 4 overlapping hits.

7. Given the role of Nprl3 in responding to and controlling amino acid levels and metabolism, it is surprising that there are no significant changes in amino acid levels in these Nprl3-/- cells in Extended Figure 3. Previous studies with another GATOR1 component, DEPDC5, show dramatic differences in vivo upon GATOR1 loss (Yuskaitis et al, 2022). The authors should comment on this discrepancy.

Thank you for this interesting point for consideration. One would expect a high amino acid demand and consumption rate in conditions of mTORC1 overactivation (i.e. Gator-1 knockout). mTORC1 activation has been shown to increase amino acid uptake and synthesis through post-transcriptional control of Atf4 (PMID: 28494858). Consumption vs. replacement will determine the absolute quantity of each amino acid in a cell, likely to be influenced by factors such as starvation status, metabolic demand, cell type, etc.

For example, our new RNA-Seq data indicates upregulation of the arginine importer, *Slc7a13*, in *Nprl3*$^{-/-}$ erythroblasts. Meanwhile, metabolomics showed that intracellular arginine levels are reduced in *Nprl3*$^{-/-}$, suggesting high arginine consumption. This dataset also suggests that *Nprl3*$^{-/-}$

erythroblasts are transcriptionally programmed to boost their intracellular amino acid biosynthesis. As mentioned, one anabolic effect downstream of mTORC1 signalling is boosted translation of ATF4, which transcriptionally regulates various transporters and enzymes involved in amino acid uptake and synthesis (PMID: 28494858). Indeed, our RNA-Seq analysis indicates significant upregulation of various such ATF4 target genes in *Nprl3*$^{-/-}$ erythroblasts (*Gpt2*, *Cth*, *Slc7a11*, *Psph*, *Psat1*, *Slc1a4*).

The significantly dampened autophagy observed in *Nprl3*$^{-/-}$ erythroblasts is relevant to the question of amino acids levels. Autophagy is essential to maintain the amino acid pool, and its impairment inhibits intracellular amino acid biosynthesis. Any, and quite possibly all, of the following may therefore be occurring: amino acids are consumed at a high rate in *Nprl3*$^{-/-}$ erythroblasts because mTORC1 is constitutively activated; environmental amino acids are not sufficiently available to meet uptake demands regardless of importer expression levels; amino acid synthesis is not sufficiently supported by autophagy, which is decreased in *Nprl3*$^{-/-}$ erythroblasts.

With regard to the Yuskaitis paper, this is an interesting study, though significantly different in question and design from ours (conditional neuronal knockout of *Depdc5* vs. whole-body knockout of *Nprl3*, adult mice vs. fetal liver, whole brain lysate vs. erythroblasts, the inclusion of a starvation arm). Fasting caused a drop in the levels of several amino acids, which was greater in in *Depdc5*-cko brains. However, focussing on the 'fed' / baseline measurements, which may be most comparable to ours, only glutamine levels are different between genotypes. It may be that glutamine is less available in the brain than in the fetal liver, and therefore its overconsumption from the cortical environment is more evident. Conversely, in *Nprl3*$^{-/-}$ erythroblasts, we observe a reduction in arginine abundance. This likely highlights the differing metabolic requirements between neurons and erythroblasts. However, a more precise and effective comparison of this effect to ours, would require quantification of intracellular neuronal amino acids (as this is the specific cell type lacking Depdc5) rather than whole brain lysate. Nevertheless, this neuronal study does highlight the important of full GATOR-1 activity to modulate the mTORC1 response to nutritional stress, and we have now referred to this in the manuscript:
'Previous research indicates the importance of full GATOR-1 activity for mTORC1 regulation in conditions of nutritional stress. For example, in conditions of mTORC1 overactivation in neurons (via neuronal-specific *Depdc5*-knockout), upon fasting, the reduced cortical abundance of various amino acids was exacerbated compared to WT[52].'

8. The authors cross-bred Nprl3+/- and Nprl3+/AEKO mouse lines to assess the role of globin enhancers on Nprl3 mRNA transcription. The authors conclude that the Nprl3+/AEKO genotype "accounts for possible indirect effects of the enhancer deletions on Nprl3 expression, showing no significant difference in Nprl3 expression compared to Nprl3+/+ and Nprl3+/- lines". However, in Figure 4c, there is a clear outlier in the Nprl3+/AEKO group. Moreover, if one compares the Nprl3+/- and Nprl3AEKO/- lines, the only difference between them is the AEKO allele, and this allele leads to a large reduction in Nprl3 transcription. Given this observation, wouldn't Nprl3 transcript levels be expected to be reduced in the Nprl3+/AEKO mouse line?

Thank you for your points. Beginning with the latter regarding presence of the AEKO allele: *Nprl3* mRNA expression is not significantly different between *Nprl3*$^{+/+}$ and Nprl*3*$^{+/-}$ (Fig. 4c). This may explain the functional haplosufficiency of *Nprl3* observed in our model in terms of erythroid phenotype (Fig. 3a, Fig.1c-g). In consideration of this, in *Nprl3*$^{+/AEKO}$ (where both copies of the *Nprl3* promoter remain) even if heterozygous loss of enhancer control rendered that allele completely incapable of producing *Nprl3* (equivalent to *Nprl3*$^{+/-}$), we would not expect this to significantly reduce overall *Nprl3* mRNA expression. The difference in the case of *Nprl3*$^{+/-}$ vs. *Nprl3*$^{AEKO/-}$ is that there is only one copy of the *Nprl3* promoter on an allele where there are no enhancers. The crucial point is that *Nprl3* mRNA expression from this sole intact promoter is greatly reduced when it cannot interact with the enhancers (Fig. 4c, *Nalph*).

We performed a ROUT test that identified zero outliers in the dataset. Therefore, we feel confident in the significance indicated by the One-way ANOVA and Tukey's test.

After revisiting the raw tables to study this data, it was found that two points plotted as *Nprl3*$^{+/-}$ were actually *Nprl3*$^{+/AEKO}$ data. This has been corrected in Fig. 4c – there is no effect on the interpretation of the experiment, which remains the same as before. We have since double-checked all other graphs for such errors, none were found.

9. In Figure 4c, what accounts for the highly variable Nprl3 mRNA expression in the Nprl3+/- mouse line?

As Nprl3 responds to amino acid (and possibly iron) limitation, it is possible that its expression varies in *Nprl3*$^{+/-}$ and *Nprl3*$^{+/AEKO}$ due to variable nutritional stress on the whole litter/pregnant mother, or on particular embryos. Intrauterine position is known to affect fetal growth, possibly due to space in the uterine horn, or nutritional delivery via blood https://academic.oup.com/jas/article/92/10/4400/4702691#114706296. This would be an interesting avenue to follow up, but is beyond the scope of this study.

10. In Extended Data Figure 4B, the Pol-II ChIP-Seq shows that not only is Pol II loading impaired on the Nprl3 promoter, but also at the Hba-a1 and Hba-a2 loci in the "All enhancer KO allele". Given this observation, are the effects that the authors observe on hematopoiesis in Figure 4 and Extended Figure 4 specific to the downregulation of Nprl3 expression? This is a difficult claim to make given that hemoglobin gene transcription is also affected. To interpret their findings, the authors need to demonstrate that globin protein expression is not impaired in this mouse model or that loss of globin gene expression cannot explain the defects in red blood cell development that they observe.

Please note that the genomic make-up of the all-enhancer-KO cellular model used for PolII ChIP in Extended Data Fig. 4b (Extended Data Fig. 4c in the revised ms) is hemizygous, and so does not directly reflect the *in vivo Nprl3*$^{+/AEKO}$ or *Nalph* models. Apologies for the lack of clarity, we have now included a description of this model and a citation in the manuscript:
'Polymerase II (Pol II) ChIP-Seq also indicates reduced Pol II loading at the Nprl3 promoter when the remaining a -globin enhancers are deleted from a hemizygous a-globin locus in a cellular model (Extended Data. Fig. 4c; as described in Blayney et al.38; data available, GSE220463)').

In this pre-existing cell model from the Higgs lab, the whole a-globin-*Nprl3* locus is deleted on one allele, and all a-globin enhancers are deleted from the other (PMID: 38101409). Therefore, in this cell model, no PolII loading is possible at the alpha promoters. What these data show, is that on a single allele where *Nprl3* and its promoter are present but the enhancers are absent (as occurs in *Nalph*) PolII loading at *Nprl3* is reduced. This shows that alpha enhancers are required for optimal PolII loading of *Nprl3,* supporting the idea that alpha enhancers contribute to expression of *Nprl3* in erythroid cells.

To further assess whether the *Nalph* phenotype could be driven by effects on a-globin expression, we have now measured a-globin mRNA in all transgenic settings. a-globin expression is equivalent to WT in *Nprl3*$^{+/-}$ mice, and reduced to expected levels in *Nprl3*$^{+/AEKO}$ mice (Extended Data. Fig. 4f and g). As the enhancers are required for a-globin expression on the allele *in cis*, *Nprl3*$^{+/AEKO}$ is equivalent to the expression of 2 of 4 functional a-globin genes (such as in α^0-thalassaemia carriers). Due to the duplication of a-globin, such patients are often asymptomatic and their erythropoiesis is sufficient overall (PMID: 20507641). The same effect was observed of these mutations in the littermates of the transgenic cross, and importantly, a-globin expression was not further reduced in *Nalph* compared to *Nprl3*$^{+/AEKO}$ (Extended Data. Fig. 4h). These results exclude the possibility that a-globin transcriptional defects are driving the phenotypic observation of low S3 erythroblast number.

11. In Figure 4D, there is a modest reduction in absolute numbers of RBCs in the Nprl3+/AEKO line, especially considering the extreme loss of Nprl3 transcription observed in Figure 4C. Is this because Nprl3 protein is still present and regulation of mTORC1 signaling is still present? The authors should probe mTORC1 activity and Nprl3 protein levels across the four genotypes in Figure 4 to interpret their results.

Yes, in *Nprl3*$^{AEKO/-}$ (*Nalph*), separation of the *Nprl3* promoter from the α-globin enhancers only removes the erythroid-specific 'boosting' of *Nprl3*. Non-erythroid levels of *Nprl3* expression are maintained with the loss of enhancer regulation. With limited 'n', we do not observe statistically significant differences in pS6 signalling among the transgenic cross littermates (see below, litter represented by symbol shape) although pS6 may be increasing in S3 with progressive loss of *Nprl3* expression WT --> *Nprl3*$^{+/AEKO}$ --> *Nprl3*$^{+/-}$ --> *Nprl3*$^{AEKO/-}$, which would be consistent with more active mTORC1. Unfortunately, due to mouse breeding constraints, we have not been able to look at this in further litters. We hypothesize that *Nprl3*$^{AEKO/-}$ (*Nalph)* erythropoiesis may particularly suffer in conditions of nutritional stress, because these embryos do not have the full erythroid

capacity to temper mTORC1 and this may lead to some intra-litter and inter-litter variation in pS6 levels.

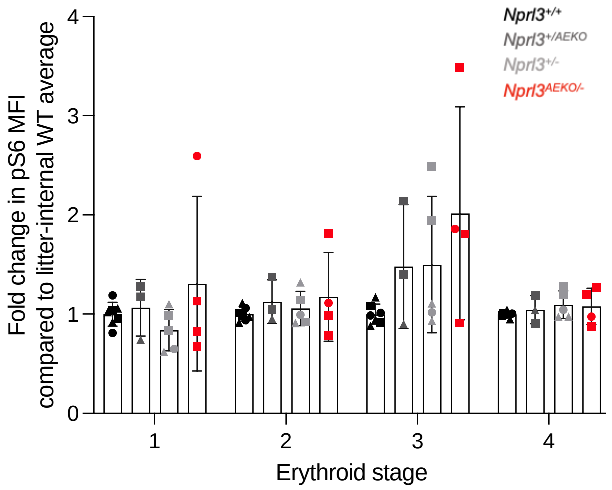

Reviewer #3 (Remarks to the Author):

This is a well written paper focused on elucidating the function of Nprl3 in erythropoiesis. Nprl3 is a gene syntenic with the alpha globin locus over a broad swath of evolution. The authors convincingly determine that Nprl3 regulates the metabolic state of erythroid precursors through the mTORC1 signalling pathway specifically in the setting of amino acid, iron and EPO deprivation. Studies are conducted both in vivo in a murine knockout model and in human CD34+-derived HSPCs cultured in vitro. Additionally, generic studies using complex mouse models indicate that the alpha globin enhancers also regulate Nprl3 expression. Taken together, this manuscript presents significant new data that the alpha globin enhancers couple Nprl3-regulated metabolism with globin gene expression during terminal stages of erythropoiesis. This serves as an intriguing example of an evolutionarily conserved enhancer sharing mechanism. The study limitations are acknowledged.

Further comments:

Major:
1. The studies of Nprl3 knock-out mouse embryos focus on the E13.5 fetal liver revealing significant defects in definitive erythropoiesis but not in concomitant HSPCs, CMP, and GMP. The relatively late in utero death of Nprl3-null embryos is consistent with a significant defect in definitive erythropoiesis. Does Nprl3 play a role in primitive erythropoiesis? Is it expressed in primitive erythroblasts? Are Nprl3 knock-out mouse embryos anemic at E12.5, i.e., before significant numbers of definitive cells have entered the circulation? Is Nprl3 regulation connected to the alpha-globin enhancers that regulate zeta-globin?

Thank you for the interesting thought. We used CellAtlas.io to check the relative erythroid expression of *Nprl3* at a primitive time point. Please see the plots below extracted from CellAtlas.io (developed by the Haniffa lab, with searchable transcriptional expression in the human yolk sac, https://app.cellatlas.io/yolk-sac/dataset/52/scatterplot). This shows that *Nprl3* is expressed relatively highly in yolk sac primitive erythroid cells compared to other lineages, which may be suggestive of functional importance (as we find with definitive erythroblasts).

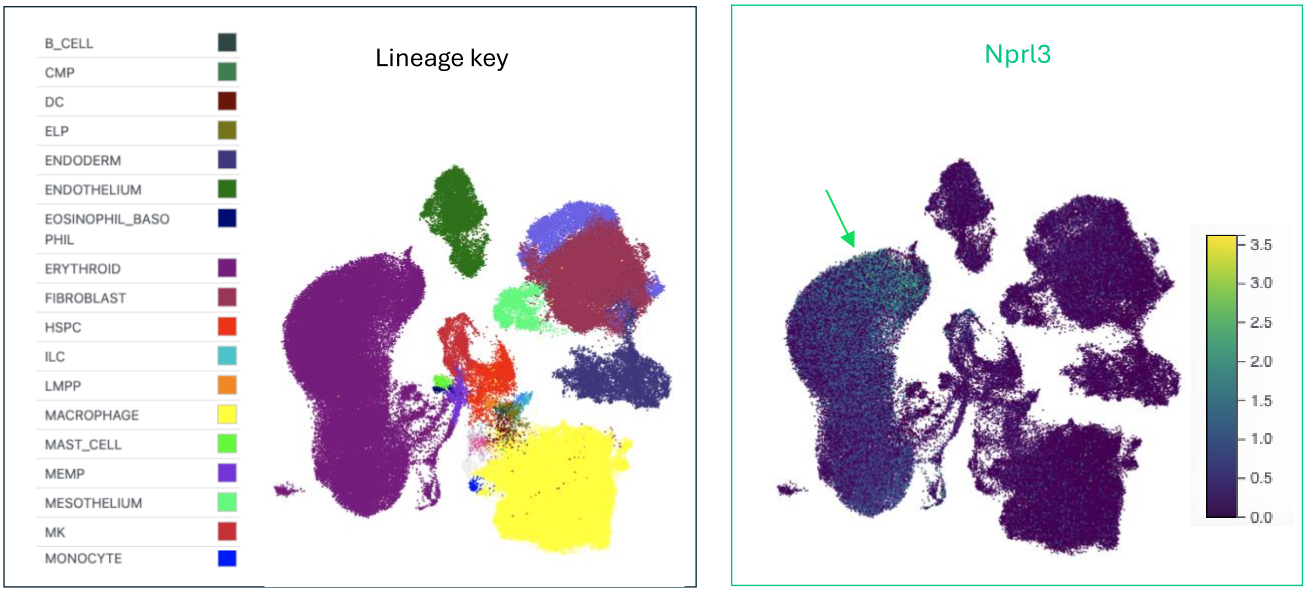

Another interactive scRNA-Seq dataset from E12.5 fetal liver includes a defined population of residual primitive erythroblasts (EryP (green arrow)), which do express *Nprl3*. Generated by Ceccacci et al, 2023 (https://ececcacci.shinyapps.io/LiverE12/).

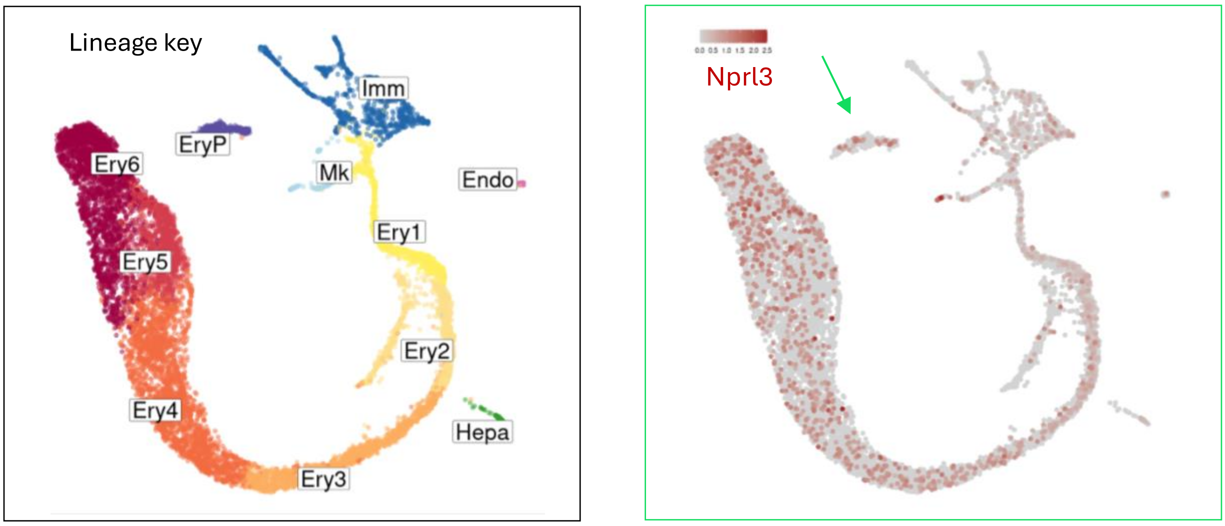

The anticipation that the metabolic repercussions of $Nprl3^{-/-}$ would also influence primitive erythropoiesis is interesting, and we have now mentioned this in the discussion. Finally, zeta-globin is indeed regulated by the same enhancer cluster which regulates a-globin and *Nprl3* (PMID: 25126789).

Unfortunately, due to constraints in our breeding facility we were unable to acquire specific information on E12.5 fetal livers to assess anemia.

Whilst the study of primitive erythropoiesis is beyond the scope of our study, we acknowledge that this is an interesting consideration, and have now mentioned the plausible primitive role of Nprl3 in our discussion:
'While our work focusses on definitive erythropoiesis, *Nprl3* is also expressed by primitive erythroid cells, and it is plausible that Nprl3 also influences metabolic regulation of primitive erythropoiesis[41,42].'

Minor:
1. Second paragraph, "erythroblasts undergo rapid haemoglobinisation followed by high autophagic and proteosomal activity", and first paragraph of the Discussion describing anabolic and subsequent catabolic phases. Is there evidence that these are two separate phases over the ~3 terminal erythroblast cell divisions or do they occur simultaneously during terminal erythroid differentiation?

Yes, key findings describe intense, cell-defining anabolic and catabolic events of different cell types within erythropoiesis. For example, we know that mTORC1-driven haemoglobinisation (PMID: 25872869) launches and surges in basophilic erythroblasts (with their defining basophilic cytoplasm formed of high ribosomal density for globin production, driven by increased levels of haem (PMID: 30530752; mouse)). We also know that autophagy/mitophagy peak in the later orthochromatic erythroblasts and reticulocytes (PMID: 18539900; mouse), in order to clear the red cell of proteins and organelles and leave an enucleated sack that is 95% haemoglobin. Of course, baseline levels of autophagy and proteasomal activity will also exist during the haemoglobinisation phase, as autophagy is required for macromolecular turnover to support protein synthesis.
As a result of answering this point, we also edited the first paragraph of the Discussion for clarity.

2. In contrast to the findings in the fetal of Nrpl3 knock-out mice, in chimeric adult mice Nprl3-null cells do not contribute well to the broad HSPC compartment, suggesting defects at that level in HSC-derived hematopoiesis, as well as in terminal erythropoiesis. Despite this defect and the severe defect in BasoE and subsequent stages of erythropoiesis (Extended Data, Fig. 1f), there are normal numbers of CFU-E/ProE. Can the authors speculate how this might be possible?

Thank you for noting this point. We hypothesise that the erythroid differentiation block at the basophilic stage (observed in both the *Nprl3*$^{-/-}$ fetal liver and chimeric mice, Extended Data Fig. 1a/d) causes an accumulation of undifferentiating proerythroblasts, increasing the number of *Nprl3*$^{-/-}$ cells counted in the CFU-E/ProE gate.

Manuscript text:
'Irrespective of the potential role for *Nprl3*$^{-/-}$ in HSCs, the accumulation of *Nprl3*$^{-/-}$ pro-erythroblasts and subsequent defect in terminal erythropoiesis supports a role for *Nprl3*$^{-/-}$ in erythroid differentiation.'

3. Nprl3 knock-out erythroid cells have decreased glutathione levels. Do they contain increased ROS levels or do other antioxidant pathways compensate?

RNA-Seq does not indicate changes in other antioxidant pathways. We checked genes that control antioxidant levels, such as *Gr*, *Gpx*, *Nrf2*, *Nqo1, Gclc, Txndr1,* and Prdx4, and found no differential regulation between WT and *Nprl3*$^{-/-}$. However, proteomic analysis revealed that biliverdin reductase a (Blvra) was upregulated in *Nprl3*$^{-/-}$ S3 erythroblasts (though not significantly, p=0.056). Blvra generates bilirubin from biliverdin, and because bilirubin is a highly potent antioxidant, this enzyme provides a cytoprotective barrier against ROS-mediated damage that may be more important than glutathione (see https://www.pnas.org/doi/full/10.1073/pnas.252626999). We have not been able to directly measure ROS, but the lack of induction of the Nrf2 pathway concomitant with the ~3-fold increase in Blvra protein indicates that this bilirubin-mediated compensatory pathway may be operating to decrease the threat from ROS. This pathway may be of relevance to erythroid cells synthesizing heme as biliverdin is a heme degradation product, this is an interesting finding for further study.

4. The study of Nprl3 levels and Nrlp3 promoter-enhancer interactions in erythroid cells from the fetal livers of transgenic mice support the regulation of Nprl3 expression by the alpha-globin enhancers. The decreased hemoglobin and MCV of Nprl3+/AEKO mice are certainly consistent with alpha-thalassemia. Are there comparative data available from alpha-thalassemia mice lacking 2 of the 4 alpha-globin genes?

Thank you, this is a helpful comparison. Please see the table below, containing peripheral haematological indices for alpha-thalassemia mice, including Hba$^{th\text{-}J}$ ($^{- -}$/^{aa}) from The Jackson Laboratory (PMID: 8781443). These seem to be consistent with those of Nprl3$^{+/AEKO}$ mice (Extended Data Fig. 4e), also shown below for ease.

Table 1. Hematologic Data of α1 Globin Gene Knockout Mice

Mouse	Genotype	Hb (g/dL)	MCV (fL)	MCH (pg)	Reticulocytes (%)
Normal	$\alpha\alpha/\alpha\alpha$	14.65 ± 1.34	56.71 ± 0.99	16.93 ± 0.43	2.58 ± 0.84
Heterozygote	$^*\alpha/\alpha\alpha$	13.74 ± 0.80	50.97 ± 1.88	15.11 ± 0.55	2.33 ± 1.50
Homozygote	$^*\alpha/^*\alpha$	13.20 ± 0.69	45.05 ± 1.22	12.15 ± 0.50	11.33 ± 0.58
Hba^{th-J}	$--/\alpha\alpha$	12.75 ± 1.82	46.10 ± 2.51	13.45 ± 1.21	5.02 ± 0.82

All values are expressed as mean ± SD. In each group all hematologic indices were calculated based on five mice except the reticulocyte value of the homozygous group, in which only three mice were used.

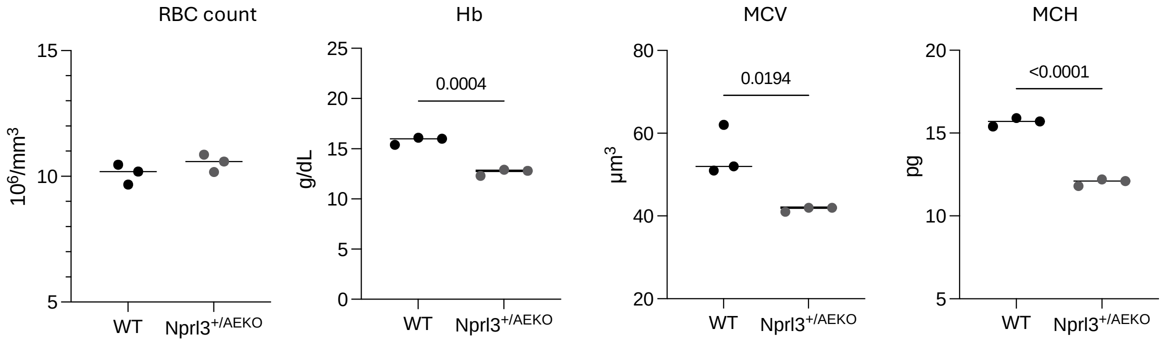

REVIEWER COMMENTS

Reviewer #1 (Remarks to the Author):

Overall, this is an interesting study that identifies “housekeeping” and erythroid specific functions for the mTORC1 inhibitor Nprl3 in hematopoiesis. In general, I am satisfied with the authors efforts to address reviewer comments. However, not all of the comments could be addressed, in part due to technical difficulties, unavailable or faulty reagents and the complexities of mTORC1 signaling. For many experiments, the numbers of mice analyzed are relatively low and there is considerable phenotype variability, possibly due to variable metabolic conditions, as the authors note in their rebuttal. The authors provide evidence for accelerated glycolysis and impaired autophagy, both of which are known consequences of mTORC activation. However, there is no experimental evidence that either of these abnormalities contribute to the observed impairment in erythropoiesis. The authors also note that studying mTORC signaling and its effects on cells in vitro is difficult due to variables that are difficult to control for. Several reviewer comments/critiques are answered in the rebuttal but not addressed in the revised manuscript. To address these issues, I suggest that the authors add a paragraph to the discussion reviewing limitations of the study, including some of the questions/problems cited by the reviewers.

Thank you. We have extended our limitations paragraph to discuss more of the questions raised by reviewers:

We would like to note some limitations of this study. The relative contributions of underlying causative mechanisms of *Nprl3*$^{-/-}$ erythropoietic failure at S3 are not defined. Plausible, and possibly linked, mechanisms include: autophagic inhibition constricts macromolecular availability for biosynthesis (recycling) preventing maintenance of cellular health (defective clearance); and unsustainable energetic imbalance represented by the dysregulation of glycolysis. Pharmacological interventions during pregnancy to test the contribution of these processes to the erythropoietic phenotype were difficult to interpret due to effects of drugs on both mother and fetus. When cultured *ex vivo*, *Nprl3*$^{-/-}$ fetal liver cells differentiate comparably to WT, likely due to superfluous nutrient availability in *in vitro* culture conditions, so that rescue experiments testing (for example) the effects of suppressing glycolysis were not interpretable. For some experiments on *Nalph,* sample numbers were limited by breeding constraints and limited material available per fetal liver, per pregnancy. Similarly, it would have been informative to describe true promoter-derived *Nprl3* expression among all haematopoietic lineages using ddPCR, but we were limited by the availability of non-erythroid cellular material per fetal liver. In our metabolomic study, the number of metabolites detected is relatively modest. This is in part because mature erythroblasts have an unusual metabolic profile, in the process of refining their proteome and cellular processes. Deeper study of metabolic pathway dysfunction in Nprl3 deficiency would require metabolic tracer assays. However, RNA-Seq and proteomic data provide pertinent parallel ‘omics, and together the datasets indicate a general upregulation of glycolytic enzymes. Another limitation is lack of an available orthogonal method of separating *Nprl3* from the a-globin enhancers. The complexity of having two essential genes and their regulatory elements

interspersed at one locus made this experiment particularly challenging. *Nalph,* and its internal littermate controls result from years of breeding and genetic manipulation[9,20,38], and cannot currently be recapitulated by *in vitro* methods. Finally, the human erythroid flow cytometry does not feature erythroblast-stage specific analysis, as surface markers of human erythroid maturation change along a continuum, meaning that defining distinct populations is challenging.

A few additional comments/suggestions:

1. The abstract could better explain one of the author's major points -- that Nprl3 is required for all hematopoietic lineages (possibly due to a requirement in hematopoietic stem/progenitor cells), but an extra "boost" from the alpha globin enhancers is required specifically for erythroid cells.

Thank you. We have updated the abstract to highlight this point. However, please note that while Nprl3 is required for the formation of all haematopoietic lineages in a competitive setting (i.e. the bone marrow chimaera), at steady state in the fetal liver, non-erythroid haematopoietic cell types (and CMP/GMP/MEP progenitors) are all unaffected by *Nprl3*$^{-/-}$. This highlights an erythroid-specific importance of Nprl3.

Abstract:
Development of red blood cells from progenitors requires profound reshaping of both gene expression and metabolism[1–3]. How these processes are coupled is unclear*. Nprl3,* an inhibitor of mTORC1, has remained in synteny with the a-globin genes for >500 million years[4], and harbours the majority of the a-globin enhancers[5]. However, whether Nprl3 itself serves an erythroid role is unknown. Here, we show that Nprl3 is a key regulator of erythroid metabolism. Using Nprl3-deficient fetal liver and adult competitive bone marrow - fetal liver chimaeras, we show that Nprl3 is required for sufficient erythropoiesis. Loss of Nprl3 elevates mTORC1 signalling, suppresses autophagy and disrupts erythroblast glycolysis. Human *NPRL3*-knockout erythroid progenitors produce fewer enucleated cells and demonstrate dysregulated mTORC1 signalling in response to nutrient availability and erythropoietin. Finally, we show that the a-globin enhancers upregulate *Nprl3* expression, and that this activity supports optimal erythropoiesis. Haematopoietic progenitors rely on tonic expression of baseline *Nprl3*, while erythropoiesis requires 'boosted' *Nprl3* expression, bestowed by the erythroid-specific a-globin enhancers. Therefore, we propose that the anciently conserved linkage of *Nprl3*, a-globin and their associated enhancers has coupled metabolic and developmental control in erythroid cells.

2. Change text accompanying Figure 4f-h (line 268, page 6) to: "This viable level of alpha-globin mRNA is not further reduced by the co-presence of Nprl3+/- (as in Nalph) in cis…"

Changed.

3. Line 331, page 7. Authors state: "Therefore, it is likely that disruption of glycolysis due to mTORC1 overactivation contributes to the Nprl3-/- erythroid differentiation defect". However, glycolysis thought to be upregulated, not disrupted. This point should be clarified I the text. How might activation of glycolysis inhibit erythropoiesis? Is there evidence in the

literature to support this? Have the authors actually measured glycolysis in the mutant erythroid cells?

Evidence from the 'omics studies indicates that some (but not all) enzymes involved in core glycolytic pathways are upregulated and concentrations of some (but not all) intermediates in glycolysis are also raised. Metabolic tracer assays would be required to determine whether pathway flux is upregulated. Our view is that inappropriate upregulation of components of glycolysis relative to wild-type represents a disruption in the normal functioning of this pathway. Perhaps 'dysregulated' is a more suitable term, which we have used to replace 'upregulated' in-text.

We can suggest mechanisms by which elevated glycolysis might inhibit erythropoiesis. Over-production of some glycolytic metabolites might shunt them into other anabolic pathways such as the pentose phosphate or serine glycine pathway. *Nprl3*$^{-/-}$ cells may be unable to keep up energy demands of increased flux of carbon into these anabolic pathways ultimately resulting in a cytotoxic unbalanced metabolic state. The phenotype may parallel that of tumour cells with constitutively activated PI3K, whose 'unrelenting demand for energy, exacerbated by inefficient ATP production by glycolysis and the absence of an alternative energy source generated by autophagy, leads to a necrotic pathway to cell death' (PMID: 17251378). Thus, inability to fulfil metabolic demands can force cell death, and we know from our study that autophagy is impaired in *Nprl3*$^{-/-}$ cells. However, this remains hypothetical and we are reluctant to speculate on this in the manuscript due to limited relevant literature.

Reviewer #2 (Remarks to the Author):

My main concerns were addressed by the authors, and I commend them for doing so.

First, I was concerned about the lack of validation that Nprl3 levels were reduced in their Nprl3-/- mouse line. I appreciate the authors' efforts to find an antibody that works, and their data that Nprl3 mRNA levels are greatly decreased in Ext. Data Figure 4 is an important addition.

There is high variability on the effect of mTORC1 activity with the Nprl3-/- mice in Figure 2, but the information the authors now include about the litter identity clarifies confusion, especially in combination with the other findings of how Nprl3 loss affects responsiveness to arginine and leucine deprivation.

The authors presented a more unbiased view of the metabolic effects of Nprl3 loss with RNA seq, which more convincingly pinpoint the defects in glycolysis than only targeted metabolomics. I understand the difficulties of performing more ideal rescue experiments and mechanistic work in their system, and I hope that the authors will follow-up these findings in another model system that is subject to less variability and will be more amenable to biochemical and genetic manipulation.

Finally, the authors addressed the validity of their Nalph model in Figure 4. Their corrections of misplotted data with necessary measurements of alpha globin levels make a stronger case that the effects observed on S3 erythroblast numbers are not due to an effect in alpha globin expression but rather the reduction of Nprl3 in this model.

We thank the reviewer for their positive reflection.

Reviewer #3 (Remarks to the Author):

The authors have very effectively responded to the critiques and Issues raised by the reviewers of the initial submission. Importantly, they have performed additional experiments and added new data both to the primary and to the extended figures. The authors have also appropriately modified the text of the manuscript to reflect the new data. Additionally, the text (particularly the Discussion) has been modified in response to other points/questions raised in the reviews. Overall, these additions and changes further strengthen an already strong paper.

This manuscript presents significant new data that the alpha globin enhancers couple Nprl3-regulated metabolism with globin gene expression during terminal stages of erythropoiesis. This serves as an intriguing example of an evolutionarily conserved enhancer sharing mechanism.

We thank the reviewer for their support of our work.

REVIEWERS' COMMENTS

Reviewer #1 (Remarks to the Author):

The authors have responded to most of my queries and the queries from other reviewers. A few questions poised by the reviewers remain unanswered due to various problems including difficulties producing mice, lack of reagents, etc. It might be helpful to add a section in the discussion about open questions and limitations of the current study.

Thank you. Please see the discussion section, which includes a paragraph outlining the limitations of the study (388-423), including the limited availability of transgenic mice and their cellular material.
